# Severe mental illness, race/ethnicity, multimorbidity and mortality following COVID-19 infection: nationally representative cohort study

**DOI:** 10.1192/bjp.2023.112

**Published:** 2023-11

**Authors:** Jayati Das-Munshi, Ioannis Bakolis, Laia Bécares, Jacqueline Dyer, Matthew Hotopf, Josephine Ocloo, Robert Stewart, Ruth Stuart, Alex Dregan

**Affiliations:** Department of Psychological Medicine, Institute of Psychiatry, Psychology and Neuroscience, King's College London, UK; Centre for Society and Mental Health, King's College London, UK; and South London & Maudsley NHS Trust, London, UK; Centre for Implementation Sciences, Department of Health Services and Population Research, Institute of Psychiatry, Psychology and Neuroscience, King's College London, UK; Department of Global Health and Social Medicine, King's College London, UK; NHS England, London, UK; Department of Psychological Medicine, Institute of Psychiatry, Psychology and Neuroscience, King's College London, UK; and South London & Maudsley NHS Trust, London, UK; Department of Psychological Medicine, Institute of Psychiatry, Psychology and Neuroscience, King's College London, UK

**Keywords:** Mortality, COVID-19, severe mental illness, multimorbidity, ethnicity

## Abstract

**Background:**

The association of COVID-19 with death in people with severe mental illness (SMI), and associations with multimorbidity and ethnicity, are unclear.

**Aims:**

To determine all-cause mortality in people with SMI following COVID-19 infection, and assess whether excess mortality is affected by multimorbidity or ethnicity.

**Method:**

This was a retrospective cohort study using primary care data from the Clinical Practice Research Database, from February 2020 to April 2021. Cox proportional hazards regression was used to estimate the effect of SMI on all-cause mortality during the first two waves of the COVID-19 pandemic.

**Results:**

Among 7146 people with SMI (56% female), there was a higher prevalence of multimorbidity compared with the non-SMI control group (*n* = 653 024, 55% female). Following COVID-19 infection, the SMI group experienced a greater risk of death compared with controls (adjusted hazard ratio (aHR) 1.53, 95% CI 1.39–1.68). Black Caribbean/Black African people were more likely to die from COVID-19 compared with White people (aHR = 1.22, 95% CI 1.12–1.34), with similar associations in the SMI group and non-SMI group (*P* for interaction = 0.73). Following infection with COVID-19, for every additional multimorbidity condition, the aHR for death was 1.06 (95% CI 1.01–1.10) in the SMI stratum and 1.16 (95% CI 1.15–1.17) in the non-SMI stratum (*P* for interaction = 0.001).

**Conclusions:**

Following COVID-19 infection, patients with SMI were at an elevated risk of death, further magnified by multimorbidity. Black Caribbean/Black African people had a higher risk of death from COVID-19 than White people, and this inequity was similar for the SMI group and the control group.

People living with severe mental illness (SMI) experience a stark 15–20 year reductions in life expectancy compared with the general population.^1^ Before the COVID-19 pandemic, it had been noted that this had remained persistently elevated over decades, been observed across international contexts^1^ and worsened over time.^2^ These inequalities have been observed irrespective of race/ethnicity.^3,4^ The onset of the global COVID-19 pandemic in 2020 heralded further concerning trends affecting these groups, with investigators noting that people with SMI had experienced a further excess risk of death from COVID-19 and other causes at the start of the pandemic,^5^ and were at a higher risk, in general, of hospital admission and mortality,^6–8^ with concerns that the presence of underlying health conditions potentially contributed to the excess risk.^8^

The COVID-19 pandemic has also been highlighted as potentially exacerbating pre-existing inequalities, particularly in relation to race/ethnicity.^9–12^ The intersection of race/ethnicity with the presence of SMI on COVID-19-related outcomes is yet unclear. Multimorbidity is known to be more prevalent in some racially minoritised groups.^13^ In addition, there have been concerns that racially minoritised groups experienced delays in access to testing, protective personal equipment and vaccination, and similar concerns have been reported in people living with SMI. The interplay of SMI in racially minoritised groups, contributing to the excess risk of death following COVID-19 infection, are also yet unknown.

## Aims

With this in mind, we conducted a cohort study, using nationally representative data from primary care. The cohort was followed from the start of the COVID-19 pandemic in 2020 until April 2021, and covered two periods of peak COVID-19 mortality across the UK.

We assessed the following three research questions:

- Was the risk of death higher in people living with SMI if they contracted COVID-19?
- Was the excess risk of death magnified by underlying long-term conditions/multimorbidity?
- Was any observed excess risk attributable to race/ethnicity?

## Method

The present study implemented a prospective cohort design within the Clinical Practice Research Datalink (CPRD) Aurum database, one of the world's largest primary care electronic medical databases. The CPRD Aurum database includes prospective historical data from family practices in England and Northern Ireland, with around 13.5 million currently active patients (20% of the UK population). Patients included in the CPRD Aurum database are broadly representative of the UK population in terms of geographical distribution, area-level deprivation, age and gender.^14^ The CPRD includes detailed information on clinical diagnoses and symptoms, therapies, referrals, laboratory tests and sociodemographic variables.

### Ethics and consent statement

The authors assert that all procedures contributing to this work comply with the ethical standards of the relevant national and institutional committees on human experimentation and with the Helsinki Declaration of 1975, as revised in 2008. All procedures involving human patients were approved by the Independent Scientific Advisory Committee of CPRD (protocol number 20_069R1). All data sent to the CPRD are anonymised and therefore consent is not required.

### Exposures

For this study, we included all patients with an SMI (e.g. schizophrenia, schizoaffective disorders, bipolar disorders) who were aged 5 years and over, with a definitive SARS-CoV-2 infection recorded between 1 February 2020 and 31 March 2021. SMI was defined as the presence of at least one diagnostic record entry for schizophrenia, schizoaffective disorder, bipolar disorder or other affective disorder with psychosis, during the study period. These are recorded according to the Systematised Nomenclature of Medicine – Clinical Terms (SNOMED) system, and are generally diagnosed in secondary mental healthcare, with the diagnosis provided to primary care clinicians. We used CPRD-documented codes to identify a COVID-19 diagnosis event in the database (see Supplementary Material available at https://doi.org/10.1192/bjp.2023.112). These codes include a broad set of diagnosis criteria, such as COVID-19 confirmed by laboratory test or clinically, asymptomatic COVID-19 and codes denoting diseases (e.g. cardiomyopathy, encephalopathy) caused by SARS-CoV-2 infection. Asymptomatic COVID-19 diagnoses were based on a medical event recorded by a general practitioner in the patient medical file, likely following a positive COVID-19 laboratory test. For consenting practices, COVID-19 data are linked to the national testing system, such as the Public Health England (PHE) Second Generation Surveillance System, PHE COVID-19 Hospitalisation in England Surveillance System and Intensive Care National Audit and Research Centre data on COVID-19 intensive care admissions. Ongoing work aims to validate CPRD-coded COVID-19 events with those in the linked data-sets.

For this study we selected two groups: (a) a population control group, comprising patients without a diagnosis of SMI, who had a definitive positive COVID-19 test during the study period (designated as the control group/‘no SMI group’); and (b) a group of patients diagnosed with SMI who tested positive for COVID-19 during the study period (designated as the ‘SMI group’).

All patients in the study were required to have at least 12 months of follow-up before the index date (date of first recorded COVID-19 infection) – for some patients this was as early as 1 February 2019 – for the COVID-19 pandemic in the UK (defined as the 1 February 2020). The control group included all eligible patients without SMI with a COVID-19 diagnosis during the study period. Patients were followed up on until the earliest of the following dates: date of registration termination, date of death, last data collection (for patients that transferred out of a CPRD practice) or the study end date (31 March 2021). To reduce the risk of reverse causation between SMI and COVID-19 diagnoses, we excluded patients with an SMI diagnosis after a COVID-19 infection. The study was approved by the Independent Scientific Advisory Committee (reference 20_069R1).

### Outcome measure

The primary outcome measure was all-cause mortality, identified with information on date of death recorded in primary care records. Mortality data recording in CPRD has been found to be highly accurate, with 99% of date of deaths being within 30 days of CPRD-registered dates, when compared with the Office for National Statistics death registration data.^15^

### Covariates

The study selected key variables documented in the literature to be associated with the risk of SMI and all-cause mortality. These variables included demographic factors such as age (continuous), gender (female/male), geographical regions, self-ascribed ethnicity and area deprivation. Because of small sample sizes in the SMI population, ethnicity had to be re-coded into broader ethnic groups. These included a ‘White’ reference group, which included White British, White Irish and White other groups; a ‘Black’ ethnicity group, which included Black Caribbean, Black African and Black Other groups; a ‘South Asian’ group, which included Indian, Pakistani, Bangladeshi and Asian other groups; and a ‘mixed’ ethnicity group. We also included the ‘other’ ethnicity group and people of ‘unknown’ ethnicity, with the latter category encoded as ‘missing’ in complete-case models and then subsequently multiply imputed, with other missing fields (see below for details). Geographical region was defined by National Health Service (NHS) administrative areas and used to classify patients into large geographical regions (e.g. London and South-East England, the Midlands, North-West England, South-West England and Northern Ireland), to capture regional variations in mortality experiences across models. Area deprivation, a composite measure of area-level income, education, employment, skills and training, crime, health and disability, and housing, was derived from patient-level quintiles of the 2015 English Index of Multiple Deprivation,^16^ at the Lower Super Output Area level, which are UK geographical small areas, comprising a mean of 650 households. We also included information on intensive care unit admission as a marker of COVID-19 infection severity, as well as immunological and corticosteroid drug therapy. Multimorbidity that was linked with an increased risk of COVID-19 infection and related mortality was also included. This included hypertension, myocardial infarction, heart disease, ischaemic stroke, type 2 diabetes mellitus, cancer, liver disease, kidney disease, chronic obstructive pulmonary disease, asthma, autoimmune disorders (e.g. rheumatoid arthritis, lupus, vasculitis, colitis, Crohn's disease, psoriasis), substance use disorders (both alcohol and drug), epilepsy, depression, anxiety, eating disorders, gastroesophageal reflux disorders and dementia. We used these diagnoses to derive a continuous measure of multimorbidity, by counting the total number of conditions recorded before COVID-19 infection for each patient. Finally, we included clinical variables such as body mass index (BMI) (categorised as underweight (BMI < 17.5 kg/m^2^), normal (BMI 18–25 kg/m^2^), overweight (BMI 25–30 kg/m^2^) or obese (BMI ≥30 kg/m^2^)) and smoking (never, former and current). Covariates were selected based on the information closest to the study start date on 1 February 2020. A full list of codes for covariates is available on request from the authors.

### Statistical analysis

We used descriptive statistics to summarise differences in baseline characteristics between cases and the comparison groups.

We employed multivariable Cox proportional hazards regression analyses to estimate differences in all-cause mortality between patients with an SMI with a positive COVID-19 test, and the comparison group. Participants were followed up from the index date for COVID-19 until the earliest of date of death, transferred out date or study end date (31 March 2021). We added covariates sequentially into the models, starting with age and gender, and then we included the full set of all other covariates. To adjust for potential clustering effects by practice, we used cluster-robust s.e., with the *vce(robust)* option in Stata version 17 for Windows (StataCorp LLC, College Station, TX, USA). Schoenfeld residual plots were used to examine the proportionality hazards assumptions and no violations were observed. To assess for potential minority ethnic differences, regional differences and differences by the presence of multimorbidity for all-cause mortality, we also included interaction terms between SMI caseness with ethnicity, with geographical region variables and multimorbidity.

We imputed missing data on clinical and demographic variables (BMI, smoking, Index of Multiple Deprivation), based on missing-at-random assumptions, using multiple imputation with chained equations (ten imputations). The imputation model included all covariates and the outcome measure, as well as interaction effects between SMI status with ethnicity, region and multimorbidity. Given that the missing-at-random assumption may not hold within routinely collected data, we also used complete-case analysis to confirm the findings from imputed estimates, as sensitivity analyses.

Additional analyses were performed to calculate all-cause mortality at two different time points, to broadly coincide with rising infection and dates of UK Government lockdown and policy changes. These included 1 February 2020 to 30 September 2020 (date of the first UK COVID-19 infection wave) and 1 October 2020 to 31 March 2021 (date of the second UK COVID-19 infection wave). Also, because the SMI and non-SMI groups differed on some key variables, we performed additional sensitivity analysis that matched SMI cases with five population controls on the number of long-term conditions (multimorbidity) in addition to the demographic variables (e.g. age gender, general practice, index date for COVID-19). Because fatalities are rare among children and adolescents, in additional sensitivity analyses we excluded patients with SMI who had a relevant SMI diagnosis before 18 years of age. To deal with missing data on covariates (BMI, blood pressure, smoking, ethnicity), we conducted multiple imputation with multiple chained equations. We imputed ten data-sets and included all study variables, including outcome, exposures and covariates, as well as planned interactions. Sensitivity analyses with 20 imputed data-sets suggested no differences in the precision of estimate with larger iterations. All data analyses were conducted in Stata version 17 for Windows, with a two-sided *P* < 0.05 set as the significance threshold.

## Results

**Fig. 1:**
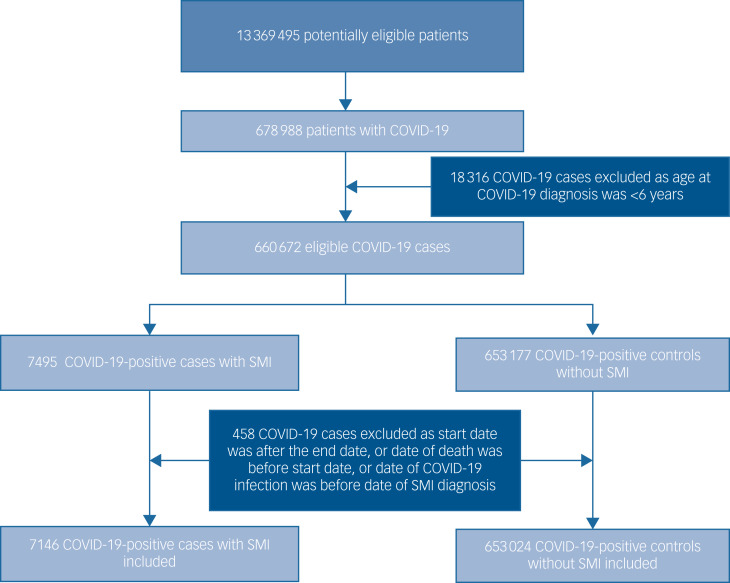
Flow diagram illustrating the selection of patients into the study. SMI, severe mental illness.

The total study population included 7146 patients with an SMI and a definitive COVID-19 diagnosis, and 653 024 people in the non-SMI/control group. The selection of patients into the study is presented in the flow chart (Fig. 1). Demographic, clinical and comorbidity information at baseline across the four groups are detailed in Table 1. Patients with an SMI were older than controls. A higher proportion of patients with an SMI who contracted COVID-19 were obese, current smokers and of Black Caribbean/Black African ethnicity, compared with the other groups, and had multimorbidity.

**Table 1:** Baseline demographic and clinical characteristics for the sample with positive COVID-19 test results, by severe mental illness status

	No SMI group, *n* (%)	SMI group, *n* (%)
Sample size	653 024	7146
Age, years, mean (s.d.)	43 (20)	55 (19)
Gender, female	360 937 (55)	4088 (57)
Body mass index, kg/m^2^		
Optimal (17.5–24.9)	185 523 (28)	1851 (26)
Underweight (<17.5)	24 781 (4)	223 (3)
Overweight (25–29.9)	169 662 (26)	2041 (29)
Obese (≥30)	151 753 (23)	2757 (38)
Missing	121 295 (19)	270 (4)
Smoker		
Never	372 224 (57)	3272 (46)
Former	71 833 (11)	1728 (24)
Current	130 605 (20)	2100 (29)
Missing	78 363 (12)	42 (1)
Area deprivation^a^		
Least deprived	108 702 (17)	786 (11)
Second	112 968 (17)	1075 (15)
Third	116 685 (18)	1210 (17)
Fourth	139 900 (21)	1575 (22)
Most deprived	147 329 (23)	2077 (29)
Missing	27 440 (4)	423 (6)
Race/ethnicity		
White British/Irish/White other	160 611 (25)	4777 (67)
Black Caribbean/Black African/Black other	22 829 (3)	415 (6)
Indian/Pakistani/Bangladeshi	57 934 (9)	690 (10)
Mixed ethnicity	7630 (1)	122 (2)
Other	235 657 (36)	362 (5)
Missing	168 363 (26)	776 (11)
UK regions		
London	143 665 (22)	1688 (24)
North-West England	139 938 (23)	1634 (23)
North-East England	41 800 (7)	458 (6)
West Midlands	121 784 (17)	1233 (17)
East Midlands	32 651 (5)	319 (4)
South-East England	117 544 (18)	1255 (18)
South-West England	58 772 (8)	465 (7)
Northern Ireland	774 (<1)	17 (<1)
Missing	413 (<1)	73 (1)
Multimorbidity/long-term health conditions		
Hypertension	99 156 (15)	1806 (25)
Myocardial infarction	10 319 (2)	190 (3)
Heart disease	21 028 (3)	248 (3)
Ischemic stroke	7386 (1)	492 (7)
Diabetes	51 192 (8)	1336 (19)
Cancer	20 947 (3)	666 (9)
Liver disease	17 283 (3)	410 (6)
Kidney disease	29 902 (5)	931 (13)
Chronic obstructive pulmonary disease	24 876 (4)	677 (9)
Asthma	112 018 (17)	1501 (21)
Autoimmune diseases	39 717 (6)	651 (9)
Substance use disorders	8850 (1)	835 (12)
Epilepsy	10 251 (2)	607 (9)
Depression	139 588 (21)	3783 (53)
Anxiety	142 329 (22)	3017 (42)
Eating disorders	11 144 (2)	287 (4)
Gastroesophageal reflux disorder	16 591 (3)	462(6)
Dementia	15 768 (2)	265 (4)
Immunological drug therapy	22.035 (34)	2806 (39)
Corticosteroid drugs	84 895 (13)	1074 (15)
Admission to intensive care unit	659 (0.10)	26 (0.34)

SMI, severe mental illness.

a. Area deprivation according to the Index of Multiple Deprivation.

**Fig. 2:**
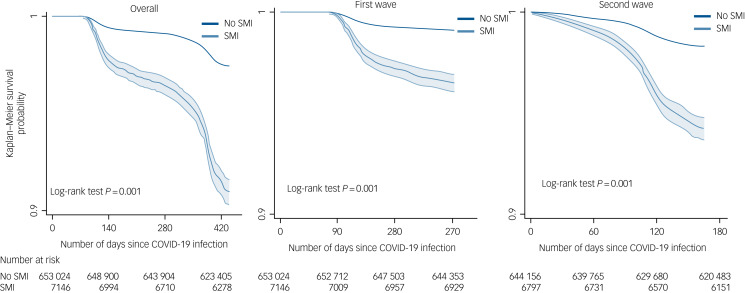
Survival probability following COVID-19 infection by severe mental illness (SMI) status, over the first two waves of the COVID-19 pandemic. For ease of understanding, the graphs use a cut-off point of 0.9.

The period of the study spanned two waves of COVID-19 infection in the UK. Figure 2 displays survival probabilities in people living with SMI compared with population controls, following COVID-19 infection. Over both waves, the SMI group was more likely to die following COVID-19 infection, compared with the population control group. In the UK, deaths from COVID-19 began to increase from March/April 2020, reflected in the graphs for the first wave and the ‘overall’ survival probabilities (Fig. 2), with no deaths initially recorded in the first 60 days following an infection, and very few deaths between 60 and 90 days after infection. The graphs indicated a steeper decline in survival probabilities in the SMI population during both pandemic waves. These trends were replicated in covariates-adjusted survival probabilities (Supplementary Fig. 2).

**Fig. 3:**
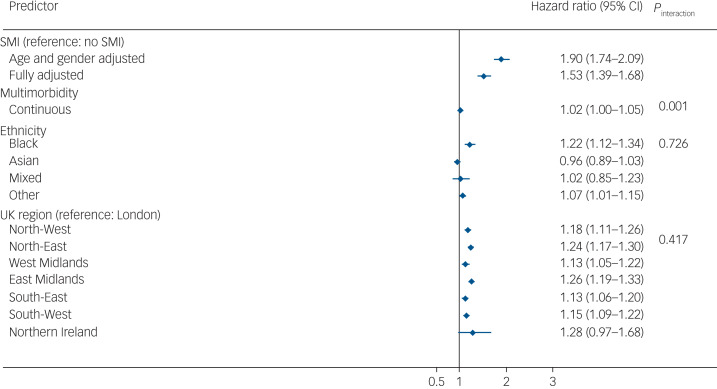
Hazard ratios for the association of severe mental illness (SMI) with all-cause mortality after COVID-19 infection. Displayed estimates are from imputed estimates. Fully adjusted models were adjusted for age, gender, multimorbidity, ethnicity, UK region and all comorbidities from Table 1. The hazard ratio includes a combination of independent and interaction effects. The hazard ratio for SMI represents the effect of SMI on all-cause mortality in the absence of multimorbidity; the interaction effect represents the additional (multiplicative) effects of SMI in the presence of multimorbidity. Effects sizes for ethnicity and UK region variables represent the fully adjusted model analysis.

In the imputed results (Fig. 3), patients living with SMI who tested positive for COVID-19 were at an increased risk of all-cause mortality in age- and gender-adjusted models (adjusted hazard ratio (aHR) 1.90, 95% CI 1.74–2.09), which remained elevated after further adjusting for multimorbidity, race/ethnicity, UK region, area deprivation, BMI and smoking status (aHR = 1.53, 95% CI 1.39–1.68). There was no evidence of an interaction between SMI and race/ethnicity, although Black Caribbean/Black African groups experienced a higher risk of all-cause mortality following COVID-19 infection compared with the White reference group. There was no evidence of interaction by region of residence either; however, all-cause mortality risks were elevated outside of London (Fig. 2). In analyses broken down by waves of infection (wave 1 spanning February to September 2020 and wave 2 spanning from October 2020 to April 2021), after adjusting for all covariates, the aHR for all-cause mortality was 1.71 (95% CI 1.48–1.99; *P* < 0.001) during the first wave of the pandemic, and 1.40 (95% CI 1.25–1.57; *P* < 0.001) during the second wave in the SMI group compared with the non-SMI control group. Estimates from complete-case models were similar to the imputed models (see Supplementary Fig. 2).

In analyses assessing an interaction between SMI status and multimorbidity (as a continuous variable, ranging from 0 to 11 conditions), there was evidence (*P* = 0.001) in support of a statistical interaction. After taking into account all confounders, the aHR for death following COVID-19 infection was 1.06 (95% CI 1.01–1.10) in the SMI stratum and 1.16 (95% CI 1.15–1.17) in the non-SMI control group stratum, in complete-case models. These estimates were similar to those from imputed models (SMI sample, imputed models: aHR = 1.08, 95% CI 1.04–1.12; non-SMI control group, imputed models: aHR = 1.15, 95% CI 1.14–1.16). These results indicate that the additional (multiplicative) effect of multimorbidity, although statistically significant in both groups, was greater in the non-SMI group compared with the SMI group.

In sensitivity analyses that matched SMI and non-SMI groups on number of long-term conditions (Supplementary Fig. 4), we observed similar findings to the main study results (albeit with smaller effect sizes), increasing confidence in the robustness of the evidence. Finally, analyses excluding patients living with SMI who were younger than 18 years at the time of SMI diagnosis produced similar results to the main findings (Supplementary Fig. 3). The only notable difference was the larger effect size for the independent effect of multimorbidity (aHR = 1.19, 95% CI 1.18–1.20) on all-cause mortality.

## Discussion

Using data from a nationally representative cohort study of 660 517 individuals followed from the start of the COVID-19 pandemic for over a year, we found that people living with SMI had a substantially elevated risk of death following COVID-19 infection. As the study spanned two phases of the pandemic in the UK, we were able to document a steeper increase in mortality for patients living with SMI during the first wave compared with their counterparts in the general population. We observed a sharp drop in survival probability at around 400 days in both SMI and non-SMI groups (Fig. 1), but particularly in the SMI group. This finding might be explained by the steep rise in COVID-19-related deaths from mid-January 2021 to mid-March 2021, as per official Office of National Statistics data (https://www.ons.gov.uk/peoplepopulationandcommunity/healthandsocialcare/conditionsanddiseases/articles/coronaviruscovid19latestinsights/overview).

Of note, the SMI population experienced a greater and lasting risk for all-cause mortality compared with the non-SMI populations during the second wave of the pandemic. The presence of multimorbidity modified the risk of death in the SMI group, accentuating this heightened risk further. We also observed a main effect of ethnicity, so that Black Caribbean/Black African people in the sample were more likely to experience death following infection with the SARS-CoV-2 virus, compared with the White reference group. Our analyses highlighted further inequalities in the mortality risk by region of residence, although this risk was experienced in a similar manner across SMI and non-SMI groups.

Our findings extend previous work confirming that people living with SMI experienced an excess risk of death during the COVID-19 pandemic.^5,8^ All-cause mortality risk was substantially elevated in this group, compared with the general population. In people with SMI, excess mortality was elevated because of a rise in deaths following COVID-19 infection; simultaneously, excess mortality from all other (non-COVID-19) causes, compared with the general population, continued to be a concern.^5^ Further, it documented that this risk was not restricted to early stages of the COVID-19 pandemic, but rather continued to adversely affect life expectancy in the SMI population. However, it is noteworthy that a lower aHR for mortality was observed in the second wave in this study. This may reflect UK vaccine roll out, which commenced in early January 2021, during the second wave of infections. People with SMI were considered to be at a heightened risk of serious illness and death following COVID-19 infection, and were prioritised at the outset for vaccination. Although it has been noted that these groups were more likely to decline vaccination, in general, overall vaccination rates were high in people with SMI. Concerns have previously been raised that people living with SMI experience profound social exclusion and social isolation, as well as reduced access to healthcare, evident before the pandemic.^3,17^

Our findings highlight the importance of the presence of co-occurring long-term conditions (multimorbidity), which further accentuated the risk of death in people living with SMI, as well as in the non-SMI/control group. The risk of death was less magnified by the presence of multimorbidity in the SMI sample compared with the non-SMI/ control group, but this should be interpreted in the context of people with SMI already having a greater excess risk of death compared with the non-SMI group, with multimorbidity further accentuating this gap. The smaller aHR for multimorbidity in the SMI group compared with the non-SMI group implies that all-cause mortality was possibly explained by the SMI rather than by other morbidities in the SMI group. The difference may also be because of differences in the management or control of multimorbidity between the SMI and non-SMI groups (e.g. better access to care/services during the COVID-19 lockdown).

Given the observation that in the general population, COVID-19 has been associated with an excess risk of death in racially minoritised populations in the UK,^9^ USA^10–12^ and elsewhere,^18,19^ we sought to explicitly assess whether death in these groups was more evident in people living with SMI. Our assessment of race/ethnicity indicated that Black Caribbean/Black African patients had a higher risk of death following SARS-COV-2 infection, compared with the White reference group. We observed these ethnic inequalities to a similar extent across the SMI and non-SMI control groups. This is consistent with previous work, and may suggest that the effect of living with an SMI has a profound ‘ceiling’ effect, leading to marked inequalities in mortality and physical health, which are then experienced to a similar extent across all racially minoritised groups.^3^

From a public health perspective, our study has emphasised the need for early and timely preventative interventions (e.g. vaccination) for the SMI population. Future studies are needed to disentangle the complex biological and psychosocial factors, and healthcare pathways, that have led to the greater mortality rates in the SMI population.^20^ For instance, we recently documented that the presence of frailty resulted in a fourfold increase in risk of all-cause mortality in people with SMI.^21^ A recent expert prioritisation exercise highlighted a range of risk factors contributing to excess mortality in people with SMI. This included the challenges that people with SMI experience when accessing care which may be fragmented or of poorer quality, the impact of stigmatising processes such as diagnostic overshadowing, and the role of socioeconomic disadvantage. Much routine care was withdrawn during the first and second waves of the pandemic, and face-to-face healthcare provision changed to remote/telecare, which may have further affected the quality of care received by people with SMI during the pandemic. Multimorbidity, including the presence of alcohol and substance use disorders, also likely affected the severity of the SMI and the risk of COVID-19 infection, as well as mortality outcomes. Co-existing diseases may result in exacerbated inflammatory responses in the SMI population^22^ or may reduce the functioning of specific body organs (e.g. lung, kidney). To prevent similar inequalities in future, it is essential to both improve the health of the SMI population and better integrate different clinical services for the SMI population during similar outbreaks.^23^

### Strengths and limitations

Although we were able to undertake a comprehensive assessment of COVID-19 infection in the cohort by using linked data sources, including information from national testing and surveillance systems, a limitation is that the presence of COVID-19 infection may have been underdiagnosed or underreported in the records. This would have led to observed associations being estimated closer to the null. However, despite this, the impact of COVID-19 on mortality risk was substantially elevated across the sample. It is possible that there were some undiagnosed SMI cases in the control sample; however, given previous work^24^ indicating that the recording of SMI in primary care records is either slightly higher or similar to community epidemiological samples, it is likely that this would have had a minimal impact on observed associations. A further limitation was that we did not have information on cause of death, limiting our ability to assess cause-specific differences in mortality. Previous work has suggested that people living with SMI have experienced elevated risk of death from both COVID-19 and other causes over the course of the pandemic.^5^ Despite being a relatively large sample, our assessment of ethnic inequalities may have been hampered by smaller sample sizes for the SMI group and incomplete data. Although our analyses adjusted for a wide range of long-term conditions known to be associated with mortality, we cannot exclude the possibility of residual confounding. Sensitivity analyses for unmeasured confounding, however, confirm that this bias had a limited effect on our findings. For example, the E-value was 2.02 for the hazard ratio and 1.84 for the upper confidence interval, indicating that the evidence for causal association was reasonably strong even under the assumption that the study confounding control was relatively poor (the hazard ratios for study confounders were <1.3, with the exception of intensive care unit admission). Also, we cannot exclude the possibility of reverse causation between COVID-19 infection and SMI. This limitation may have under- or overestimated the true association between SMI and all-cause mortality in our population. To minimise the effect of reverse causation, we excluded all SMI diagnoses after a COVID-19 infection. Our study did not account for multiple COVID-19 infections, which may have affected the results pending unequal distribution in this rate between the SMI and non-SMI groups. Since our study timeline covered only the first two waves, this issue may be of lesser concern for the present findings. Furthermore, we had to group together some of the race/ethnicity categories because of small sample sizes, and this would have affected our analyses by potentially masking differences within groups. Further, we have not considered the potential mediating effect of the vaccination rollout programme on the study outcome. The vaccination rollout programme in the UK started around February/March 2021, and may have only minimally affected our study results.

Strengths of the study include the prospective cohort design, using a sample representative of the UK population according to age, gender and ethnicity,^25^ thus suggesting good generalisability and external validity. This is because most of the general population in the UK (98%) is under primary care and healthcare remains free at point of contact. In addition, the use of linked data would have meant that key exposures (COVID-19 tests/ diagnoses) were captured accurately in the health records,^25^ albeit with the caveats mentioned above. UK primary care electronic health records have been noted as having high levels of recording with good accuracy for clinical diagnoses,^26^ particularly since the introduction of financial incentives to manage chronic diseases.^25^ It is possible that people living with SMI may have been less likely to be in contact with primary care; however, previous studies have found that the incidence of SMI in UK primary care samples are comparable to the incidence reported in community samples,^27^ suggesting that the issue of selection bias is less of a concern.

In conclusion, the findings from this study indicate that people living with SMI have experienced large and substantial inequalities in mortality outcomes during the COVID-19 pandemic, which are further magnified through the presence of underlying health conditions and have been experienced to a similar extent across racialised populations. These inequalities were observed across multiple waves of the pandemic, which will likely exacerbate the life expectancy gap between people with SMI and the general population.

## Data Availability

The analytical codes used in this study are available online (https://github.com/alexdregan/CPRD). The clinical codes are available from the corresponding author, A.D., on request. Access to data is available only once approval has been obtained through the individual constituent entities controlling access to the data. The primary care data can be requested via application to the Clinical Practice Research Datalink.

## Supplementary material

For supplementary material accompanying this paper visit https://doi.org/10.1192/bjp.2023.112. click here to view supplementary material
