# Severe mental illnesses, race/ ethnicity, multimorbidity and mortality following COVID-19 infection: Nationally representative cohort study

**DOI:** 10.1192/bjp.2023.112

**Published:** 2023-11

**Authors:** Jayati Das-Munshi, Ioannis Bakolis, Laia Bécares, Jacqui Dyer, Matthew Hotopf, Josephine Ocloo, Robert Stewart, Ruth Stuart, Alex Dregan

**Affiliations:** 1 Department of Psychological Medicine, Institute of Psychiatry, Psychology & Neurosciences, King’s College London, London, UK; 2 Centre for Society and Mental Health, King’s College London, London, UK; 3 South London & Maudsley NHS Trust, London, UK; 4 Centre for Implementation Sciences, Department of Health Services & Population Research, Institute of Psychiatry, Psychology & Neuroscience, King’s College London, London, UK; 5 Department of Global Health and Social Medicine, King’s College London, London, UK; 6 NHS-England, UK

## Abstract

**Background:**

The association of COVID-19 with death in people with severe mental illnesses (SMI), and associations with multimorbidity and ethnicity are unclear.

**Aims:**

To determine all-cause mortality in people with SMI following COVID-19 infection and assess whether excess mortality is affected by multimorbidity or ethnicity.

**Method:**

This was a retrospective cohort study using primary care data from the Clinical Practice Research Database (CPRD) from February 2020 to April 2021. Cox proportional hazards regression was used to estimate the impact of SMI on all-cause mortality during the first two-waves of the COVID-19 pandemic.

**Results:**

Among 7,146 people with SMI (56% female), there was a higher prevalence of multimorbidity compared to the non-SMI control group (N=653,024, 55% female). Following COVID-19 infection, the SMI group experienced a greater risk of death compared to controls (adjusted hazard ratio (aHR)=1.53, 95% confidence interval (CI), 1.39-1.68). Black Caribbean/ Black African people were more likely to die from COVID-19 compared with the White reference group (aHR: 1.22 (95% CI, 1.12-1.34), with similar associations in the SMI group and non-SMI group (p-value for interaction=0.73). Following infection with COVID-19, for every additional multimorbidity condition, the aHR for death was 1.06 (95% CI 1.01, 1.10) in the SMI stratum and 1.16 (95% CI: 1.15, 1.17) in the non-SMI stratum (p-value for interaction=0.001).

**Conclusions:**

Following COVID-19 infection, patients living with SMI were at an elevated risk of death, further magnified by multimorbidity. Black Caribbean/ Black African people had a higher risk of death from COVID-19 relative to the White reference group, this inequity was similar for the SMI and the control group.

## Introduction

People living with severe mental illnesses (SMI) experience stark 15-20 year reductions in life expectancy relative to the general population^1^. Prior to the COVID-19 pandemic, it had been noted that this had remained persistently elevated over decades, had been observed across international contexts^1^ and worsened over time^2^. These inequalities have been observed irrespective of race/ ethnicity^3 4^. The onset of the global COVID-19 pandemic in 2020 heralded further concerning trends impacting these groups, with investigators noting that people with SMI had experienced a further excess risk of death from COVID-19 and other causes at the start of the pandemic^5^, and were at a higher risk, in general, of hospitalisation and mortality^6–8^, with concerns that the presence of underlying health conditions potentially contributed to the excess risk^8^.

The COVID-19 pandemic has also been highlighted as potentially exacerbating pre-existing inequalities, particularly by race/ ethnicity^9–12^. The intersection of race/ ethnicity with the presence of severe mental illness on COVID-19 related outcomes is yet unclear. Multimorbidities are known to be more prevalent in some racially minoritized groups^13^. In addition, there have been concerns that racially minoritized groups experienced delays in access to testing, Protective Personal Equipment (PPE) and vaccination, similar concerns have been reported in people living with SMI. The interplay of SMI in racially minoritized groups, contributing to the excess risks of deaths following COVID19 infection, are also yet unknown.

With this in mind, we conducted a cohort study, using nationally representative data from primary care. The cohort was followed from the start of the COVID-19 pandemic in 2020 until April 2021 and covered two periods of peak COVID-19 mortality across the UK.

We assessed the following three research questions:

1. Was the risk of death higher in people living with SMI if they contracted COVID-19?
2. Was the excess risk of death magnified by underlying long-term conditions/ multimorbidities?
3. Was any observed excess risk attributable to race/ethnicity?

## Methods

The present study implemented a prospective cohort design within the Clinical Practice Research Datalink (CPRD) Aurum database, one of the world’s largest primary care electronic medical databases. The CPRD Aurum database includes prospective historical data from family practices in England and Northern Ireland, with around 13.5 million currently active patients (20% of the UK population). Patients included in the CPRD Aurum database are broadly representative of the UK population in terms of geographical distribution, area-level deprivation, age, and sex.^14^ The CPRD includes detailed information on clinical diagnoses and symptoms, therapies, referrals, laboratory tests, and socio-demographic variables.

### Ethics and consent statement

The authors assert that all procedures contributing to this work comply with the ethical standards of the relevant national and institutional committees on human experimentation and with the Helsinki Declaration of 1975, as revised in 2008. All procedures involving human patients were approved by the Independent Scientific Advisory Committee of CPRD (protocol no. 20_069R1). All data sent to the CPRD is anonymised and therefore consent is not required.

#### Exposures

For this study, we have included all SMI (e.g., schizophrenia, schizoaffective disorders, bipolar disorders) patients aged 5 years and over with a definitive SARS-CoV-2 infection recorded between 01/02/2020 to 31/03/2021. SMI was defined as the presence of at least one diagnostic record entry for schizophrenia, schizoaffective disorder, bipolar disorder, or other affective disorder with psychosis during the study period. These are recorded according to the Systematised Nomenclature of Medicine – Clinical Terms (SNOMED) system and are generally diagnosed in secondary mental healthcare, with the diagnosis provided back to primary care clinicians. We used CPRD documented codes to identify a COVID-19 diagnosis event in the database (Appendix). These codes include a broad set of diagnosis criteria, such as COVID-19 confirmed by laboratory test or clinically, asymptomatic COVID-19, as well as codes denoting diseases (e.g., cardiomyopathy, encephalopathy) caused by SARS-CoV-2 infection. Asymptomatic Covid-19 diagnoses were based on a medical event recorded by GP in patient medical file, likely following a positive Covid-19 lab test. For consenting practices, COVID-19 data is linked to the national testing system, such as the Public Health England (PHE) Second Generation Surveillance System (SGSS), PHE COVID-19 Hospitalisation in England Surveillance System (CHESS), and the Intensive Care National Audit and Research Centre (ICNARC) data on COVID-19 intensive care admissions. Ongoing work aims to validate CPRD-coded COVID-19 events with those in the linked datasets.

For this study we selected two groups:

1) A population control group, patients without a diagnosis of SMI, who had a definitive positive COVID-19 test during the study period (designated as the control group/ “no SMI group”);
2) A group of patients diagnosed with SMI who tested positive for COVID-19 during the study period (designated as the “SMI group”).

All patients in the study were required to have at least 12 months of follow-up prior to the index date (date of first recorded COVID-19 infection) – for some patients for as early as 01/02/2019 - for the COVID-19 pandemic in the UK (defined as the 1^st^ of February 2020). The control group included all eligible non-SMI patients with a COVID-19 diagnosis during the study period. Patients were followed-up until the earliest of: date of registration termination; date of death; last data collection (for patients that transferred out of a CPRD practice); or the study end date (31/03/2021). To reduce the risk of reverse causation between SMI and COVID-19 diagnoses, we have excluded patients with an SMI diagnosis after a COVID-19 infection. The study was approved by the Independent Scientific Advisory Committee (Ref 20_069R1).

#### Outcome measure

The primary outcome measure was all-cause mortality, identified using information on date of death recorded in primary care records. Mortality data recording in CPRD has been found to be highly accurate, with 99% of date of deaths being within 30 days of CPRD registered dates, when compared with the Office for National Statistics (ONS) death registration data.^15^

#### Covariates

The study selected key variables documented in the literature to be associated with the risk of SMI and all-cause mortality. These variables included demographic factors such as age (continuous), gender (female/male), geographical regions, self-ascribed ethnicity, and area deprivation. Due to small sample sizes in the SMI population, ethnicity had to be re-coded into broader ethnic groups. These included a ‘White’ reference group, which included White British, White Irish and White Other groups, a ‘Black’ ethnicity group which included Black Caribbean, Black African and Black Other groups, a ‘South Asian’ group which included Indian, Pakistani, Bangladeshi and Asian ‘Other’ groups, and a ‘Mixed’ ethnicity group. We also included the ‘Other’ ethnicity group, and people of ‘Unknown’ ethnicity- with the latter category encoded as ‘missing’ in complete case models and then subsequently multiply imputed, with other missing fields (see below for details). Geographical region was defined by NHS administrative areas and used to classify patients into large geographical regions (e.g., London and South East England, Midlands, North West England, South West England, and Northern Ireland), to capture regional variations in mortality experiences across models. Area deprivation, a composite measure of area-level income, education, employment, skills and training, crime, health and disability, and housing, was derived using patient-level quintiles of 2015 English Index of Multiple Deprivation (IMD)^16^, at the Lower Super Output Area (LSOA) level, which are UK geographical small areas, comprising a mean of 650 households. We have also included information on intensive care unit (ICU) admission as a marker of COVID-19 infection severity, as well as immunological and corticosteroids therapy. Multimorbidities linked with an increased risk of COVID-19 infection and related mortality were also included. These included hypertension, myocardial infarction, heart disease, ischaemic stroke, type 2 diabetes mellitus, cancer, liver disease, kidney disease, chronic obstructive pulmonary disease (COPD), asthma, autoimmune disorders (e.g., rheumatoid arthritis, lupus, vasculitis, colitis, Crohn’s disease, psoriasis), substance abuse disorders (both alcohol and drug abuse), epilepsy, depression, anxiety, eating disorders, gastroesophageal reflux disorders (GERD) and dementia. -We have used these diagnoses to derive a continuous measure of multimorbidity, by counting the total number of conditions recorded prior to Covid-19 for each patient. Finally, we included clinical variables such as body mass index (BMI) (categorised as underweight (BMI<17.5), normal (BMI 18-25), overweight (BMI 25-30), and obese (BMI 30+)) and smoking (never, former, and current). Covariates were selected based on the information closest to the study start date on 1^st^ February 2020. A full list of codes for covariates is available on request from the authors.

#### Statistical analysis

We used descriptive statistics to summarise differences in baseline characteristics between cases and the comparison groups.

We employed multivariable Cox proportional hazards regression analyses to estimate differences in all-cause mortality between SMI patients with a positive COVID-19 test, and the comparison group. Participants were followed-up from the index date for COVID-19 until the earliest of death, transferred out date, or study end date (31/03/2021). We added covariates sequentially into the models starting with age and gender, and then we included the full set of all other covariates. To adjust for potential clustering effects by practice, we used cluster-robust standard errors, with the *vce(robust)* option in Stata. Schoenfeld residual plots were used to examine the proportionality hazards assumptions and no violations were observed. To assess for potential minority ethnic, regional differences and differences by the presence of multimorbidities for all-cause mortality, we also included interaction terms between SMI caseness with ethnicity, with geographical region variables and multimorbidity. We imputed missing data on clinical and demographic variables (BMI, smoking, IMD), based on missing at random (MAR) assumptions, using multiple imputation with chained equations (10 imputations). The imputation model included all covariates and the outcome measure, as well as interaction effects between SMI status with ethnicity, region, and multimorbidity. Given that the MAR assumption may not hold within routinely collected data, we have also used complete case analysis to confirm the findings from imputed estimates, as sensitivity analyses.

Additional analyses were performed to calculate all-cause mortality at two different time points, to broadly coincide with rising infection and dates of UK government lock-down and policy changes. These included 1^st^ of February 2020 to 30^th^ of September 2020 (date of first UK COVID-19 infection wave) and 1^st^ of October 2020 to 31^st^ of March 2021 (date of second UK COVID-19 infection wave). Also, because the SMI and non-SMI groups differed on some key variables, we have performed additional sensitivity analysis that matched SMI cases with 5 population controls on the number of long-term conditions (multimorbidity) in addition to the demographic variables (e.g., age gender, general practice, index date for Covid-19). Because fatalities are rare among children and adolescents, in additional sensitivity analyses we have excluded SMI patients with a relevant SMI diagnosis before the age of 18. To deal with missing data on covariates (BMI, BP, smoking, ethnicity), we have conducted multiple imputation using multiple chained equation. We have imputed 10 datasets and included all study variables, including outcome, exposures, and covariates, as well as planned interactions. Sensitivity analyses using 20 imputed datasets suggested no differences in the precision of estimate with larger iterations. All data analyses were conducted in Stata version 17, with a two-sided p<0.05 set as the significance threshold.

## Results

**Figure 1:**
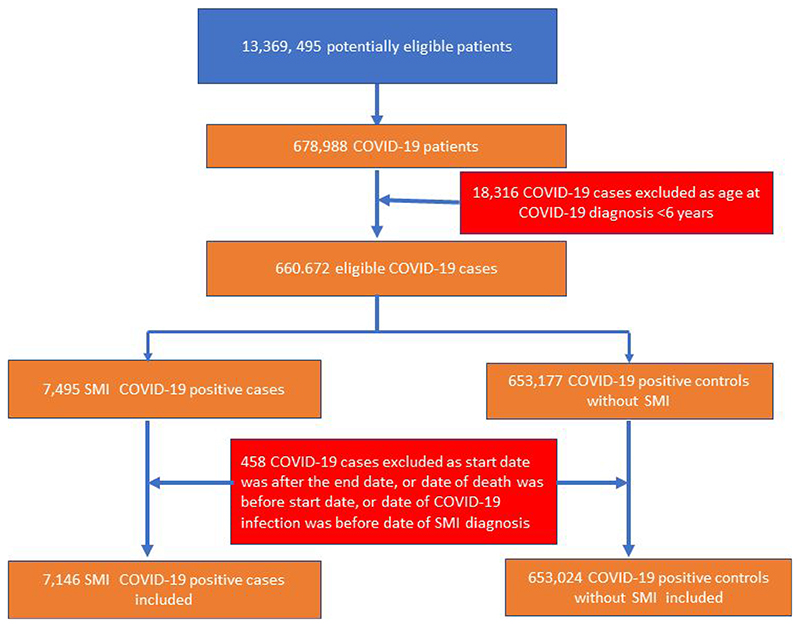
Flow diagram illustrating the selection of patients into the study.

The total study population included 7,146 patients with SMI and a definitive COVID-19 diagnosis and 653,024 in the non-SMI/ control group. The selection of patients into the study is presented in the flow chart (Figure 1). Demographic, clinical, and comorbidity information at baseline across the four groups are detailed in Table 1. SMI patients were older than non-SMI control groups. A higher proportion of SMI patients who contracted COVID-19 were obese, current smokers, and were of Black Caribbean/ Black African ethnicity, relative to the other groups, and had multimorbidity.

**Figure 2:**
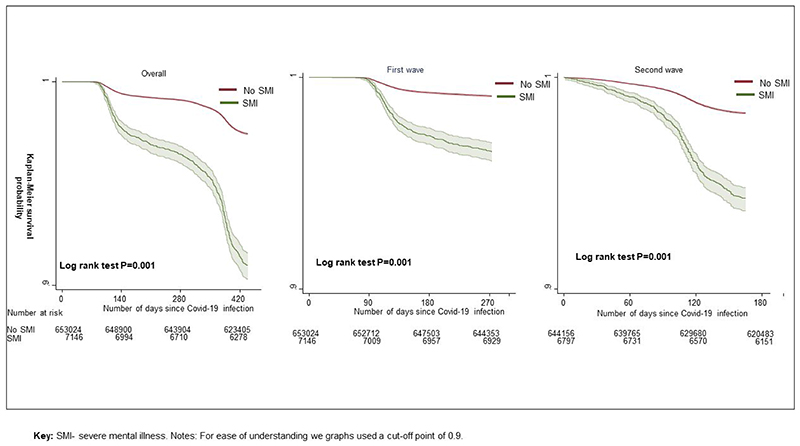
Survival probability following COVID-19 infection by severe mental illness (SMI) status, over the first two waves of the COVID-19 pandemic.

The period of the study spanned two waves of COVID-19 infection in the UK. Figure 2 displays survival probabilities in people living with SMI relative to population controls, following COVID-19 infection. Over both waves the SMI group were more likely to die following COVID-19 infection, relative to the population control group. In the UK, deaths from COVID-19 began to increase from March/ April 2020, reflected in the graphs for the first wave and the ‘overall’ survival probabilities_(Figure 2), with no deaths initially recorded in the first 60 days following an infection, and very few deaths between 60-90 days post-infection. The graphs indicated a steeper decline in survival probabilities in the SMI population, during both pandemic waves. These trends were replicated in covariates-adjusted survival probabilities (Figure S2, Supplementary material).

**Figure 3:**
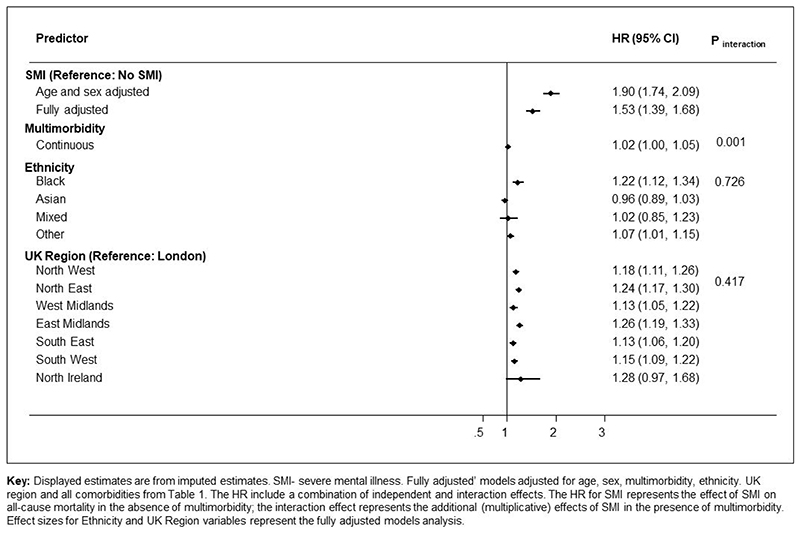
Hazard Ratios for the association of severe mental illnesses (SMI) with all-cause mortality, following COVID-19 infection

In the imputed results (Figure 3), SMI patients who tested positive for COVID-19 were at an increased risk of all-cause mortality in age and sex adjusted models (adjusted Hazard Ratios (aHRs) 1.90 (95% CI: 1.74, 2.09) which remained elevated after further adjusting for multimorbidity, race/ ethnicity, regions, area deprivation, BMI and smoking status (aHR: 1.53 (95% CI: 1.39-1.68)). There was no evidence of an interaction between SMI and race/ ethnicity, although Black Caribbean/ Black African groups experienced a higher risk of all-cause mortality following COVID-19 infection compared to the White reference group. There was no evidence of interaction by region of residence either, however all-cause mortality risks were elevated outside of London (Figure 2). In analyses broken down by waves of infection (wave 1 spanning Feb-Sept 2020 and wave 2 from Oct 2020 to April 2021), after adjusting for all covariates, the aHR for all-cause mortality was 1.71 (95% CI: 1.48, 1.99; p<0.001) during the first wave of the pandemic, and 1.40 (95% CI: 1.25, 1.57; p<0.001) during the second wave in the SMI group relative to the non-SMI control group. Estimates from complete case models were similar to the imputed models (see supplementary figure 2).

In analyses assessing an interaction between SMI status and multimorbidities (as a continuous variable, ranging from 0 to 11 conditions) there was evidence (p=0.001) in support of a statistical interaction. After taking into account all confounders, the adjusted Hazard Ratio of death in the SMI stratum was 1.06 (95% CI: 1.01, 1.10) and in the non-SMI control group stratum was 1.16 (95%CI: 1.15, 1.17) following COVID-19 infection, in complete case models. These estimates were similar to those from imputed models (in the SMI sample, imputed models, aHR: 1.08 (95% CI: 1.04, 1.12) and in the non-SMI control group stratum, imputed models, aHR: 1.15 (95% CI: 1.14, 1.16)). These results indicate that the additional (multiplicative) impact of multimorbidity, while statistically significant in both groups, was greater in the non-SMI group compared to the SMI patients.

In sensitivity analyses that matched SMI and non-SMI groups on number of long-term conditions (Figure S4, Supplementary material), we have observed similar findings to the main study results (albeit the smaller effect sizes) increasing confidence in the robustness of the evidence. Finally, analyses the excluded SMI patients younger than 18 years at the time of SMI diagnosis produced similar results to the main findings (Figure S3, Supplementary material). The only notable difference was the larger effect size for the independent effect of multimorbidity (1.18, 95% CI: 1.17, 1.19) on all-cause mortality.

## Discussion

Using data from a nationally representative cohort study of 660,517 individuals followed from the start of the COVID-19 pandemic for over a year, we found that people living with SMI had a substantially elevated risk of death following COVID-19 infection. As the study spanned two phases of the pandemic in the UK, we were able to document a steeper increase in mortality for the SMI patients during the first wave compared to their counterparts in the general population. We have observed a sharp drop in survival probability around 400 days in both SMI and non-SMI groups (Figure 1), particularly the former. This finding might be explained by the steep rise in COVID-19 related deaths from mid-January 2021 to mid-March 2021, as per official Office of National Statistics data (https://www.ons.gov.uk/peoplepopulationandcommunity/healthandsocialcare/conditionsanddiseases/articles/coronaviruscovid19latestinsights/overview, page accessed on 01/07/2023).

Of note, the SMI population experienced a greater and lasting risk for all-cause mortality relative to the non-SMI populations during the second wave of the pandemic. The presence of multimorbidity modified the risk of death in the SMI group, accentuating this heightened risk further. We also observed a main effect of ethnicity, so that Black Caribbean/ Black African people in the sample were more likely to experience death following infection with the SARS-CoV-2 virus, relative to the White reference group. Our analyses highlighted further inequalities in the mortality risk by region of residence, although this risk was experienced in a similar manner across SMI and non-SMI groups.

Our findings extend previous work confirming that people living with SMI experienced an excess risk of deaths during the COVID-19 pandemic^5 8^. All-cause mortality risk was substantially elevated in this group, compared with the general population. In people with SMI, excess mortality was elevated due a rise in deaths following COVID-19 infection, whilst at the same time, excess mortality from all other (non-COVID-19) causes, relative to the general population, continued to be a concern^5^. Further, it documented that this risk was not restricted to early stages of the COVID-19 pandemic, but rather continued to adversely impact on the SMI population life expectancy. It is noteworthy however that a lower adjusted hazard ratio for mortality was observed in the second wave in this study. This may reflect UK vaccine roll out which commenced in early January 2021, during the second wave of infections. People with SMI were considered to be at a heightened risk of serious illness and death following COVID-19 infection and were prioritised at the outset for vaccination. Although subsequently it has been noted that these groups were more likely to decline vaccination, in general overall vaccination rates were high in people with SMI. Concerns have previously been raised that people living with SMI experience profound social exclusion and social isolation as well as reduced access to healthcare, evident prior to the pandemic^3^ ^17^. Our findings highlight the importance that the presence of co-occurring long-term conditions (multimorbidity), which further accentuated the risk of death in people living with SMI, as well as in the non-SMI/ Control group. The risk of death was less magnified by the presence of multimorbidity in the SMI sample compared to the non-SMI/ control group, however this should be interpreted in the context of people with SMI already having a greater excess risk of death relative to the non-SMI group, with multimorbidity further accentuating this gap. The smaller aHR for multimorbidity in the SMI group compared to the non-SMI group imply that all-cause mortality was possibly explained by the SMI rather than by other morbidities in the SMI group. The difference may also be due to differences in the management or control of multimorbidity between the SMI and the non-SMI groups (e.g., better access to care services during the COVID-19 lockdown).

Given the observation that in the general population, COVID-19 has been associated with an excess risk of death in racially minoritized populations in the UK^9^, US^10–12^ and elsewhere^18 19^, we sought to explicitly assess whether deaths in these groups was more evident in people living with severe mental illnesses. Our assessment of race/ ethnicity indicated that Black Caribbean/ Black African patients had a higher risk of death following SARS-COV-2 infection, relative to the White reference group. We observed these ethnic inequalities to a similar extent across the SMI and the non-SMI control groups. This is consistent with previous work, and may suggest that the effect of living with a severe mental illnesses has a profound ‘ceiling’ effect leading to marked inequalities in mortality and physical health, which are then experienced to a similar extent across all racially minoritized groups^3^.

From a public health perspective, our study has emphasised the need for early and timely preventative interventions (e.g., vaccination) of the SMI population. Future studies are needed to disentangle the complex biological, psychosocial factors, and healthcare pathways that have led to the greater mortality rates in the SMI population^20 21^. For instance, we have recently documented that the presence of frailty increased fourfold the risk of all-cause mortality in people with SMI^22^. A recent expert prioritisation exercise highlighted a range of risk factors contributing to excess mortality in people with SMI. This included the challenges which people with SMI experience when accessing care that may be fragmented or of poorer quality, the impacts of stigmatising processes such as diagnostic overshadowing, and the role of socioeconomic disadvantage. Much routine care was withdrawn during the first and second waves of the pandemic, and face–to-face health care provision changed to remote/ telecare, which may have further impacted the quality of care received during the pandemic by people with SMI. Multimorbidity, including the presence of alcohol and substance use disorders, also likely affected the severity of the SMI disease and the risk of COVID-19 infections, as well as mortality outcomes. Multimorbidity also likely affected the severity of the SMI disease and the risk of COVID-19 infections, as well as mortality outcomes. Co-existing diseases may gave exacerbated inflammatory responses in the SMI population^23^ or may have reduced the functioning of specific body organs (e.g., lung, kidney). To prevent similar inequalities in future, it is essential to both improve the SMI population health and better integration of different clinical services for the SMI population during similar outbreaks.^24^

## Strengths and limitations

Although we were able to undertake a comprehensive assessment of COVID-19 infection in the cohort through using linked data sources, including information from national testing and surveillance systems, a limitation is that the presence of COVID-19 infection may have been under-diagnosed or under-reported in the records. This would have led to observed associations being estimated closer to the null. However, despite this, the impact of COVID-19 on mortality risk was substantially elevated across the sample. It is possible that there were some undiagnosed SMI cases in the control sample, however given previous work^25^ which has indicated that the recording of SMI in primary care records is either slightly higher or similar to community epidemiological samples, it is likely that this would have had a minimal impact on observed associations. A further limitation was that we did not have information on cause of death limiting our ability to assess cause-specific differences in mortality. Previous work has suggested that people living with SMI have experienced elevated risks of death from both COVID-19 and other causes over the course of the pandemic^5^. Despite being a relatively large sample, our assessment of ethnic inequalities may have been hampered by smaller sample sizes for the SMI group and incomplete data. While our analyses adjusted for a wide range of long-term conditions known to be associated with mortality, we cannot exclude the possibility of residual confounding. Sensitivity analyses for unmeasured confounding, however, confirm that this bias had a limited impact our findings. For example, the E-value for HR was 2.02 and 1.84 for the upper confidence interval, indicating that the evidence for causal association was reasonably strong even under the assumption that the study confounding control was relatively poor (the HR for study confounders were <1.3, with the exception of ICU). Also, we cannot exclude the possibility of reverse causation between COVID-19 infection and SMI. This limitation may have under- or over-estimated the true association between SMI with all-cause mortality in our population. To minimize the impact of reverse causation we have excluded all SMI diagnoses after a COVID-19 infection. Our study did not account for multiple COVID-19 infections, which may have affected the results pending unequal distribution in this rate between the SMI and non-SMI groups. Since our study timeline covered only the first two waves, this issue may be of lesser concern for the present findings. Furthermore, we had to group together some of the race/ ethnicity categories due to smaller sample sizes, and this would have impacted our analyses by potentially masking differences within groups. Further, we have not considered the potential mediating effect of the vaccination roll-out programme on the study outcome. The vaccination roll-out programme in the UK started around February/March 2021, and may have only minimally impacted our study results.

Strengths of the study include the prospective cohort design using a sample representative of the UK population by age, sex and ethnicity^26^, thus suggesting good generalisability and external validity. This is because most of the general population in the UK (98%) is under primary care and healthcare remains free at point of contact. In addition, the use of linked data would have meant that key exposures (COVID-19 tests/ diagnoses) were captured accurately in the health records^26^, albeit with the caveats mentioned above. UK primary care electronic health records have been noted as having high levels of recording with good accuracy for clinical diagnoses^27^, particularly since the introduction of financial incentives to manage chronic diseases^26^. It is possible that people living with SMI may have been less likely to be in contact with primary care, however previous studies have suggested that the incidence of SMI in UK primary care samples are comparable to the incidence reported in community samples^28^, suggesting that the issue of selection biases are less of a concern.

In conclusion, the findings from this study indicate that people living with severe mental illnesses have experienced large and substantial inequalities in mortality outcomes during the COVID-19 pandemic, which are further magnified through the presence of underlying health conditions and have been experienced to a similar extent across racialised populations. These inequalities were observed across multiple waves of the pandemic, which will likely exacerbate the life expectancy gap between people with SMI and the general population.

## Supplementary Material

Supplementary Material

## Figures and Tables

**Table 1:** Baseline demographic and clinical characteristics for the sample with positive COVID-19 test results, by Severe Mental Illness (SMI) status

	No SMI group	SMI group
**Sample size**	**653,024**	**7,146**
	N (%)/ mean (SD)	N (%)/ mean (SD)
**Age,** mean (SD)	43(20)	55(19)
**Sex**		
Female	360,937(55)	4,088(57)
**Body Mass Index (BMI)**		
Optimal (17.5-24.9)	185,523(28)	1,851(26)
Underweight (<17.5)	24,781(4)	223(3)
Overweight (25-29.9)	169,662(26)	2,041(29)
Obese (30+)	151,753(23)	2,757(38)
Missing	121,295(19)	270(4)
**Smoker**		
Never	372,224(57)	3,272(46)
Former	71,833(11)	1,728(24)
Current	130,605(20)	2,100(29)
Missing	78,363(12)	42(1)
Area deprivation*		
Least deprived	108,702(17)	786(11)
Second	112,968(17)	1,075(15)
Third	116,685(18)	1,210(17)
Fourth	139,900(21)	1,575(22)
Most deprived	147,329(23)	2,077(29)
Missing	27,440(4)	423(6)
**Race/ Ethnicity**		
White British/ Irish/ White Other	160,611(25)	4,777(67)
Black Caribbean/ Black African/ Black Other	22,829(3)	415(6)
Indian, Pakistani, Bangladeshi	57,934(9)	690(10)
Mixed ethnicity	7,630(1)	122(2)
Other	235,657(36)	362(5)
Missing	168,363(26)	776(11)
**UK Regions**		
London	143,665(22)	1,688(24)
North West England	139,938(23)	1,634(23)
North East England	41,800(7)	458(6)
West Midlands	121,784(17)	1,233(17)
East Midlands	32,651(5)	319(4)
South-East England	117,544(18)	1,255(18)
South-West England	58,772(8)	465(7)
Northern Ireland	774(<1)	17(<1)
Missing	413(<1)	73(1)
**Multimorbidities/ Long term health conditions**		
Hypertension	99,156(15)	1,806(25)
Myocardial infarction	10,319(2)	190(3)
Heart disease	21,028(3)	248(3)
Ischemic Stroke	7,386(1)	492(7)
Diabetes	51,192(8)	1,336(19)
Cancer	20,947(3)	666(9)
Liver disease	17,283(3)	410(6)
Kidney disease	29,902(5)	931(13)
Chronic Obstructive Pulmonary Disease (COPD)	24,876(4)	677(9)
Asthma	112,018(17)	1,501 (21)
Autoimmune	39,717(6)	651(9)
Substance use	8850(1)	835(12)
Epilepsy	10,251(2)	607(9)
Depression	139,588(21)	3,783(53)
Anxiety	142,329(22)	3,017(42)
Eating disorders	11,144(2)	287(4)
Gastroesophageal reflux disorder	16,591(3)	462(6)
Dementia	15,768(2)	265(4)
Immunological drug therapy	22.035(34)	2,806(39)
Corticosteroid drugs	84,895(13)	1,074(15)
Admissions to intensive care unit	659(0.10)	26(0.34)

* Area deprivation according to the Index of Multiple Deprivation (IMD)

## Data Availability

The analytical codes used in this study is available online (https://github.com/alexdregan/CPRD). The clinical codes are available from the corresponding author on request. Access to data are available only once approval has been obtained through the individual constituent entities controlling access to the data. The primary care data can be requested via application to the Clinical Practice Research Datalink.
