# Severe mental illness, race/ethnicity, multimorbidity and mortality following COVID-19 infection: nationally representative cohort study – ADDENDUM

**DOI:** 10.1192/bjp.2023.165

**Published:** 2024-01

**Authors:** Jayati Das-Munshi, Ioannis Bakolis, Laia Bécares, Jacqueline Dyer, Matthew Hotopf, Josephine Ocloo, Robert Stewart, Ruth Stuart, Alex Dregan

**Keywords:** Mortality, COVID-19, severe mental illness, multimorbidity, ethnicity, addendum

The authors regret the omission of the acknowledgement statement in the above article.

This study is based in part on data from the Clinical Practice Research Datalink obtained under licence from the UK Medicines and Healthcare products Regulatory Agency. The data is provided by patients and collected by the NHS as part of their care and support. The interpretation and conclusions contained in this study are those of the author/s alone.
