# Supplementary material for: Severe mental illnesses, race/ ethnicity, multimorbidity and mortality following COVID-19 infection: Nationally representative cohort study
Source: Br J Psychiatry. Author manuscript; Available in PMC 2023 Nov 1. (PMC7615273; doi:10.1192/bjp.2023.112)
Supplement: Supplementary Material [file EMS184798-supplement-Supplementary_Material.pdf]

Title: Severe mental illnesses, multimorbidity and mortality following COVID-19 infection: Nationally representative cohort study

Authors: Jayati Das-Munshi PhD, Ioannis Bakolis PhD, Laia Bécares PhD, Jacqui Dyer PhD, Matthew Hotopf PhD, Josephine Ocloo PhD, Robert Stewart MD, Ruth Stuart MSc, Alex Dregan PhD

Supplementary material

Figures:5

Supplementary Figure 1: Hazard Ratios for the association of severe mental illnesses (SMI) with all-cause mortality, following COVID-19 infection. Complete case models

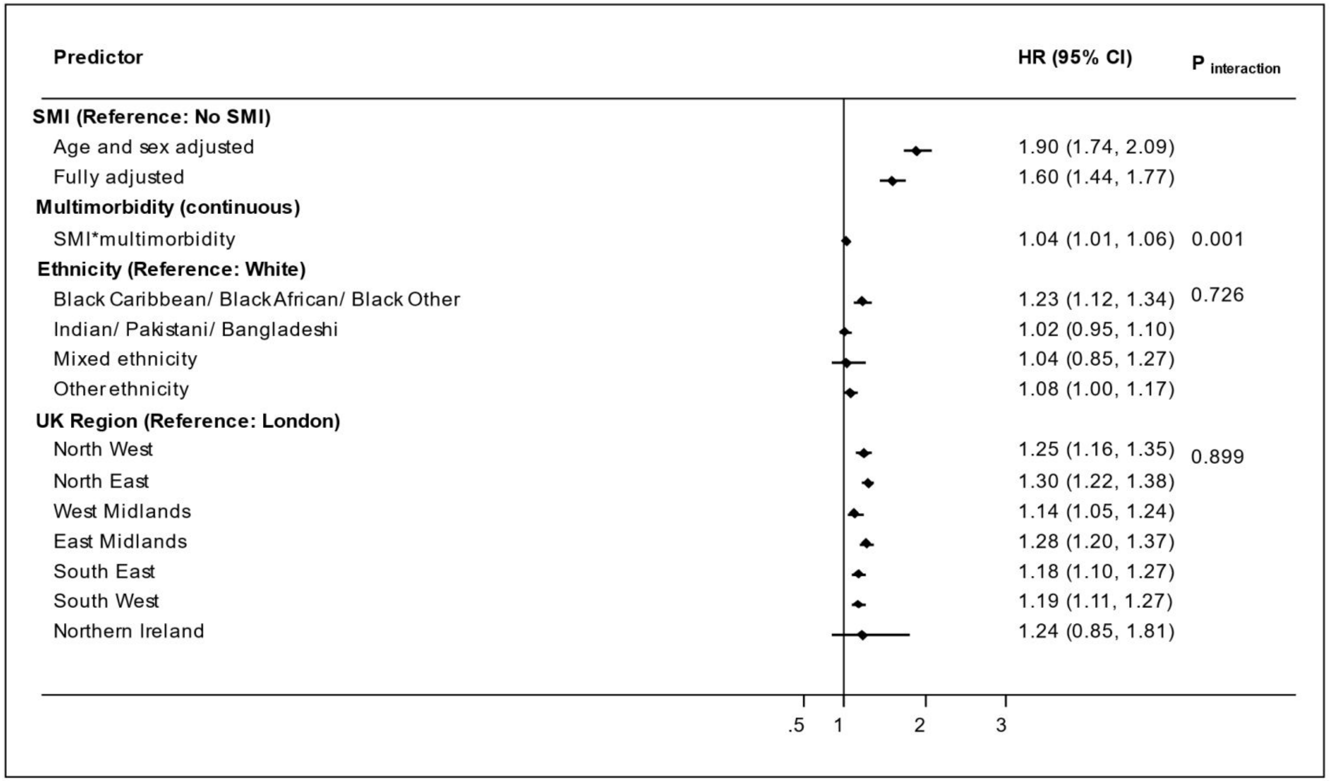

Key: Displayed estimates are from complete case models. SMI- severe mental illness. 'Fully adjusted' models adjusted for age, sex, race/ ethnicity, UK regions, deprivation, BMI, smoking status and all comorbidities displayed in Table 1 and an interaction term between SMI*multimorbidity.

Supplementary Figure 2: Covariates adjusted survival probability following COVID-19 infection by severe mental illness (SMI) status, over the first year of the COVID-19 pandemic

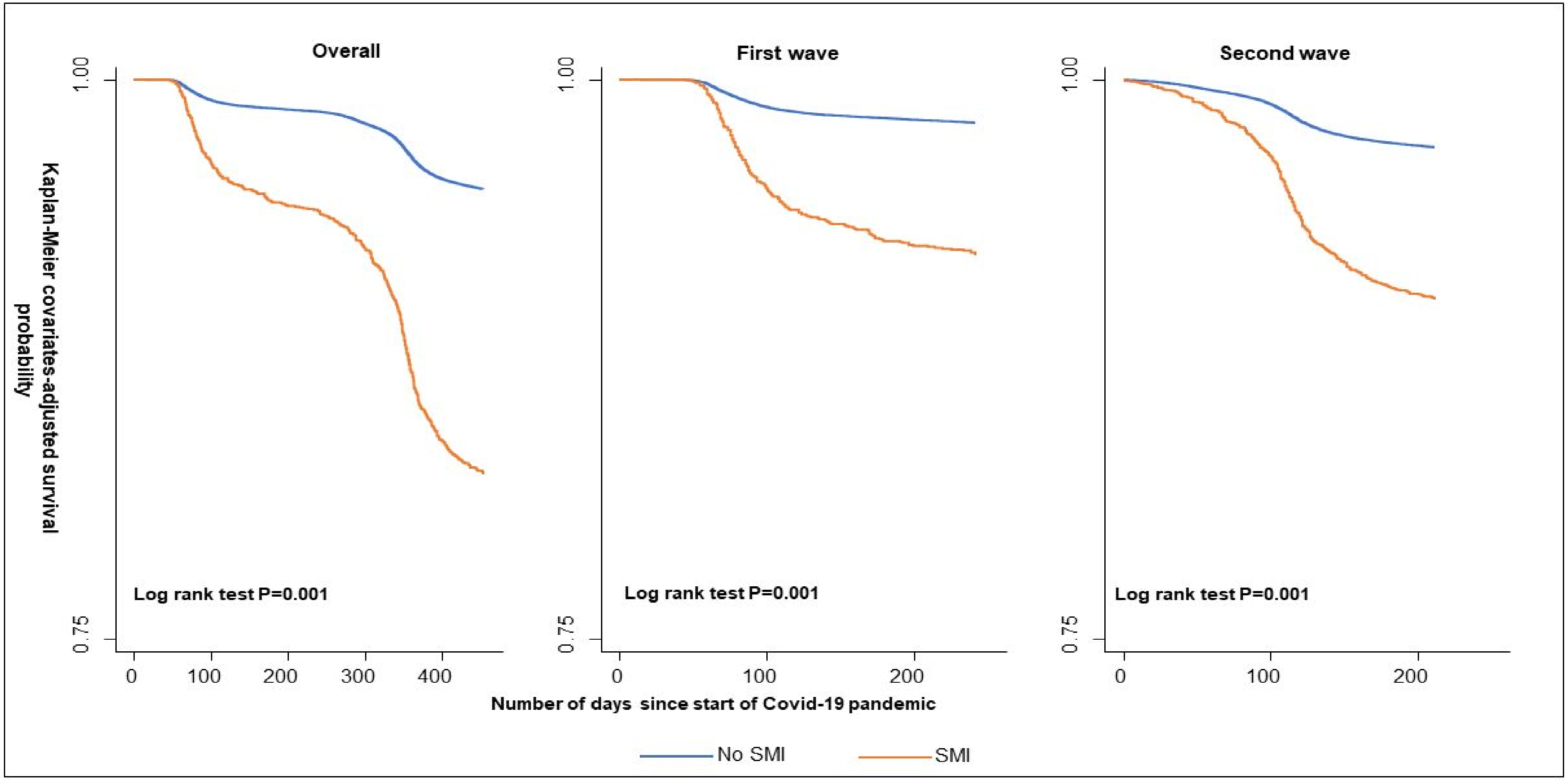

Key: SMI- severe mental illness. **Notes**: For ease of understanding we graphs used a cut-off point of 0.75.

Supplementary Figure 3: Hazard Ratios for the association of severe mental illnesses (SMI) with all-cause mortality, following COVID-19 infection in patients aged 18 years or over at the time of COVID-19 pandemic.

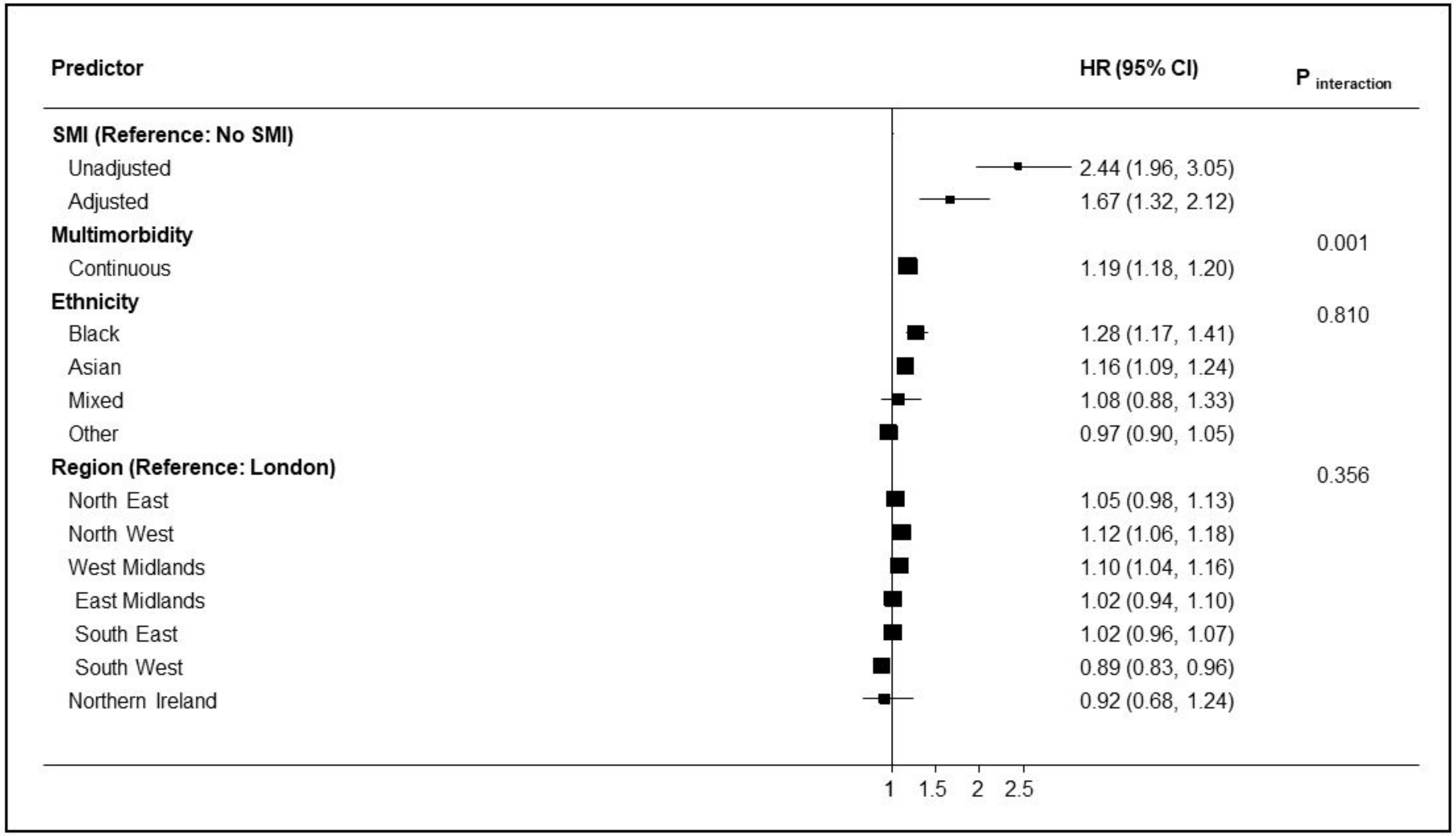

Key: Displayed estimates are from imputed estimates. SMI- severe mental illness. Fully adjusted' models adjusted for age, sex, multimorbidity, ethnicity. UK region and all comorbidities from Table 1. The HR include a combination of independent and interaction effects. The HR for SMI represents the effect of SMI on all-cause mortality in the absence of multimorbidity; the interaction effect represents the additional (multiplicative) effects of SMI in the presence of multimorbidity. Effect sizes for Ethnicity and UK Region variables represent the fully adjusted models analysis.

Table S1. Baseline demographic and clinical characteristics for the matched sample with positive COVID-19 test results, by Severe Mental Illness (SMI) status

	**No SMI group**	**SMI group**
**Sample size**	**35,575**	**7,146**
	N (%)/ mean (SD)	N (%)/ mean (SD)
**Age,** mean (SD)	55(19)	55(19)
**Sex**		
Female	20,381(57)	4,088(57)
**Body Mass Index (BMI)**		
Optimal (17.5-24.9)	9,982(28)	1,851(26)
Underweight (<17.5)	814(2)	223(3)
Overweight (25-29.9)	11,385(32)	2,041(29)
Obese (30+)	10,511(30)	2,757(38)
Missing	2,883(8)	270(4)
**Smoker**		
Never	20,248(57)	3,272(46)
Former	8,706(24)	1,728(24)
Current	6,011(17)	2,100(29)
Missing	610(2)	42(1)
**Area deprivation***		
Least deprived	5,364(15)	786(11)
Second	5,878(17)	1,075(15)
Third	6,311(18)	1,210(17)
Fourth	7,715(21)	1,575(22)
Most deprived	8,804(25)	2,077(29)
Missing	1,503(4)	423(6)
**Race/ Ethnicity**		
White British/ Irish/ White Other	23,423(66)	4,777(67)
Black Caribbean/ Black African/ Black Other	1,287(4)	415(6)
Indian, Pakistani, Bangladeshi	3,875(11)	690(11)
Mixed ethnicity	389(1)	122(2)
Other	1,638(5)	362(5)
Missing	4,963(14)	776(11)
**UK Regions**		
London	7,361(21)	1,688(24)
North West England	8,499(24)	1,634(23)

North East England	2,443(6)	458(6)
West Midlands	6,309(18)	1,233(17)
East Midlands	1,764(5)	319(4)
South-East England	6,340(18)	1,255(18)
South-West England	2,422(7)	465(7)
Northern Ireland	61(<1)	17(<1)
Missing	376(1)	73(1)
**Multimorbidities/ Long term health conditions**		
Hypertension	9,449(27)	1,806(25)
Myocardial infarction	1,033(3)	190(3)
Heart disease	1,314(4)	248(3)
Ischemic Stroke	1,686(5)	492(7)
Diabetes	4,409(12)	1,336(19)
Cancer	3,681(10)	666(9)
Liver disease	1,288(4)	410(6)
Kidney disease	3,513(10)	931(13)
Chronic Obstructive Pulmonary Disease (COPD)	2,509(7)	677(9)
Asthma	5,818(16)	1,501 (21)
Autoimmune	3,227(9)	651(9)
Substance use	540(2)	835(12)
Epilepsy	950(3)	607(9)
Depression	8,157(23)	3,783(53)
Anxiety	7,545(21)	3,017(42)
Eating disorders	387(1)	287(4)
Gastroesophageal reflux disorder	2,127(6)	462(6)
Dementia	865(2)	265(4)
Immunological drug therapy	14,728(41)	2,806(39)
Corticosteroid drugs	6,032(17)	1,074(15)
Admissions to intensive care unit	42(0.12)	26(0.34)

Supplementary Figure 4: Hazard Ratios for the association of severe mental illnesses (SMI) with all-cause mortality, following COVID-19 infection. Multimorbidity matched patients

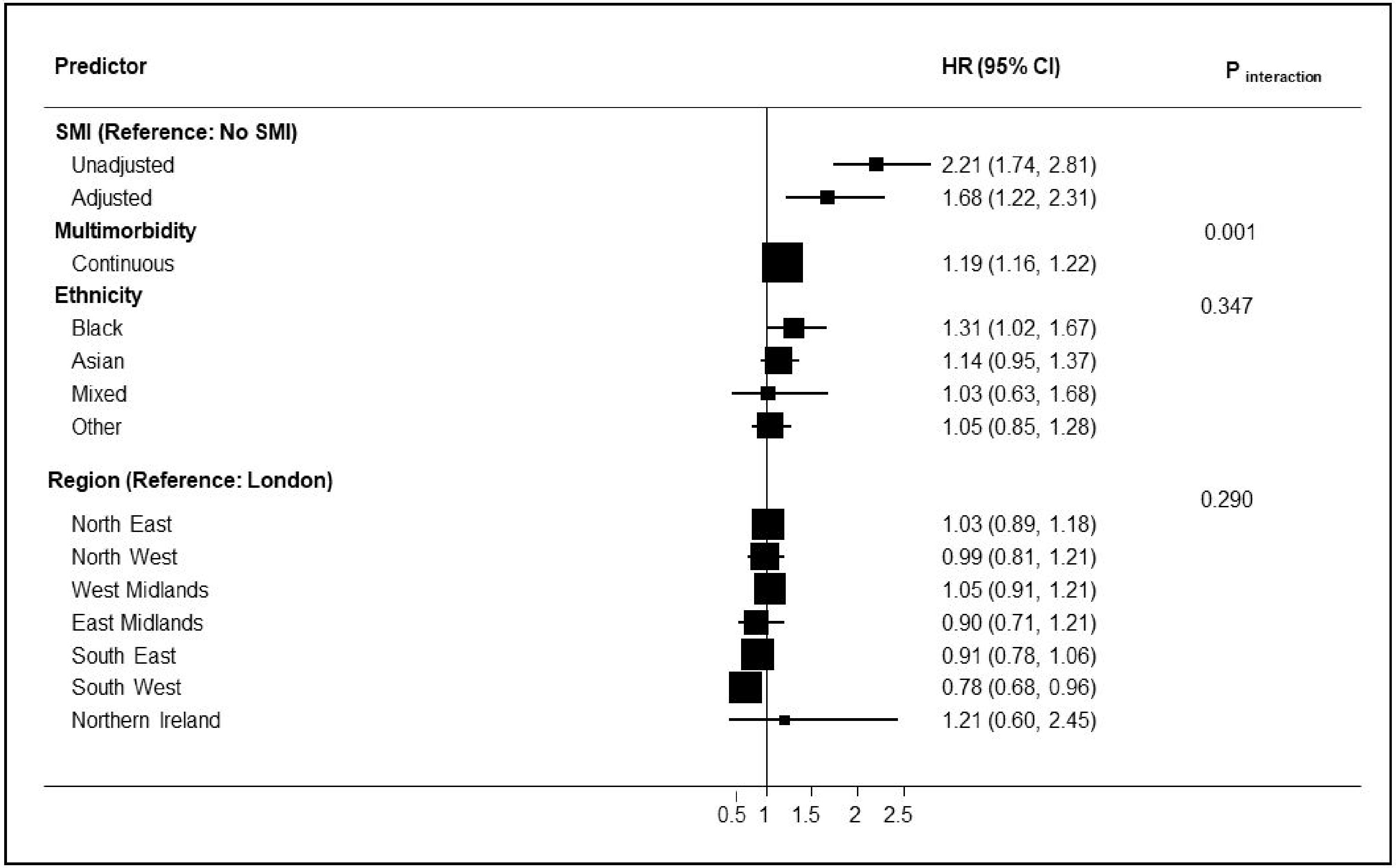

Key: Displayed estimates are from imputed estimates. SMI- severe mental illness. Fully adjusted' models adjusted for age, sex, multimorbidity, ethnicity. UK region and all comorbidities from Table 1. The HR include a combination of independent and interaction effects. The HR for SMI represents the effect of SMI on all-cause mortality in the absence of multimorbidity; the interaction effect represents the additional (multiplicative) effects of SMI in the presence of multimorbidity. Effect sizes for Ethnicity and UK Region variables represent the fully adjusted models analysis.

SMI medical codes

Read code	Snomed concept id	Medical term
E1005	4926007	Schizophrenia in remission
E13y1	5464005	Brief reactive psychosis
E1001	16990005	Subchronic schizophrenia
E106	26025008	Residual schizophrenia
E1032	31658008	Chronic paranoid schizophrenia
E1021	42868002	Subchronic catatonic schizophrenia
E1035	63181006	Paranoid schizophrenia in remission
E103	64905009	Paranoid schizophrenia
E1022	68995007	Chronic catatonic schizophrenia
E141	71961003	Disintegrative psychosis
E1031	79866005	Subchronic paranoid schizophrenia
1464	161468000	H/O: schizophrenia
E0111	191471000	Korsakov's alcoholic psychosis with peripheral neuritis
E01y	42344001	Other alcoholic psychosis
E01yz	42344001	Other alcoholic psychosis NOS
E01z	42344001	Alcoholic psychosis NOS
E02z	191483003	Drug psychosis NOS
E04z	191447007	Chronic organic psychosis NOS
E10	191526005	Schizophrenic disorders
E100-1	191527001	Schizophrenia simplex
E100	191527001	Simple schizophrenia
E1000	58214004	Unspecified schizophrenia
E1003	111482003	Acute exacerbation of subchronic schizophrenia
E1004	191531007	Acute exacerbation of chronic schizophrenia
E100z	191527001	Simple schizophrenia NOS

E1010	35252006	Unspecified hebephrenic schizophrenia
E1011	27387000	Subchronic hebephrenic schizophrenia
E1012	12939007	Chronic hebephrenic schizophrenia
E1013	14291003	Acute exacerbation of subchronic hebephrenic schizophrenia
E1014	191539009	Acute exacerbation of chronic hebephrenic schizophrenia
E101z	35252006	Hebephrenic schizophrenia NOS
E102	191542003	Catatonic schizophrenia
Eu202-4	191542003	[X]Schizophrenic flexibilatis cerea
E1020	191542003	Unspecified catatonic schizophrenia
E1023	191547009	Acute exacerbation of subchronic catatonic schizophrenia
E1024	191548004	Acute exacerbation of chronic catatonic schizophrenia
E102z	191542003	Catatonic schizophrenia NOS
E1030	64905009	Unspecified paranoid schizophrenia
E1033	191554003	Acute exacerbation of subchronic paranoid schizophrenia
E1034	191555002	Acute exacerbation of chronic paranoid schizophrenia
E103z	64905009	Paranoid schizophrenia NOS
E105	191559008	Latent schizophrenia
E1050	191559008	Unspecified latent schizophrenia
E1051	191561004	Subchronic latent schizophrenia
E1052	191562006	Chronic latent schizophrenia
E1053	191563001	Acute exacerbation of subchronic latent schizophrenia
E1054	191564007	Acute exacerbation of chronic latent schizophrenia
E105z	191559008	Latent schizophrenia NOS
E107-1	191567000	Cyclic schizophrenia
E10y1	191577003	Coenesthopathic schizophrenia
E10y-1	191577003	Cenesthopathic schizophrenia
E10yz	58214004	Other schizophrenia NOS
E10z	58214004	Schizophrenia

E1100	268619003	Single manic episode, unspecified
E1101	191583000	Single manic episode, mild
E1102	191584006	Single manic episode, moderate
E1103	764641000000104	Single manic episode, severe
E1104	191586008	Single manic episode, severe, with psychosis
E1105	764731000000103	Single manic episode in partial remission
E110z	268619003	Manic disorder, single episode NOS
E111	191590005	Recurrent manic episodes
E1110	191590005	Recurrent manic episodes, unspecified
E1111	191592002	Recurrent manic episodes, mild
E1112	191593007	Recurrent manic episodes, moderate
E1114	191595000	Recurrent manic episodes, severe, with psychosis
E111z	191590005	Recurrent manic episode NOS
E114-1	191618007	Manic-depressive - now manic
E1140	191618007	Bipolar affective disorder, currently manic, unspecified
E1141	191620005	Bipolar affective disorder, currently manic, mild
E1142	191621009	Bipolar affective disorder, currently manic, moderate
E114z	191618007	Bipolar affective disorder, currently manic, NOS
E115-1	191627008	Manic-depressive - now depressed
E1150	191627008	Bipolar affective disorder, currently depressed, unspecified
E1151	191629006	Bipolar affective disorder, currently depressed, mild
E1152	191630001	Bipolar affective disorder, currently depressed, moderate
E115z	191627008	Bipolar affective disorder, currently depressed, NOS
E116	191636007	Mixed bipolar affective disorder
E1160	191636007	Mixed bipolar affective disorder, unspecified
E1161	191638008	Mixed bipolar affective disorder, mild
E1162	191639000	Mixed bipolar affective disorder, moderate
E1164	191641004	Mixed bipolar affective disorder, severe, with psychosis

E116z	191636007	Mixed bipolar affective disorder, NOS
E117	13746004	Unspecified bipolar affective disorder
E1170	13746004	Unspecified bipolar affective disorder, unspecified
E1171	13313007	Mild bipolar disorder
E1172	79584002	Moderate bipolar disorder
E117z	13746004	Unspecified bipolar affective disorder, NOS
E11y	13746004	Other and unspecified manic-depressive psychoses
E11y0	13746004	Unspecified manic-depressive psychoses
E11y1	191658009	Atypical manic disorder
E11y3	16506000	Mixed bipolar I disorder
E11yz	13746004	Other and unspecified manic-depressive psychoses NOS
E11z	441704009	Affective psychosis
E11zz	441704009	Other affective psychosis NOS
E12z	191667009	Paranoid psychosis
E130	191676002	Reactive depressive psychosis
E131	191677006	Acute hysterical psychosis
E134	191680007	Psychogenic paranoid psychosis
E141z	71961003	Disintegrative psychosis NOS
Eu2	417601000000102	[X]Schizophrenia, schizotypal and delusional disorders
Eu203	111484002	Undifferentiated schizophrenia
Eu20z	58214004	[X]Schizophrenia, unspecified
Eu22z	231487004	[X]Persistent delusional disorder, unspecified
Eu23y	231489001	[X]Other acute and transient psychotic disorders
Eu25y	68890003	[X]Other schizoaffective disorders
Eu25z	68890003	[X]Schizoaffective disorder, unspecified
Eu301	231494001	Mania
Eu30y	231494001	[X]Other manic episodes
Eu31z	13746004	[X]Bipolar affective disorder, unspecified
E13z-1	69322001	Psychotic disorder

E00y-1	231438001	Presbyophrenic psychosis
E14z-1	191687005	Childhood schizophrenia NOS
E104	268617001	Acute schizophrenic episode
E10y	58214004	Other schizophrenia
E110	268619003	Manic disorder, single episode
E121	268622001	Chronic paranoid psychosis
E1z	191525009	Non-organic psychosis NOS
E14z	191687005	Child psychosis NOS
Eu20y	58214004	[X]Other schizophrenia
Eu21	31027006	Schizotypal personality disorder
Eu22y	231487004	[X]Other persistent delusional disorders
Eu233	231489001	[X]Other acute predominantly delusional psychotic disorders
Eu23z	231489001	[X]Acute and transient psychotic disorder, unspecified
Eu2z	191525009	[X]Unspecified nonorganic psychosis
Eu302	231494001	[X]Mania with psychotic symptoms
Eu30z	268619003	[X]Manic episode, unspecified
Eu31y	13746004	[X]Other bipolar affective disorders
285-1	268957000	Psychotic condition, insight present
ZV110	161464003	[V]Personal history of schizophrenia
E10y0	111484002	Atypical schizophrenia
E1002	83746006	Chronic schizophrenic
E1070	191567000	Unspecified schizo-affective schizophrenia
E1175	5703000	Bipolar disorder in partial remission
E1173	53049002	Severe bipolar disorder without psychotic features
E1174	4441000	Severe bipolar disorder with psychotic features
E1071	191569002	Subchronic schizo-affective schizophrenia
E107	191567000	Schizoaffective schizophrenia
E1075	191574005	Schizoaffective schizophrenia in remission

E107z	191567000	Schizo-affective schizophrenia NOS
E2122	31027006	Schizotypal personality
E106-1	26025008	Restzustand - schizophrenia
E1115	764671000000105	Recurrent manic episodes, in partial remission
E1113	764621000000106	Recurrent manic episodes, severe
Eu220	48500005	Delusional disorder
Eu220-1	191667009	[X]Paranoid psychosis
E11-3	231494001	Manic psychosis
E13z	191525009	Non-organic psychosis
8HHs	199191000000109	Referral to psychosis early intervention service
Eu02z-2	268612007	[X] Presenile psychosis NOS
Eu02z-5	268612007	[X] Senile psychosis NOS
Eu04-3	191502008	[X]Acute / subacute infective psychosis
Eu23	231489001	Acute transient psychotic disorder
Eu231	712850003	Acute polymorphic psychotic disorder co-occurrent with symptoms of schizophrenia
Eu230	712824002	Acute polymorphic psychotic disorder without symptoms of schizophrenia
Eu232	278853003	Acute schizophrenia-like psychotic disorder
Eu3z-1	46206005	[X]Affective psychosis NOS
Eu105-4	42344001	[X]Alcoholic psychosis NOS
Eu841-1	231536004	[X]Atypical childhood psychosis
Eu203-1	111484002	[X]Atypical schizophrenia
Eu314	61403008	Severe depressed bipolar I disorder without psychotic features
Eu312	191623007	Bipolar affective disorder, currently manic, severe, with psychosis
Eu311	191618007	[X]Bipolar affect disorder cur epi manic wout psychotic symp

Eu313	191630001	[X]Bipolar affect disorder cur epi mild or moderate depressn
Eu31	13746004	Bipolar affective disorder
Eu310	31446002	Bipolar affective disorder, current episode hypomanic
Eu316	192362008	[X]Bipolar affective disorder, current episode mixed
Eu30-1	268619003	[X]Bipolar disorder, single manic episode
Eu31y-1	83225003	[X]Bipolar II disorder
Eu21-2	274952002	Borderline schizophrenia
Eu231-1	712850003	[X]Bouffee delirante with symptoms of schizophrenia
Eu23z-1	231489001	[X]Brief reactive psychosis NOS
Eu232-1	278853003	[X]Brief schizophreniform disorder
Eu232-2	278853003	[X]Brief schizophrenifrm psych
Eu202	191542003	[X]Catatonic schizophrenia
Eu202-1	191542003	[X]Catatonic stupor
Eu20y-1	58214004	[X]Cenesthopathic schizophrenia
Eu2y-1	480111000000107	[X]Chronic hallucinatory psychosis
Eu205-1	111484002	[X]Chronic undifferentiated schizophrenia
Eu252-1	270901009	[X]Cyclic schizophrenia
Eu230-2	307417003	[X]Cycloid psychosis
Eu231-2	307417003	Cycloid psychosis
Eu340	76105009	Cyclothymia
Eu22y-1	231487004	[X]Delusional dysmorphophobia
Eu843-2	35919005	[X]Disintegrative psychosis
Eu201-1	35252006	[X]Disorganised schizophrenia
Eu05y-1	111479008	[X]Epileptic psychosis NOS
Eu201	35252006	[X]Hebephrenic schizophrenia
Eu300	231496004	Hypomania
Eu44-4	44376007	[X]Hysterical psychosis
Eu24-2	61831009	[X]Induced paranoid disorder

Eu24-3	61831009	[X]Induced psychotic disorder
Eu840-3	408858002	[X]Infantile psychosis
Eu22y-2	231487004	[X]Involutional paranoid state
Eu106-1	69482004	Korsakoff's psychosis
Eu03-1	3298001	[X]Korsakov's psychosis, nonalcoholic
Eu21-3	191559008	[X]Latent schizophrenia
Eu21-1	191559008	[X]Latent schizophrenic reaction
Eu31-3	13746004	[X]Manic-depressive reaction
Eu30z-1	268619003	[X]Mania NOS
Eu302-1	231494001	[X]Mania with mood-congruent psychotic symptoms
Eu302-2	231494001	[X]Mania with mood-incongruent psychotic symptoms
Eu30	268619003	[X]Manic episode
Eu302-3	231494001	[X]Manic stupor
Eu332-3	36474008	[X] Manic-depressive psychosis, depressed type without psychotic symptoms
Eu333-2	765176007	[X]Manic-depress psychosis,depressed type+psychotic symptoms
Eu31-1	13746004	Manic-depressive illness
Eu31-2	13746004	Manic-depressive psychosis
Eu125	943081000000102	Cannabis-induced psychosis
Eu105	42344001	Alcohol-induced psychosis
Eu145	943101000000108	Cocaine-induced psychosis
Eu115	943071000000104	Opioid-induced psychosis
Eu3y0-1	192362008	Bipolar affective disorder , current episode mixed
Eu252-2	270901009	Mixed schizophrenic and affective pschosis
Eu232-3	278853003	[X]Oneirophrenia
Eu052	5510009	Organic delusional disorder
Eu0z-1	111479008	[X]Organic psychosis NOS
Eu2y	191525009	[X]Other nonorganic psychotic disorders

Eu22y-3	231487004	[X]Paranoia querulans
Eu200	64905009	[X]Paranoid schizophrenia
Eu200-1	64905009	[X]Paraphrenic schizophrenia
Eu22	231487004	Persistent delusional disorder
Eu21-4	247804008	Prepsychotic schizophrenia
Eu21-5	247804008	Prodromal schizophrenia
Eu21-6	31027006	[X]Pseudoneurotic schizophrenia
Eu21-7	31027006	[X]Pseudopsychopathic schizophrenia
Eu233-2	191680007	[X]Psychogenic paranoid psychosis
Eu2z-1	69322001	Psychotic
Eu531-1	199260001	[X]Puerperal psychosis NOS
Eu23z-2	231437006	[X]Reactive psychosis
Eu333-4	191613003	[X]Recurr severe episodes/psychogenic depressive psychosis
Eu31y-2	191590005	[X]Recurrent manic episodes
Eu205	26025008	[X]Residual schizophrenia
Eu205-2	26025008	[X]Restzustand schizophrenic
Eu251	84760002	Schizoaffective disorder, depressive type
Eu250	271428004	Schizoaffective disorder, manic type
Eu252	270901009	Schizoaffective disorder, mixed type
Eu25	68890003	Schizoaffective disorder
Eu25z-1	68890003	[X]Schizoaffective psychosis NOS
Eu251-1	84760002	[X]Schizoaffective psychosis, depressive type
Eu250-1	271428004	[X]Schizoaffective psychosis, manic type
Eu20	58214004	[X]Schizophrenia
Eu052-2	5510009	[X]Schizophrenia-like psychosis in epilepsy
Eu202-2	191542003	[X]Schizophrenic catalepsy
Eu202-3	191542003	[X]Schizophrenic catatonia
Eu232-4	278853003	[X]Schizophrenic reaction

Eu20y-2	58214004	[X]Schizophreniform disord NOS
Eu251-2	84760002	[X]Schizophreniform psychosis, depressive type
Eu250-2	271428004	[X]Schizophreniform psychosis, manic type
Eu20y-3	58214004	[X]Schizophrenifrm psychos NOS
Eu21-8	31027006	[X]Schizotypal personality disorder
Eu206	191527001	[X]Simple schizophrenia
Eu843-4	35919005	[X]Symbiotic psychosis
E1074	191572009	Acute exacerbation of chronic schizo-affective schizophrenia
E1073	191571002	Acute exacerbation subchronic schizo-affective schizophrenia
E01	42344001	Alcohol-induced psychosis
E1143	162004	Severe manic bipolar I disorder without psychotic features
E1144	191623007	Bipolar affect disord, currently manic,severe with psychosis
E1155	49512000	Depressed bipolar I disorder in partial remission
E1154	765176007	Bipolar affect disord, now depressed, severe with psychosis
E1153	61403008	Bipolar affect disord, now depressed, severe, no psychosis
E1145	63249007	Manic bipolar I disorder in partial remission
E115	191627008	Bipolar affective disorder, current episode depression
E114	191618007	Bipolar affective disorder, current episode manic
E11-1	13746004	Bipolar disorder
E1072	191570001	Chronic schizoaffective schizophrenia
E02	191483003	Drug psychosis
E1165	760721000000109	Mixed bipolar affective disorder, partial/unspec remission
E1163	764591000000108	Mixed bipolar affective disorder, severe
E0110	69482004	Korsakov alcoholic psychosis

E110-1	231496004	Hypomanic psychoses
E101	35252006	Hebephrenic schizophrenia
EMISQHY1	851691000006105	Hypomanic
EGTON118	853201000006100	Obsessional compulsive psychosis
E03y3-99	551591000000100	Puerperal psychosis
E10-98	191526005	Schizophrenic psychoses NOS
E10-99	191526005	Schizophrenic psychoses
E107-99	191567000	Acute schizo affective psychosis
E11-99	13746004	Manic-depressive psychoses
E110-99	268619003	Mania/hypomania
212T	200951000000109	Psychosis, schizophrenia and bipolar affective disorder resolved
Eu318	371596008	Bipolar I disorder
Eu319	83225003	Bipolar II disorder
Eu319-1	83225003	[X]Bipolar II disorder
EMISICD10|F0630	58329000	Organic manic disorder
EMISICD10|F0631	197210100006110	Organic bipolar affective disorder
EMISICD10|F2000	1974351000006100	Paranoid schizophrenia, continuous
EMISICD10|F2001	1974391000006110	Paranoid schizophrenia, episodic with progressive deficit
EMISICD10|F2002	1974431000006100	Paranoid schizophrenia, episodic with stable deficit
EMISICD10|F2003	1974451000006110	Paranoid schizophrenia, episodic remittent
EMISICD10|F2004	1974481000006100	Paranoid schizophrenia, incomplete remission
EMISICD10|F2009	1974541000006110	Paranoid schizophrenia, course uncertain, period of observation too short
EMISICD10|F2010	1974601000006110	Hebephrenic schizophrenia, continuous
EMISICD10|F2011	1974611000006100	Hebephrenic schizophrenia, episodic with progressive deficit
EMISICD10|F2012	1974621000006100	Hebephrenic schizophrenia, episodic with stable deficit
EMISICD10|F2013	1974641000006110	Hebephrenic schizophrenia, episodic remittent

EMISICD10|F2014	1974671000006100	Hebephrenic schizophrenia, incomplete remission
EMISICD10|F2019	1974721000006100	Hebephrenic schizophrenia, course uncertain, period of observation too short
EMISICD10|F2020	1974751000006100	Catatonic schizophrenia, continuous
EMISICD10|F2021	1974781000006110	Catatonic schizophrenia, episodic with progressive deficit
EMISICD10|F2022	1974801000006110	Catatonic schizophrenia, episodic with stable deficit
EMISICD10|F2023	1974831000006100	Catatonic schizophrenia, episodic remittent
EMISICD10|F2024	1974861000006110	Catatonic schizophrenia, incomplete remission
EMISICD10|F2029	1974921000006110	Catatonic schizophrenia, course uncertain, period of observation too short
EMISICD10|F2034	1975051000006100	Undifferentiated schizophrenia, incomplete remission
EMISICD10|F2050	1975351000006100	Residual schizophrenia, continuous
EMISICD10|F2051	1975381000006110	Residual schizophrenia, episodic with progressive deficit
EMISICD10|F2052	1975401000006110	Residual schizophrenia, episodic with stable deficit
EMISICD10|F2053	1975431000006100	Residual schizophrenia, episodic remittent
EMISICD10|F2054	1975441000006110	Residual schizophrenia, incomplete remission
EMISICD10|F2059	1975481000006100	Residual schizophrenia, course uncertain, period of observation too short
EMISICD10|F2060	1975491000006100	Simple schizophrenia, continuous
EMISICD10|F2061	1975501000006100	Simple schizophrenia, episodic with progressive deficit
EMISICD10|F2062	1975521000006100	Simple schizophrenia, episodic with stable deficit
EMISICD10|F2063	1975551000006110	Simple schizophrenia, episodic remittent
EMISICD10|F2064	1975571000006100	Simple schizophrenia, incomplete remission
EMISICD10|F2300	1975621000006100	Acute polymorphic psychot disord without symp of schizoph, without associated acute stress
EMISICD10|F2069	1975641000006110	Simple schizophrenia, course uncertain, period of observation too short
EMISICD10|F2301	1975671000006100	Acute polymorphic psychot disord without symp of schizoph, with associated acute stress

EMISICD10|F2310	1975681000006100	Acute polymorphic psychot disord with symp of schizophren, without associated acute stress
EMISICD10|F2311	1975691000006100	Acute polymorphic psychot disord with symp of schizophren, with associated acute stress
EMISICD10|F2320	1975711000006100	Acute schizophrenia-like psychotic disorder, without associated acute stress
EMISICD10|F2321	1975731000006110	Acute schizophrenia-like psychotic disorder, with associated acute stress
EMISICD10|F2080	1975751000006100	Other schizophrenia, continuous
EMISICD10|F2330	1975761000006100	Other acute predominantly delusional psychotic disorders, without associated acute stress
EMISICD10|F2081	1975771000006100	Other schizophrenia, episodic with progressive deficit
EMISICD10|F2331	1975781000006110	Other acute predominantly delusional psychotic disorders, with associated acute stress
EMISICD10|F2082	1975801000006110	Other schizophrenia, episodic with stable deficit
EMISICD10|F2380	1975821000006100	Other acute and transient psychotic disorders, without associated acute stress
EMISICD10|F2083	1975831000006100	Other schizophrenia, episodic remittent
EMISICD10|F2084	1975841000006110	Other schizophrenia, incomplete remission
EMISICD10|F2381	1975851000006110	Other acute and transient psychotic disorders, with associated acute stress
EMISICD10|F2390	1975871000006100	Acute and transient psychotic disorder, unspecified, without associated acute stress
EMISICD10|F2391	1975901000006100	Acute and transient psychotic disorder, unspecified, with associated acute stress
EMISICD10|F2089	1975911000006100	Other schizophrenia, course uncertain, period of observation too short
EMISICD10|F3130	1975931000006110	Bipolar affect disorder cur epi mild or moderate depressn, without somatic syndrome
EMISICD10|F3131	1975941000006100	Bipolar affect disorder cur epi mild or moderate depressn, with somatic syndrome

EMISICD10|F2090	1976001000006110	Schizophrenia, unspecified, continuous
EMISICD10|F2091	1976061000006100	Schizophrenia, unspecified, episodic with progressive deficit
EMISICD10|F2092	1976101000006110	Schizophrenia, unspecified, episodic with stable deficit
EMISICD10|F2093	1976121000006100	Schizophrenia, unspecified, episodic remittent
EMISICD10|F2094	1976141000006100	Schizophrenia, unspecified, incomplete remission
EMISICD10|F2099	1976171000006100	Schizophrenia, unspecified, course uncertain, period of observation too short
EMISICD10|F2008	1976881000006110	Paranoid schizophrenia, other
EMISICD10|F2018	1976891000006110	Hebephrenic schizophrenia, other
EMISICD10|F2028	1976901000006100	Catatonic schizophrenia, other
EMISICD10|F2038	1976911000006110	Undifferentiated schizophrenia, other
EMISICD10|F2058	1976931000006100	Residual schizophrenia, other
EMISICD10|F2068	1976941000006110	Simple schizophrenia, other
EMISICD10|F2088	1976951000006110	Other schizophrenia, other
EMISICD10|F2098	1976961000006110	Schizophrenia, unspecified, other
^ESCTBR258680	5464005	Brief psychotic disorder
^ESCT1171785	1089671000000110	Mania with mood-incongruent psychotic features
^ESCT1171786	1089681000000110	Mania with psychotic features
^ESCT1171787	1089691000000100	Acute predominantly delusional psychotic disorder
^ESCTSC300026	31027006	Schizoptypal disorder
^ESCTBI300667	31446002	Bipolar I disorder, most recent episode hypomanic
^ESCTDI306812	35252006	Disorganised schizophrenia
^ESCTSC311804	38368003	Schizoaffective disorder, bipolar type
^ESCTUN313726	39610001	Undifferentiated schizophrenia in remission
^ESCTOR344778	58329000	Organic mood disorder of manic type
^ESCTSE346839	59617007	Severe bipolar I disorder, most recent episode depressed with psychotic features
^ESCTIN350472	61831009	Induced psychotic disorder

^ESCTIN350475	61831009	Induced psychosis
^ESCTIN350479	61831009	Induced paranoid disorder
^ESCTPS362734	69322001	Psychosis
^ESCTKO363012	69482004	Korsakoff psychosis
^ESCTKO363015	69482004	Korsakov psychosis
^ESCTCY373801	76105009	Cyclothymic disorder
^ESCTBI385440	83225003	Bipolar 2 disorder
^ESCTCH386266	83746006	Chronic schizophrenia
^ESCTSC394614	88975006	Schizophreniform disorder
^ESCTSC408293	102940002	Schizophrenic reaction
^ESCTHI453993	161468000	History of schizophrenia
^ESCTDR476321	191483003	Drug-induced psychosis
^ESCTAC476364	191539009	Acute exacerbation of chronic disorganised schizophrenia
^ESCTAC476391	191571002	Acute exacerbation of subchronic schizoaffective schizophrenia
^ESCTAC476392	191572009	Acute exacerbation of chronic schizoaffective schizophrenia
^ESCTPS476451	191687005	Psychosis with origin in childhood
^ESCTBI476731	192362008	Bipolar affective disorder, current episode mixed
^ESCTOR502345	231444002	Organic bipolar disorder
^ESCTEP502353	231449007	Epileptic psychosis
^ESCTMA502396	231494001	Manic
^ESCTSE510608	237352005	Severe postnatal psychosis
^ESCTSC524896	247804008	Schizophrenic prodrome
^ESCTBI543590	260994008	Bipolar
^ESCTMI551797	270901009	Mixed schizophrenic and affective psychosis
^ESCTAC556819	274953007	Acute polymorphic psychotic disorder
^ESCTGE584078	297477009	Germanic language

^ESCTPR593370	304757008	Profile of mood states, bipolar
^ESCTLA689080	416340002	Late onset schizophrenia
^ESCTAC769626	712850003	Acute polymorphic psychotic disorder with symptoms of schizophrenia
^ESCTPS779024	719717006	Psychosis co-occurrent and due to Parkinson's disease
^ESCTCO785964	724689006	Cocaine-induced psychotic disorder
^ESCTRA804178	133091000119105	Rapid cycling bipolar I disorder
^ESCTMI823516	760721000000109	Mixed bipolar affective disorder, in partial remission
^ESCTSI823787	764741000000107	Single manic episode in remission

<u>Covariates medical codes</u>

BMI medical codes

Read code	Snomed concept Id	Medical term
22K	60621009	Body mass index
22K5	162864005	Body mass index 30+ - obesity
22K1	35425004	Normal body mass index
22K4	162863004	Body mass index index 25-29 - overweight
22K6	310252000	Body mass index less than 20
22K8	412768003	Body mass index 20-24 - normal
22K7	408512008	Body mass index 40+ - severely obese
22A4-1	275947003	O/E - overweight
222A	162690006	O/E - obese

22A5-1	162690006	O/E - obese
^ESCTBO455370	162863004	Body mass index 25-29 - overweight
22KC	914721000000105	Obese class I (body mass index 30.0 - 34.9)
22AA	238131007	Overweight
22KE	914741000000103	Obese class III (body mass index equal to or greater than 40.0)
22KD	914731000000107	Obese class II (body mass index 35.0 - 39.9)
^ESCTBM348480	60621009	BMI - Body mass index
22KB	846931000000101	Baseline body mass index
^ESCTBM455374	162864005	BMI 30+ - obesity
^ESCTBM455372	162863004	BMI 25-29 - overweight
^ESCTBM599828	310252000	BMI less than 20
^ESCTOV783399	722595002	Overweight in adulthood with body mass index of 25 or more but less than 30
^ESCTBA828699	846931000000101	Baseline BMI (body mass index)
^ESCTBO676339	408512008	Body mass index 40+ - morbidly obese
^ESCTBO706884	427090001	Body mass index less than 16.5
^ESCTWE348479	60621009	Weight: body mass
^ESCTNO307061	35425004	Normal BMI (body mass index)
^ESCTBM676340	408512008	BMI (body mass index) 40+ - severely obese
JHCBO5	1808071000006100	Body mass index 18.5-24.9
^ESCTOB589220	301331008	Observation of body mass index
EMISNQBO29	1808061000006100	Body mass index less than 18.5
PCNQBO1	923861000006108	Body mass index
^ESCT1192336	301331008	Finding of body mass index
EMISCUN18	981741000006106	Underweight: adult BMI 18.5; child BMI 5th percentile; or less
EMISCOV1	981731000006101	Overweight: adult BMI 25.0; child BMI 95th percentile; or more

EGTONMO1	857321000006109	Moderately obese
EGTONVE2	857911000006102	Very obese
^ESCTOB686699	414915002	Obese
^ESCTOB552644	271590003	Obese build
^ESCT1392667	819948005	Obese class III
^ESCT1211318	443381000124105	Obese class II
^ESCT1211316	443371000124107	Obese class I
^ESCTOB527306	249533007	Obese abdomen
HNGNQRF70	910251000006106	[RFC] Overweight
^ESCTPA511785	238131007	Patient overweight
^ESCTNO320328	43664005	Normal weight

Blood pressure medical codes

Read code	Snomed concept id	Medical term
246	163020007	O/E - blood pressure reading
2469	72313002	Systolic arterial pressure
246A	1091811000000100	Diastolic arterial pressure
2464	163025002	O/E - BP reading normal
246-1	163020007	O/E - BP reading
R1y2	24184005	Raised blood pressure
246d	413606001	Average home systolic blood pressure
246c	413605002	Average home diastolic blood pressure
662j	413153004	Blood pressure recorded by patient at home
315B	164783007	Ambulatory blood pressure recording

662V	135840009	Blood pressure monitoring
2465	163026001	O/E - BP borderline raised
2466	163027005	O/E - BP reading raised
246P	400975005	Standing diastolic blood pressure
246N	400974009	Standing systolic blood pressure
662L	170599006	24 hr blood pressure monitoring
246R	407555005	Sitting diastolic blood pressure
246Q	407554009	Sitting systolic blood pressure
246D	163034007	Standing blood pressure reading
246-2	163020007	O/E - blood pressure
G20-1	24184005	Elevated blood pressure
246L	315613000	Target diastolic blood pressure
246K	315612005	Target systolic blood pressure
ZV70B	274785000	[V]Examination of blood pressure
246E	163035008	Sitting blood pressure reading
7P1B2	448678005	Application of ambulatory blood pressure monitor
662Q	314956000	Borderline blood pressure
246V	314462001	Average 24 hour diastolic blood pressure
246W	314449000	Average 24 hour systolic blood pressure
8HR8	310353008	Referral for 24 hour blood pressure recording
246g	335661000000109	Self measured blood pressure reading
246X	314461008	Average day interval diastolic blood pressure
246Y	314446007	Average day interval systolic blood pressure
246Z	163020007	O/E-blood pressure reading NOS
246M	697930002	White coat hypertension
246a	314460009	Average night interval diastolic blood pressure
246b	314445006	Average night interval systolic blood pressure
246f	198091000000104	Ambulatory diastolic blood pressure
246e	198081000000101	Ambulatory systolic blood pressure

246T	407557002	Lying diastolic blood pressure
246S	407556006	Lying systolic blood pressure
246C	163033001	Lying blood pressure reading
246J	313005002	O/E - BP reading: no postural drop
8A59	924481000000109	Self-monitoring of blood pressure
8HRH	824421000000101	Referral for ambulatory blood pressure monitoring
7A551	42826002	Monitoring of arterial pressure
9OD-2	715051000000108	Blood pressure screen admin
EMISNQSY8	1808011000006110	Systolic blood pressure - left arm
R1y3	271870002	Low blood pressure reading
2463	163024003	O/E - BP borderline low
2467	163028000	O/E - BP reading very high
2468	163029008	O/E - BP reading:postural drop
EMISNQSY9	1808041000006110	Systolic blood pressure - right arm
^ESCTBL372560	75367002	Blood pressure
2462	163023009	O/E - BP reading low
246B	163032006	O/E - BP stable
EMISNQHO139	1994021000006100	Home systolic blood pressure
EMISNQDI86	1808021000006100	Diastolic blood pressure - left arm
EMISNQ242	1009241000006100	24 hr blood pressure normal
EMISNQHO138	1993951000006110	Home diastolic blood pressure
EMISNQDI87	1808051000006110	Diastolic blood pressure - right arm
^ESCT24604630	314463006	24 hour blood pressure
^ESCTNO253060	2004005	Normal blood pressure
EMISNQ241	1009231000006100	24 hr blood pressure abnormal
EMISCAB20	961921000006100	Abnormal blood pressure reading
246m	314453003	Average diastolic blood pressure
246l	314440001	Average systolic blood pressure
EMISNQSE154	2012461000006100	Self measured blood pressure reading required

246H	310356000	O/E - Arterial pressure index normal
2460	163021006	O/E - BP unrecordable
2461	163022004	O/E - BP reading very low
246G	163037000	O/E - BP labile
^ESCT1394902	860643007	No postural drop in blood pressure
246F	163036009	O/E - blood pressure decreased
EMISNQBL19	1805081000006100	Blood pressure monitoring in both arms
246n	928021000000108	Baseline blood pressure
246n0	716632005	Baseline diastolic blood pressure
246n1	716579001	Baseline systolic blood pressure
^ESCTEX556633	274785000	Examination of blood pressure
^ESCTBL473783	185673004	Blood pressure abnormal - 1st recall
^ESCTSI455671	163035008	Sitting blood pressure
^ESCTBP372561	75367002	BP - Blood pressure
^ESCTMO592977	304495004	Monitoring of blood pressure, temperature, pulse rate and respiratory rate
^ESCTSY552719	271649006	Systolic blood pressure
^ESCTST455669	163034007	Standing blood pressure
^ESCTME260839	6797001	Mean blood pressure
246I	310357009	O/E - Arterial pressure index abnormal
EMISNQBL40	2011941000006100	Blood pressure recorded in community
^ESCTMA529511	251074006	MAP - Mean arterial pressure
^ESCTDI552721	271650006	Diastolic blood pressure
^ESCTLO322617	45007003	Low blood pressure
EMISNQBL39	2011931000006110	Blood pressure recorded by pharmacy
246h	87179004	Arterial pulse pressure
^ESCTAP735856	448678005	Application of ABPM (ambulatory blood pressure monitor)
^ESCTSY367627	72313002	Systolic blood pressure
^ESCTNO529510	251074006	Non-invasive mean arterial pressure

246i	814081000000101	Diastolic blood pressure centile
246j	814101000000107	Systolic blood pressure centile
EMISCEL2	961661000006104	Elevated pulse/respirations/blood pressure
662s	722500002	Preoperative blood pressure measurement
^ESCTNO529512	251074006	Non-invasive mean blood pressure
246k	102584008	Unequal blood pressure in arms
^ESCTBL644506	385845001	Blood pressure taking assessment
^ESCTLY455667	163033001	Lying blood pressure
^ESCTBL325924	46973005	Blood pressure taking
^ESCTSY319048	42826002	Systemic arterial pressure monitoring
662t	722502005	Postoperative blood pressure measurement
^ESCTBL473785	185674005	Blood pressure abnormal - 2nd recall
^ESCTHI311741	38341003	High blood pressure
^ESCTAV784193	723232008	Average blood pressure
^ESCTME528714	250546000	Measurement of partial pressure of oxygen in blood
^ESCTEL635797	371622005	Elevated blood-pressure reading without diagnosis of hypertension
^ESCT24604632	314465004	24 hour diastolic blood pressure
^ESCT24604631	314464000	24 hour systolic blood pressure
^ESCTAR319051	42826002	Arterial pressure monitoring
^ESCTST552725	271651005	Stable blood pressure
^ESCTME836947	990241000000101	Measurement of blood pressure using standard adult size blood pressure cuff
^ESCTBL473788	185675006	Blood pressure abnormal - 3rd recall
^ESCTAB312665	38936003	Abnormal blood pressure
^ESCT1261950	1105331000000110	Blood pressure measurement using oscillometric monitoring device with opportunistic atrial fibrillation detection
246o	1036531000000110	Non-invasive central blood pressure
^ESCTSE810748	335661000000109	Self measured BP (blood pressure) reading

^ESCTNO539060	258057004	Non-invasive blood pressure monitor
^ESCTAR277209	17146006	Arterial pressure monitoring, non-invasive method
^ESCTNO270539	12929001	Normal systolic arterial pressure
^ESCTMA604626	314459004	Maximum 24 hour diastolic blood pressure
^ESCTMA604615	314448008	Maximum 24 hour systolic blood pressure
^ESCTMA604606	314439003	Maximum systolic blood pressure
^ESCTIN286917	23154005	Increased diastolic arterial pressure
^ESCTMI604621	314454009	Minimum day interval diastolic blood pressure
246o1	1036571000000100	Non-invasive central diastolic blood pressure
^ESCTMA604625	314458007	Maximum day interval diastolic blood pressure
^ESCTMA604624	314457002	Maximum night interval diastolic blood pressure
^ESCTMI604622	314455005	Minimum night interval diastolic blood pressure
^ESCTMA604611	314444005	Maximum day interval systolic blood pressure
^ESCTMA604610	314443004	Maximum night interval systolic blood pressure
^ESCTMI604609	314442009	Minimum night interval systolic blood pressure
^ESCTMI604608	314441002	Minimum day interval systolic blood pressure
246o0	1036551000000100	Non-invasive central systolic blood pressure
^ESCTAB645668	386534000	ABP - Arterial blood pressure
^ESCTMA604619	314452008	Maximum diastolic blood pressure
^ESCTON455630	163020007	On examination - blood pressure reading
^ESCTBP311745	38341003	BP - High blood pressure
^ESCTPO761507	707304009	Post exercise systolic blood pressure response normal
^ESCTMI604605	314438006	Minimum systolic blood pressure
EMISNQBL41	2018581000006110	Blood pressure recorded by optician
^ESCTME826407	816161000000103	Measurement of blood pressure using non-invasive blood pressure monitor
^ESCTME732836	446695008	Measurement of blood pressure at anterior tibial pulse using doppler
^ESCTBL535892	255330009	Blood pressure rise

^ESCTNO466071	174255007	Non-invasive diastolic arterial pressure
^ESCTBL435634	123820005	Blood oxygen pressure
^ESCTIN645664	386532001	Invasive arterial pressure
^ESCTFI588962	301140001	Finding of systemic arterial pressure
^ESCTNO337253	53813002	Normal diastolic arterial pressure
^ESCTME836946	990231000000105	Measurement of blood pressure using large adult size blood pressure cuff
^ESCTME836936	990151000000109	Measurement of blood pressure in right arm
^ESCTME836935	990141000000106	Measurement of blood pressure in left arm
^ESCTBL654365	392570002	Blood pressure finding
^ESCTSB645671	386536003	SBP - Systemic blood pressure
^ESCTEL635798	371622005	Elevated blood pressure reading without diagnosis of hypertension
^ESCTMI604623	314456006	Minimum 24 hour diastolic blood pressure
^ESCTMI604618	314451001	Minimum diastolic blood pressure
^ESCTMI604614	314447003	Minimum 24 hour systolic blood pressure
^ESCTNO529502	251070002	Non-invasive systolic arterial pressure
^ESCTPR463465	170573004	Pre-treatment blood pressure reading
^ESCTON455665	163032006	On examination - blood pressure stable
^ESCTON455653	163028000	On examination - blood pressure reading very high
^ESCTON455644	163025002	On examination - blood pressure reading normal
^ESCTNO270540	12929001	Normal systolic blood pressure
^ESCTSY625413	364090009	Systemic arterial pressure
^ESCTAB531027	252071000	ABI - Arterial pressure index
^ESCTAB330678	49844009	Abnormal diastolic arterial pressure
^ESCTDE318812	42689008	Decreased diastolic arterial pressure
^ESCTAB279169	18352002	Abnormal systolic arterial pressure
^ESCTHI278650	18050000	High systolic arterial pulse pressure
^ESCTIN278648	18050000	Increased systolic arterial pressure

Smoker medical codes

Read code	Snomed concept id	Medical term
1371	266919005	Never smoked tobacco
8CAL	225323000	Smoking cessation advice
137S	8517006	Ex-smoker
137P	65568007	Cigarette smoker
137R	77176002	Current smoker
137L	160618006	Current non-smoker
137K	160617001	Stopped smoking
1374	160604004	Moderate cigarette smoker (10-19 cigs/day)
1371-1	8392000	Non-smoker
1373	160603005	Light cigarette smoker (1-9 cigs/day)
137G	160616005	Trying to give up smoking
137T	160625004	Date ceased smoking
137	266918002	Tobacco smoking consumption
1379	266923002	Ex-moderate cigarette smoker (10-19/day)
137j	281018007	Ex-cigarette smoker
137F	8517006	Ex-smoker - amount unknown
1375	160605003	Heavy cigarette smoker (20-39 cigs/day)
137M	160619003	Rolls own cigarettes
1378	266922007	Ex-light cigarette smoker (1-9/day)
8H7i	395700008	Referral to smoking cessation advisor
9N2k	401068004	Seen by smoking cessation advisor
137P-1	77176002	Smoker
137A	266924008	Ex-heavy cigarette smoker (20-39/day)

137d	394873005	Not interested in stopping smoking
8IAj	527151000000107	Smoking cessation advice declined
137X	230056004	Cigarette consumption
137c	394871007	Thinking about stopping smoking
1372	266920004	Trivial cigarette smoker (less than one cigarette/day)
1377	266921000	Ex-trivial cigarette smoker (<1/day)
EGTON320	137811000006103	Smoking Status
137J	59978006	Cigar smoker
9OO1	185789006	Attends stop smoking monitoring
137n	228487000	Total time smoked
13p0	390901002	Negotiated date for cessation of smoking
137b	394872000	Ready to stop smoking
137g	401201003	Cigarette pack-years
137I	43381005	Passive smoker
137U	315213009	Not a passive smoker
13p5	401160008	Smoking cessation programme start date
8IEM	822591000000108	Smoking cessation drug therapy declined
137H	82302008	Pipe smoker
13WF4	161080002	Passive smoking risk
137B	266925009	Ex-very heavy cigarette smoker (40+/day)
13p	390900001	Smoking cessation milestones
13p1	390902009	Smoking status at 4 weeks
13WK	394964001	No smokers in the household
745H	710081004	Smoking cessation therapy
EGTONSM3	137771000006103	Smoking Age Started
1372-1	428041000124106	Occasional smoker
67H6	506491000000102	Brief intervention for smoking cessation
9OO	714001000000108	Anti-smoking monitoring admin.
137k	1098881000000100	Declined to give smoking status
1376	160606002	Very heavy cigarette smoker (40+ cigs/day)

137E	160614008	Tobacco consumption unknown
137Q	266929003	Smoking started
13p4	395177003	Smoking free weeks
137h	413173009	Minutes from waking to first tobacco consumption
745H4	713700008	Smoking cessation drug therapy
8CdB	783011000000105	Stop smoking service opportunity signposted
E251	89765005	Tobacco dependence
9OOB0	783401000000101	Stop smoking invitation first short message service text message
EMISQSM12	904181000006102	Smoking cessation counselling
137C	160612007	Keeps trying to stop smoking
67A3	171055003	Pregnancy smoking advice
137-1	266918002	Smoker - amount smoked
137Z	266918002	Tobacco consumption NOS
EGTONSM4	137761000006105	Smoking Age Ceased
EGTON322	649861000006105	Ex-Cigarette Smoker
EMISQSM18	852131000006108	Smoking cessation-practice nurse support
EMISQSM9	904011000006105	Smoking cessation declaration signed by patient
137m	446172000	Failed attempt to stop smoking
137N	160620009	Ex-pipe smoker
137O	160621008	Ex-cigar smoker
^ESCTTO628233	365981007	Tobacco smoking behaviour - finding
EMISHGT222	37311000006101	Other anti-smoking drug given
EMISQPR6	904021000006102	Previous smoking quit attempts
137V	134406006	Smoking reduced
745Hy	710081004	Other specified smoking cessation therapy
1782	340921000000103	Asthma trigger - tobacco smoke

9NdW	750851000000104	Consent given for smoking cessation data sharing
EGTON321	854021000006104	Cigarette smoker
137W	81703003	Chews tobacco
137Y	230057008	Cigar consumption
9ko-1	505651000000103	Current smoker annual review
9km-1	505761000000105	Ex-smoker annual review
137K0	517211000000106	Recently stopped smoking
EGTON1025	604961000006105	Current Smoker NOS
9OO-1	714021000000104	Stop smoking clinic administration
EMISQDA1	904091000006100	Date of last cigarette
EMISNQSM6	1626121000006100	Smoking cessation drug therapy - varenicline
137e	308438006	Smoking restarted
137f	401159003	Reason for restarting smoking
137l	492191000000103	Ex roll-up cigarette smoker
9kn-1	505681000000109	Non-smoker annual review
EGTON1027	649851000006108	Ex- Rolled Tobacco Smoker
13p50	712971000000108	Practice based smoking cessation programme start date
EMISQSM5	904151000006105	Smoking cessation 4 week F/U completed
EMISQSM15	904221000006106	Smoking cessation bupropion therapy
EMISQSM14	904241000006104	Smoking cessation counselling in person
HNGZ011	909391000006101	[RFC] Smoking cessation
ASDFGNO1	1009271000006100	Non Smoker - Nos
137I0	228524006	Exposed to tobacco smoke at home
137F-99	266928006	EX-Smoker NOS
137Q-1	308438006	Smoking restarted
137a	230058003	Pipe tobacco consumption
177	426936004	Smoke inhalation
137o	836001000000109	Waterpipe tobacco consumption
1PD	908781000000104	Ex user of electronic cigarette

^ESCTAS810907	340921000000103	Asthma trigger - tobacco smoke
EGTON328	854151000006107	Date stopped smoking
^ESCTWA806318	203191000000107	Wants to stop smoking
9kn	505681000000109	Non-smoker annual review - enhanced services administration
^ESCTSM822152	720401000000103	Smoking cessation
^ESCTNE549592	266919005	Never smoked
9NdZ	751661000000106	Declined consent for smoking cessation data sharing
EGTONGR10	854951000006109	Grade A non-smoker
EGTONGR11	854961000006106	Grade B light smoker (1-10/day)
EGTONGR12	854981000006101	Grade C moderate smoker (11-20/day)
EMISQSM4	904141000006108	Smoking cessation 4 week F/U not completed
EMISQSM11	904201000006101	Smoking cessation confidence score
137D	160613002	Admitted tobacco consumption possibly untrue
^ESCTEX549603	266928006	Ex-cigarette smoker amount unknown
EMISCSM1	961581000006105	Smokes/uses tobacco products
ZV4K0	110483000	Tobacco user
ZV4D7	229819007	Tobacco use and exposure
^ESCT1190499	230059006	Occasional cigarette smoker
^ESCTLI500315	230060001	Light cigarette smoker
^ESCTSM737599	449868002	Smokes tobacco daily
E2510	89765005	Tobacco dependence, unspecified
1V08	228378005	Smokes drugs in cigarette form
^ESCTMO500316	230062009	Moderate cigarette smoker
^ESCTHE500317	230063004	Heavy cigarette smoker
TDyy4	217580001	Accident caused by cigarette
E251z	89765005	Tobacco dependence NOS
^ESCTTO395911	89765005	Tobacco dependence syndrome
^ESCTOC815337	428041000124106	Occasional tobacco smoker

^ESCTEX801757	48031000119106	Ex-smoker for more than 1 year
EGTON1024	852981000006107	Rolls own cigarettes
^ESCT1172045	1092111000000100	Ex-light smoker (1-9/day)
^ESCT1172048	1092131000000110	Ex-trivial smoker (<1/day)
^ESCTHE342222	56771006	Heavy smoker (over 20 per day)
^ESCTCI407457	102408007	Cigarette smoke
E2511	191887008	Tobacco dependence, continuous
E2513	191889006	Tobacco dependence in remission
1V09	228379002	Smokes drugs through a pipe
^ESCTSM628235	365981007	Smoking
^ESCT1172039	1092071000000100	Ex-heavy smoker (20-39/day)
EMISNQSM16	1825071000006100	Smoking cessation 12 week F/U not completed
EGTON324	854051000006108	Ex-pipe smoker
^ESCT1172042	1092091000000110	Ex-moderate smoker (10-19/day)
EMISNQSM14	1819411000006100	Smoking increased
137i	228513009	Ex-tobacco chewer
SMC	212899006	Toxic effect of tobacco and nicotine
^ESCTEX319903	43381005	Exposed to tobacco smoke
^ESCTSM354414	64197008	Smoke
^ESCTEX498100	228525007	Exposed to tobacco smoke in public places
^ESCTVE500318	230064005	Very heavy cigarette smoker
ZV116	8517006	History of tobacco use
E2512	191888003	Tobacco dependence, episodic
^ESCTST736897	449369001	Stopped smoking before pregnancy
^ESCTTO314292	39953003	Tobacco
^ESCTCH500319	230065006	Chain smoker
^ESCTOC549595	266920004	Occasional cigarette smoker (less than one cigarette/day)

U271	284744004	[X]Intentional self harm by smoke, fire and flames, occurrence in residential institution
^ESCTCU671807	405746006	Current non smoker but past smoking history unknown
EMISQWA1	904041000006109	Waking time to first cigarette
EMISQSM16	904211000006103	Smoking cessation service patient satisfaction score
^ESCT1172036	1092041000000100	Ex-very heavy smoker (40+/day)
EMISNQSM15	1823811000006100	Smoking cessation 12 week follow up
EMISNQUS72	1879431000006110	User of electronic cigarette
^ESCTTO407456	102407002	Tobacco smoke
^ESCTCI558572	276468004	Cigarette burn
^ESCTNE498087	228512004	Never chewed tobacco
137L-99	160618006	Tobacco Consumption Nil
EMISQTH1	904031000006104	Thinking about stopping smoking
ALLERGY14708NEMIS	1120481000006110	Adverse reaction to Lactobacillus Acidophilus
J0364	9473008	Tobacco deposit on teeth
Eu173	90755006	[X]Mental and behavioural disorders due to use of tobacco: withdrawal state
^ESCTSM494853	225934006	Smokes in bed
^ESCTFI498083	228509002	Finding relating to tobacco chewing
^ESCTAM549590	266918002	Amount and type of tobacco smoked
^ESCTSM642793	384742004	Smoking cessation assistance
^ESCTNE756906	702979003	Never used tobacco
^ESCTCE263604	8517006	Cessation of smoking
^ESCTUS498078	228504007	User of moist powdered tobacco
0C3Z	159882006	Tobacco processor NOS
^ESCTCI783250	722496004	Cigarette
^ESCTST334508	52138004	Streptobacillary fever
^ESCTTR500314	230059006	Trivial cigarette smoker

^ESCTAG621728	360900008	Aggressive ex-smoker
PCSQI51	153868100006100	Smoke
^ESCTTO343057	57264008	Tobacco
^ESCTPI387464	84498003	Pipe smoking tobacco
^ESCTTO418033	110483000	Tobacco use
^ESCTDO498075	228501004	Does not use moist powdered tobacco
^ESCTDO498086	228511006	Does not chew tobacco
^ESCTIN621715	360890004	Intolerant ex-smoker
^ESCTTO628237	365982000	Tobacco smoking consumption - finding
^ESCT1179361	384742004	Smoking cessation behaviour support
^ESCTAS765081	709507008	Assessment of readiness for smoking cessation

Ethnicity medical codes

Read code	Snomed concept Id	Medical term
226	162730000	O/E - ethnic group
226-1	162730000	O/E - ethnic origin
9SB1	186020007	Other ethnic, Black/White orig
9SB2	186021006	Other ethnic, Asian/White orig
9SB4	186023009	Other ethnic, other mixed orig
9T	186034007	Ethnicity and other related nationality data
9T1	186035008	New Zealand ethnic groups
9SH	315281001	Other Asian ethnic group
9S14	401214002	Other white British ethnic group
9iA4	86461000000107	Sri Lankan - ethnic category 2001 census
9i2K	88971000000106	Albanian - ethnic category 2001 census
9iF6	88991000000105	Jewish - ethnic category 2001 census
9i4	92431000000100	White and Black African - ethnic category 2001 census

9iA	92481000000101	Other Asian background - ethnic category 2001 census
9i22	92551000000106	Welsh - ethnic category 2001 census
9i24	92561000000109	Northern Irish - ethnic category 2001 census
9i63	92601000000109	Chinese and White - ethnic category 2001 census
9iA2	92651000000105	Kashmiri - ethnic category 2001 census
9iA3	92661000000108	East African Asian - ethnic category 2001 census
9iA8	92681000000104	British Asian - ethnic category 2001 census
9iA7	92691000000102	Caribbean Asian - ethnic category 2001 census
9iD1	92731000000108	Nigerian - ethnic category 2001 census
9iF2	92771000000105	Filipino - ethnic category 2001 census
9i26	92791000000109	Cypriot (part not stated) - ethnic category 2001 census
9i27	93931000000104	Greek - ethnic category 2001 census
9i2J	93981000000100	Kosovan - ethnic category 2001 census
9i2M	94001000000108	Croatian - ethnic category 2001 census
9i2Q	94021000000104	Mixed Irish and other White - ethnic category 2001 census
9iFA	94061000000107	North African - ethnic category 2001 census
9iF4	94131000000103	Buddhist - ethnic category 2001 census
9iB	107691000000105	Caribbean - ethnic category 2001 census
9i20	110761000000106	English - ethnic category 2001 census
9iA6	110781000000102	Sinhalese - ethnic category 2001 census
9i2N	88981000000108	Serbian - ethnic category 2001 census
9i64	92611000000106	Asian and Chinese - ethnic category 2001 census
9iD3	92721000000106	Mixed Black - ethnic category 2001 census
9i2B	93961000000109	Italian - ethnic category 2001 census
9t1	976591000000101	Ethnic category - 2011 census Northern Ireland
9t01	976651000000108	White: Irish - England and Wales ethnic category 2011 census
9t20	977911000000103	White: Scottish - Scotland ethnic category 2011 census
9t24	978011000000101	White: Polish - Scotland ethnic category 2011 census
9SAD	186005001	Other ethnic NEC (NMO)

9T1Z	186035008	New Zealand ethnic group NOS
^ESCTOT474038	186022004	Other ethnic, mixed white origin
^ESCTBR474024	186006000	British ethnic minority specified (NMO)
^ESCTBR474026	186007009	British ethnic minority unspecified (NMO)
^ESCTET659190	397731000	Ethnicity
^ESCTET474006	185983003	Ethnic group (1991 census) (UK)
^ESCTET1163867	999002401000000000	Ethnic category - 2011 census Northern Ireland simple reference set
^ESCTET626090	364699009	Ethnic group
9SJ	372148003	Ethnic group
9i2H	88961000000104	Commonwealth of (Russian) Independent States - ethnic category 2001 census
9i2P	94011000000105	Other republics which made up the former Yugoslavia - ethnic category 2001 census
9iFB	94071000000100	Middle Eastern (excluding Israeli, Iranian and Arab) - ethnic category 2001 census
9iFJ	94121000000100	Multi-ethnic islands: Mauritian or Seychellois or Maldivian or St Helena - ethnic category 2001 census
9t00	976631000000101	White: English or Welsh or Scottish or Northern Irish or British - England and Wales ethnic category 2011 census
9t07	976771000000108	Mixed multiple ethnic groups: any other Mixed or multiple ethnic background - England and Wales ethnic category 2011 census
9t08	976791000000107	Asian or Asian British: Indian - England and Wales ethnic category 2011 census
9t09	976811000000108	Asian or Asian British: Pakistani - England and Wales ethnic category 2011 census
9t0D	976891000000104	Black or African or Caribbean or Black British: African - England and Wales ethnic category 2011 census
9t0E	976911000000101	Black or African or Caribbean or Black British: Caribbean - England and Wales ethnic category 2011 census

9t0G	976951000000102	Other ethnic group: Arab - England and Wales ethnic category 2011 census
9t16	977591000000103	Asian or Asian British: Indian - Northern Ireland ethnic category 2011 census
9t18	977731000000108	Asian or Asian British: Bangladeshi - Northern Ireland ethnic category 2011 census
9t1A	977771000000105	Asian or Asian British: any other Asian background - Northern Ireland ethnic category 2011 census
9t1B	977791000000109	Black or African or Caribbean or Black British: African - Northern Ireland ethnic category 2011 census
9t1D	977831000000102	Black or African or Caribbean or Black British: other Black or African or Caribbean background - Northern Ireland ethnic category 2011 census
9t1F	977871000000100	Other ethnic group: any other ethnic group - Northern Ireland ethnic category 2011 census
9t27	978071000000106	Asian or Asian Scottish or Asian British: Pakistani, Pakistani Scottish or Pakistani British - Scotland ethnic category 2011 census
9t2B	978211000000108	Asian or Asian Scottish or Asian British: any other Asian group - Scotland ethnic category 2011 census
9t2D	978251000000107	African: any other African - Scotland ethnic category 2011 census
9t2H	978381000000105	Other ethnic group: Arab, Arab Scottish or Arab British - Scotland ethnic category 2011 census
9t2J	978401000000105	Other ethnic group: any other ethnic group - Scotland ethnic category 2011 census
9t04	976711000000103	Mixed multiple ethnic groups: White and Black Caribbean - England and Wales ethnic category 2011 census
9t0A	976831000000100	Asian or Asian British: Bangladeshi - England and Wales ethnic category 2011 census

9t0F	976931000000109	Black or African or Caribbean or Black British: other Black or African or Caribbean background - England and Wales ethnic category 2011 census
9t17	977711000000100	Asian or Asian British: Pakistani - Northern Ireland ethnic category 2011 census
9t19	977751000000101	Asian or Asian British: Chinese - Northern Ireland ethnic category 2011 census
9t2A	978191000000109	Asian or Asian Scottish or Asian British: Chinese - Scotland ethnic category 2011 census
9t2G	978361000000101	Caribbean or Black: any other Black or Caribbean group - Scotland ethnic category 2011 census
^ESCTET1163866	999002391000000000	Ethnic category - 2011 census England and Wales simple reference set
^ESCTFI627041	365456003	Finding of ethnicity / related nationality data
ESCTBL5	315240009	Black - ethnic group
ESCTMI5	315239007	Mixed ethnic census group
9TC	718958002	Roma ethnic group
9i2F	88941000000100	Polish - ethnic category 2001 census
9i1	92401000000106	Irish - ethnic category 2001 census
9i3	92421000000102	White and Black Caribbean - ethnic category 2001 census
9i21	92541000000108	Scottish - ethnic category 2001 census
9iA5	92671000000101	Tamil - ethnic category 2001 census
9iD0	92711000000100	Somali - ethnic category 2001 census
9i25	93921000000101	Ulster Scots - ethnic category 2001 census
9iFE	94091000000101	Kurdish - ethnic category 2001 census
9iF8	94141000000107	Sikh - ethnic category 2001 census
9i0	92391000000108	British or mixed British - ethnic category 2001 census
9SA1	186006000	Brit. ethnic minor. spec.(NMO)
9SB3	186022004	Other ethnic, mixed white orig
^ESCTET659760	398089004	Ethnic background

9T1E	315280000	Asian - ethnic group
9S12	185984009	White - ethnic group
^ESCTET822482	723621000000103	Ethnicity
^ESCTON455110	162730000	On examination - ethnic group
9T1Y	186035008	Other New Zealand ethnic group
^ESCTET627039	365455004	Ethnic groups (1991 census) (United Kingdom)
226Z	162730000	O/E - ethnic group NOS
9t10	977351000000100	White - Northern Ireland ethnic category 2011 census
9t21	977931000000106	White: other British - Scotland ethnic category 2011 census
9i	92381000000106	Ethnic category - 2001 census
9iF1	92761000000103	Japanese - ethnic category 2001 census
9i2L	93991000000103	Bosnian - ethnic category 2001 census
9i9	92471000000103	Bangladeshi or British Bangladeshi - ethnic category 2001 census
9t13	977411000000108	Mixed multiple ethnic groups: White and Black African - Northern Ireland ethnic category 2011 census
9t15	977551000000106	Mixed multiple ethnic groups: any other Mixed or multiple ethnic background - Northern Ireland ethnic category 2011 census
9t25	978031000000109	White: any other White ethnic group - Scotland ethnic category 2011 census
^ESCTET474007	185983003	Ethnic groups (1991 census)
^ESCTET627040	365456003	Ethnicity / related nationality data - finding
^ESCTOT474034	186020007	Other ethnic, Black/White origin
^ESCTOT474040	186023009	Other ethnic, other mixed origin
^ESCTET659191	397731000	Ethnic groups
^ESCTET627038	365455004	Ethnic groups (1991 census) (UK)
9S	397731000	Ethnic group finding
9i2T	94051000000109	Other White or White unspecified - ethnic category 2001 census

9t02	976671000000104	White: Gypsy or Irish Traveller - England and Wales ethnic category 2011 census
9t06	976751000000104	Mixed multiple ethnic groups: White and Asian - England and Wales ethnic category 2011 census
9t0H	976971000000106	Other ethnic group: any other ethnic group - England and Wales ethnic category 2011 census
9t11	977371000000109	Irish Traveller - Northern Ireland ethnic category 2011 census
9t1C	977811000000105	Black or African or Caribbean or Black British: Caribbean - Northern Ireland ethnic category 2011 census
9t23	977971000000108	White: Gypsy or Irish Traveller - Scotland ethnic category 2011 census
9t29	978171000000105	Asian or Asian Scottish or Asian British: Bangladeshi, Bangladeshi Scottish or Bangladeshi British - Scotland ethnic category 2011 census
9t2C	978231000000100	African: African, African Scottish or African British - Scotland ethnic category 2011 census
9t2E	978271000000103	Caribbean or Black: Caribbean, Caribbean Scottish or Caribbean British - Scotland ethnic category 2011 census
9t2F	978341000000102	Caribbean or Black: Black, Black Scottish or Black British - Scotland ethnic category 2011 census
9t0	976571000000100	Ethnic category - 2011 census England and Wales
9t22	977951000000104	White: Irish - Scotland ethnic category 2011 census
9i00	494131000000105	White British - ethnic category 2001 census
9i2E	88931000000109	Gypsy/Romany - ethnic category 2001 census
9i8	92461000000105	Pakistani or British Pakistani - ethnic category 2001 census
9i60	92581000000100	Black and Asian - ethnic category 2001 census
9i61	92591000000103	Black and Chinese - ethnic category 2001 census
9iA9	92631000000103	Mixed Asian - ethnic category 2001 census
9iF0	92751000000101	Vietnamese - ethnic category 2001 census
9i2A	93951000000106	Turkish Cypriot - ethnic category 2001 census
9i29	110401000000103	Turkish - ethnic category 2001 census

9iF5	110831000000107	Hindu - ethnic category 2001 census
^ESCTET474008	185983003	Ethnic group (1991 census) (United Kingdom)
^ESCTET474042	186034007	Ethnicity / related nationality data
9SZ	397731000	Ethnic groups (census) NOS
9SG	315279003	Other black ethnic group
9SB	186019001	Other ethnic, mixed origin
9i2C	88911000000101	Irish Traveller - ethnic category 2001 census
9i2D	88921000000107	Traveller - ethnic category 2001 census
9iFH	89021000000101	South and Central American - ethnic category 2001 census
9iF7	89031000000104	Muslim - ethnic category 2001 census
9i2	92411000000108	Other White background - ethnic category 2001 census
9i6	92451000000107	Other Mixed background - ethnic category 2001 census
9iC	92491000000104	African - ethnic category 2001 census
9iE	92511000000107	Chinese - ethnic category 2001 census
9iF	92521000000101	Other - ethnic category 2001 census
9i23	92571000000102	Cornish - ethnic category 2001 census
9iA1	92641000000107	Punjabi - ethnic category 2001 census
9iF3	92781000000107	Malaysian - ethnic category 2001 census
9i28	93941000000108	Greek Cypriot - ethnic category 2001 census
9iFG	94111000000106	Latin American - ethnic category 2001 census
9i62	110771000000104	Black and White - ethnic category 2001 census
9iD	92501000000105	Other Black background - ethnic category 2001 census
^ESCTET1163868	999002411000000000	Ethnic category - 2011 census Scotland simple reference set
9i2R	94041000000106	Other White European or European unspecified or Mixed European - ethnic category 2001 census
9iD4	92741000000104	Other Black or Black unspecified - ethnic category 2001 census
9t03	976691000000100	White: any other White background - England and Wales ethnic category 2011 census

9t05	976731000000106	Mixed multiple ethnic groups: White and Black African - England and Wales ethnic category 2011 census
9t0C	976871000000103	Asian or Asian British: any other Asian background - England and Wales ethnic category 2011 census
9t1E	977851000000109	Other ethnic group: Arab - Northern Ireland ethnic category 2011 census
9t26	978051000000102	Mixed or multiple ethnic groups: any Mixed or multiple ethnic group - Scotland ethnic category 2011 census
^ESCTPE658974	397607002	Perioperative plan of care consistent with patient value system, lifestyle, ethnicity, and culture
^ESCTET636480	372148003	Ethnic category
^ESCTOT474036	186021006	Other ethnic, Asian/White origin
9T1A	372148003	Other Pacific ethnic group
9t	976551000000109	Ethnic category - 2011 census
9t2	976611000000109	Ethnic category - 2011 census Scotland
9iF9	89001000000105	Arab - ethnic category 2001 census
9iFD	89011000000107	Iranian - ethnic category 2001 census
9i5	92441000000109	White and Asian - ethnic category 2001 census
9i2S	94031000000102	Other mixed White - ethnic category 2001 census
9iFC	94081000000103	Israeli - ethnic category 2001 census
9iFF	94101000000109	Moroccan - ethnic category 2001 census
9iFK	94151000000105	Any other group - ethnic category 2001 census
9i7	110751000000108	Indian or British Indian - ethnic category 2001 census
9iD2	110791000000100	Black British - ethnic category 2001 census
9SA	186005001	Other ethnic non-mixed (NMO)
9SA2	186007009	Brit. ethnic minor. unsp (NMO)
9i10	494161000000100	White Irish - ethnic category 2001 census
9i2G	88951000000102	Baltic States (Estonian or Latvian or Lithuanian) - ethnic category 2001 census

9i65	92621000000100	Other Mixed or Mixed unspecified - ethnic category 2001 census
9iAA	92701000000102	Other Asian or Asian unspecified - ethnic category 2001 census
9t0B	976851000000107	Asian or Asian British: Chinese - England and Wales ethnic category 2011 census
9t12	977391000000108	Mixed multiple ethnic groups: White and Black Caribbean - Northern Ireland ethnic category 2011 census
9t14	977431000000100	Mixed multiple ethnic groups: White and Asian - Northern Ireland ethnic category 2011 census
9t28	978111000000100	Asian or Asian Scottish or Asian British: Indian, Indian Scottish or Indian British - Scotland ethnic category 2011 census
