# Supplementary material for: Severe mental illness, race/ethnicity, multimorbidity and mortality following COVID-19 infection: nationally representative cohort study
Source: Br J Psychiatry. 2023 Nov;223(5):518–25. doi: 10.1192/bjp.2023.112 (PMC7615273; doi:10.1192/bjp.2023.112)
Supplement: Supplementary file 1 [file S0007125023001125sup001.docx]

**Title:** Severe mental illnesses, multimorbidity and mortality following COVID-19 infection: Nationally representative cohort study 

**Authors:** Jayati Das-Munshi PhD, Ioannis Bakolis PhD, Laia Bécares PhD, Jacqui Dyer PhD, Matthew Hotopf PhD, Josephine Ocloo PhD, Robert Stewart MD, Ruth Stuart MSc, Alex Dregan PhD 

**Supplementary material**

**Figures:5**

**Supplementary Figure 1: Hazard Ratios for the association of severe mental illnesses (SMI) with all-cause mortality, following COVID-19 infection. Complete case models**

**Key:** Displayed estimates are from complete case models. SMI- severe mental illness. ‘Fully adjusted’ models adjusted for age, sex, race/ ethnicity, UK regions, deprivation, BMI, smoking status and all comorbidities displayed in Table 1 and an interaction term between SMI*multimorbidity.

**Supplementary Figure 2: Covariates adjusted survival probability following COVID-19 infection by severe mental illness (SMI) status, over the first year of the COVID-19 pandemic**

**Supplementary Figure 3: Hazard Ratios for the association of severe mental illnesses (SMI) with all-cause mortality, following COVID-19 infection in patients aged 18 years or over at the time of COVID-19 pandemic.**

**Table S1. Baseline demographic and clinical characteristics for the matched sample with positive COVID-19 test results, by Severe Mental Illness (SMI) status**

|  | **No SMI group** | **SMI group** |
| --- | --- | --- |
| **Sample size** | **35,575** | **7,146** |
|  | N (%)/ mean (SD) | N (%)/ mean (SD) |
| **Age,** mean (SD) | 55(19) | 55(19) |
| **Sex** |  |  |
| Female | 20,381(57) | 4,088(57) |
| **Body Mass Index (BMI)** |  |  |
| Optimal (17.5-24.9) | 9,982(28) | 1,851(26) |
| Underweight (<17.5) | 814(2) | 223(3) |
| Overweight (25-29.9) | 11,385(32) | 2,041(29) |
| Obese (30+) | 10,511(30) | 2,757(38) |
| Missing | 2,883(8) | 270(4) |
| **Smoker** |  |  |
| Never | 20,248(57) | 3,272(46) |
| Former | 8,706(24) | 1,728(24) |
| Current | 6,011(17) | 2,100(29) |
| Missing | 610(2) | 42(1) |
| **Area deprivation*** |  |  |
| Least deprived | 5,364(15) | 786(11) |
| Second | 5,878(17) | 1,075(15) |
| Third | 6,311(18) | 1,210(17) |
| Fourth | 7,715(21) | 1,575(22) |
| Most deprived | 8,804(25) | 2,077(29) |
| Missing | 1,503(4) | 423(6) |
| **Race/ Ethnicity** |  |  |
| White British/ Irish/ White Other | 23,423(66) | 4,777(67) |
| Black Caribbean/ Black African/ Black Other | 1,287(4) | 415(6) |
| Indian, Pakistani, Bangladeshi | 3,875(11) | 690(11) |
| Mixed ethnicity | 389(1) | 122(2) |
| Other | 1,638(5) | 362(5) |
| Missing | 4,963(14) | 776(11) |
| **UK Regions** |  |  |
| London | 7,361(21) | 1,688(24) |
| North West England | 8,499(24) | 1,634(23) |
| North East England | 2,443(6) | 458(6) |
| West Midlands | 6,309(18) | 1,233(17) |
| East Midlands | 1,764(5) | 319(4) |
| South-East England | 6,340(18) | 1,255(18) |
| South-West England | 2,422(7) | 465(7) |
| Northern Ireland | 61(<1) | 17(<1) |
| Missing | 376(1) | 73(1) |
| **Multimorbidities/ Long term health conditions** |  |  |
| Hypertension | 9,449(27) | 1,806(25) |
| Myocardial infarction | 1,033(3) | 190(3) |
| Heart disease | 1,314(4) | 248(3) |
| Ischemic Stroke | 1,686(5) | 492(7) |
| Diabetes | 4,409(12) | 1,336(19) |
| Cancer | 3,681(10) | 666(9) |
| Liver disease | 1,288(4) | 410(6) |
| Kidney disease | 3,513(10) | 931(13) |
| Chronic Obstructive Pulmonary Disease (COPD) | 2,509(7) | 677(9) |
| Asthma | 5,818(16) | 1,501 (21) |
| Autoimmune | 3,227(9) | 651(9) |
| Substance use | 540(2) | 835(12) |
| Epilepsy | 950(3) | 607(9) |
| Depression | 8,157(23) | 3,783(53) |
| Anxiety | 7,545(21) | 3,017(42) |
| Eating disorders | 387(1) | 287(4) |
| Gastroesophageal reflux disorder | 2,127(6) | 462(6) |
| Dementia | 865(2) | 265(4) |
| Immunological drug therapy | 14,728(41) | 2,806(39) |
| Corticosteroid drugs | 6,032(17) | 1,074(15) |
| Admissions to intensive care unit | 42(0.12) | 26(0.34) |

**Supplementary Figure 4: Hazard Ratios for the association of severe mental illnesses (SMI) with all-cause mortality, following COVID-19 infection. Multimorbidity matched patients**

**SMI medical codes**

| Read code | Snomed concept id | Medical term |
| --- | --- | --- |
| E1005 | 4926007 | Schizophrenia in remission |
| E13y1 | 5464005 | Brief reactive psychosis |
| E1001 | 16990005 | Subchronic schizophrenia |
| E106 | 26025008 | Residual schizophrenia |
| E1032 | 31658008 | Chronic paranoid schizophrenia |
| E1021 | 42868002 | Subchronic catatonic schizophrenia |
| E1035 | 63181006 | Paranoid schizophrenia in remission |
| E103 | 64905009 | Paranoid schizophrenia |
| E1022 | 68995007 | Chronic catatonic schizophrenia |
| E141 | 71961003 | Disintegrative psychosis |
| E1031 | 79866005 | Subchronic paranoid schizophrenia |
| 1464 | 161468000 | H/O: schizophrenia |
| E0111 | 191471000 | Korsakov's alcoholic psychosis with peripheral neuritis |
| E01y | 42344001 | Other alcoholic psychosis |
| E01yz | 42344001 | Other alcoholic psychosis NOS |
| E01z | 42344001 | Alcoholic psychosis NOS |
| E02z | 191483003 | Drug psychosis NOS |
| E04z | 191447007 | Chronic organic psychosis NOS |
| E10 | 191526005 | Schizophrenic disorders |
| E100-1 | 191527001 | Schizophrenia simplex |
| E100 | 191527001 | Simple schizophrenia |
| E1000 | 58214004 | Unspecified schizophrenia |
| E1003 | 111482003 | Acute exacerbation of subchronic schizophrenia |
| E1004 | 191531007 | Acute exacerbation of chronic schizophrenia |
| E100z | 191527001 | Simple schizophrenia NOS |
| E1010 | 35252006 | Unspecified hebephrenic schizophrenia |
| E1011 | 27387000 | Subchronic hebephrenic schizophrenia |
| E1012 | 12939007 | Chronic hebephrenic schizophrenia |
| E1013 | 14291003 | Acute exacerbation of subchronic hebephrenic schizophrenia |
| E1014 | 191539009 | Acute exacerbation of chronic hebephrenic schizophrenia |
| E101z | 35252006 | Hebephrenic schizophrenia NOS |
| E102 | 191542003 | Catatonic schizophrenia |
| Eu202-4 | 191542003 | [X]Schizophrenic flexibilatis cerea |
| E1020 | 191542003 | Unspecified catatonic schizophrenia |
| E1023 | 191547009 | Acute exacerbation of subchronic catatonic schizophrenia |
| E1024 | 191548004 | Acute exacerbation of chronic catatonic schizophrenia |
| E102z | 191542003 | Catatonic schizophrenia NOS |
| E1030 | 64905009 | Unspecified paranoid schizophrenia |
| E1033 | 191554003 | Acute exacerbation of subchronic paranoid schizophrenia |
| E1034 | 191555002 | Acute exacerbation of chronic paranoid schizophrenia |
| E103z | 64905009 | Paranoid schizophrenia NOS |
| E105 | 191559008 | Latent schizophrenia |
| E1050 | 191559008 | Unspecified latent schizophrenia |
| E1051 | 191561004 | Subchronic latent schizophrenia |
| E1052 | 191562006 | Chronic latent schizophrenia |
| E1053 | 191563001 | Acute exacerbation of subchronic latent schizophrenia |
| E1054 | 191564007 | Acute exacerbation of chronic latent schizophrenia |
| E105z | 191559008 | Latent schizophrenia NOS |
| E107-1 | 191567000 | Cyclic schizophrenia |
| E10y1 | 191577003 | Coenesthopathic schizophrenia |
| E10y-1 | 191577003 | Cenesthopathic schizophrenia |
| E10yz | 58214004 | Other schizophrenia NOS |
| E10z | 58214004 | Schizophrenia |
| E1100 | 268619003 | Single manic episode, unspecified |
| E1101 | 191583000 | Single manic episode, mild |
| E1102 | 191584006 | Single manic episode, moderate |
| E1103 | 764641000000104 | Single manic episode, severe |
| E1104 | 191586008 | Single manic episode, severe, with psychosis |
| E1105 | 764731000000103 | Single manic episode in partial remission |
| E110z | 268619003 | Manic disorder, single episode NOS |
| E111 | 191590005 | Recurrent manic episodes |
| E1110 | 191590005 | Recurrent manic episodes, unspecified |
| E1111 | 191592002 | Recurrent manic episodes, mild |
| E1112 | 191593007 | Recurrent manic episodes, moderate |
| E1114 | 191595000 | Recurrent manic episodes, severe, with psychosis |
| E111z | 191590005 | Recurrent manic episode NOS |
| E114-1 | 191618007 | Manic-depressive - now manic |
| E1140 | 191618007 | Bipolar affective disorder, currently manic, unspecified |
| E1141 | 191620005 | Bipolar affective disorder, currently manic, mild |
| E1142 | 191621009 | Bipolar affective disorder, currently manic, moderate |
| E114z | 191618007 | Bipolar affective disorder, currently manic, NOS |
| E115-1 | 191627008 | Manic-depressive - now depressed |
| E1150 | 191627008 | Bipolar affective disorder, currently depressed, unspecified |
| E1151 | 191629006 | Bipolar affective disorder, currently depressed, mild |
| E1152 | 191630001 | Bipolar affective disorder, currently depressed, moderate |
| E115z | 191627008 | Bipolar affective disorder, currently depressed, NOS |
| E116 | 191636007 | Mixed bipolar affective disorder |
| E1160 | 191636007 | Mixed bipolar affective disorder, unspecified |
| E1161 | 191638008 | Mixed bipolar affective disorder, mild |
| E1162 | 191639000 | Mixed bipolar affective disorder, moderate |
| E1164 | 191641004 | Mixed bipolar affective disorder, severe, with psychosis |
| E116z | 191636007 | Mixed bipolar affective disorder, NOS |
| E117 | 13746004 | Unspecified bipolar affective disorder |
| E1170 | 13746004 | Unspecified bipolar affective disorder, unspecified |
| E1171 | 13313007 | Mild bipolar disorder |
| E1172 | 79584002 | Moderate bipolar disorder |
| E117z | 13746004 | Unspecified bipolar affective disorder, NOS |
| E11y | 13746004 | Other and unspecified manic-depressive psychoses |
| E11y0 | 13746004 | Unspecified manic-depressive psychoses |
| E11y1 | 191658009 | Atypical manic disorder |
| E11y3 | 16506000 | Mixed bipolar I disorder |
| E11yz | 13746004 | Other and unspecified manic-depressive psychoses NOS |
| E11z | 441704009 | Affective psychosis |
| E11zz | 441704009 | Other affective psychosis NOS |
| E12z | 191667009 | Paranoid psychosis |
| E130 | 191676002 | Reactive depressive psychosis |
| E131 | 191677006 | Acute hysterical psychosis |
| E134 | 191680007 | Psychogenic paranoid psychosis |
| E141z | 71961003 | Disintegrative psychosis NOS |
| Eu2 | 417601000000102 | [X]Schizophrenia, schizotypal and delusional disorders |
| Eu203 | 111484002 | Undifferentiated schizophrenia |
| Eu20z | 58214004 | [X]Schizophrenia, unspecified |
| Eu22z | 231487004 | [X]Persistent delusional disorder, unspecified |
| Eu23y | 231489001 | [X]Other acute and transient psychotic disorders |
| Eu25y | 68890003 | [X]Other schizoaffective disorders |
| Eu25z | 68890003 | [X]Schizoaffective disorder, unspecified |
| Eu301 | 231494001 | Mania |
| Eu30y | 231494001 | [X]Other manic episodes |
| Eu31z | 13746004 | [X]Bipolar affective disorder, unspecified |
| E13z-1 | 69322001 | Psychotic disorder |
| E00y-1 | 231438001 | Presbyophrenic psychosis |
| E14z-1 | 191687005 | Childhood schizophrenia NOS |
| E104 | 268617001 | Acute schizophrenic episode |
| E10y | 58214004 | Other schizophrenia |
| E110 | 268619003 | Manic disorder, single episode |
| E121 | 268622001 | Chronic paranoid psychosis |
| E1z | 191525009 | Non-organic psychosis NOS |
| E14z | 191687005 | Child psychosis NOS |
| Eu20y | 58214004 | [X]Other schizophrenia |
| Eu21 | 31027006 | Schizotypal personality disorder |
| Eu22y | 231487004 | [X]Other persistent delusional disorders |
| Eu233 | 231489001 | [X]Other acute predominantly delusional psychotic disorders |
| Eu23z | 231489001 | [X]Acute and transient psychotic disorder, unspecified |
| Eu2z | 191525009 | [X]Unspecified nonorganic psychosis |
| Eu302 | 231494001 | [X]Mania with psychotic symptoms |
| Eu30z | 268619003 | [X]Manic episode, unspecified |
| Eu31y | 13746004 | [X]Other bipolar affective disorders |
| 285-1 | 268957000 | Psychotic condition, insight present |
| ZV110 | 161464003 | [V]Personal history of schizophrenia |
| E10y0 | 111484002 | Atypical schizophrenia |
| E1002 | 83746006 | Chronic schizophrenic |
| E1070 | 191567000 | Unspecified schizo-affective schizophrenia |
| E1175 | 5703000 | Bipolar disorder in partial remission |
| E1173 | 53049002 | Severe bipolar disorder without psychotic features |
| E1174 | 4441000 | Severe bipolar disorder with psychotic features |
| E1071 | 191569002 | Subchronic schizo-affective schizophrenia |
| E107 | 191567000 | Schizoaffective schizophrenia |
| E1075 | 191574005 | Schizoaffective schizophrenia in remission |
| E107z | 191567000 | Schizo-affective schizophrenia NOS |
| E2122 | 31027006 | Schizotypal personality |
| E106-1 | 26025008 | Restzustand - schizophrenia |
| E1115 | 764671000000105 | Recurrent manic episodes, in partial remission |
| E1113 | 764621000000106 | Recurrent manic episodes, severe |
| Eu220 | 48500005 | Delusional disorder |
| Eu220-1 | 191667009 | [X]Paranoid psychosis |
| E11-3 | 231494001 | Manic psychosis |
| E13z | 191525009 | Non-organic psychosis |
| 8HHs | 199191000000109 | Referral to psychosis early intervention service |
| Eu02z-2 | 268612007 | [X] Presenile psychosis NOS |
| Eu02z-5 | 268612007 | [X] Senile psychosis NOS |
| Eu04-3 | 191502008 | [X]Acute / subacute infective psychosis |
| Eu23 | 231489001 | Acute transient psychotic disorder |
| Eu231 | 712850003 | Acute polymorphic psychotic disorder co-occurrent with symptoms of schizophrenia |
| Eu230 | 712824002 | Acute polymorphic psychotic disorder without symptoms of schizophrenia |
| Eu232 | 278853003 | Acute schizophrenia-like psychotic disorder |
| Eu3z-1 | 46206005 | [X]Affective psychosis NOS |
| Eu105-4 | 42344001 | [X]Alcoholic psychosis NOS |
| Eu841-1 | 231536004 | [X]Atypical childhood psychosis |
| Eu203-1 | 111484002 | [X]Atypical schizophrenia |
| Eu314 | 61403008 | Severe depressed bipolar I disorder without psychotic features |
| Eu312 | 191623007 | Bipolar affective disorder, currently manic, severe, with psychosis |
| Eu311 | 191618007 | [X]Bipolar affect disorder cur epi manic wout psychotic symp |
| Eu313 | 191630001 | [X]Bipolar affect disorder cur epi mild or moderate depressn |
| Eu31 | 13746004 | Bipolar affective disorder |
| Eu310 | 31446002 | Bipolar affective disorder, current episode hypomanic |
| Eu316 | 192362008 | [X]Bipolar affective disorder, current episode mixed |
| Eu30-1 | 268619003 | [X]Bipolar disorder, single manic episode |
| Eu31y-1 | 83225003 | [X]Bipolar II disorder |
| Eu21-2 | 274952002 | Borderline schizophrenia |
| Eu231-1 | 712850003 | [X]Bouffee delirante with symptoms of schizophrenia |
| Eu23z-1 | 231489001 | [X]Brief reactive psychosis NOS |
| Eu232-1 | 278853003 | [X]Brief schizophreniform disorder |
| Eu232-2 | 278853003 | [X]Brief schizophrenifrm psych |
| Eu202 | 191542003 | [X]Catatonic schizophrenia |
| Eu202-1 | 191542003 | [X]Catatonic stupor |
| Eu20y-1 | 58214004 | [X]Cenesthopathic schizophrenia |
| Eu2y-1 | 480111000000107 | [X]Chronic hallucinatory psychosis |
| Eu205-1 | 111484002 | [X]Chronic undifferentiated schizophrenia |
| Eu252-1 | 270901009 | [X]Cyclic schizophrenia |
| Eu230-2 | 307417003 | [X]Cycloid psychosis |
| Eu231-2 | 307417003 | Cycloid psychosis |
| Eu340 | 76105009 | Cyclothymia |
| Eu22y-1 | 231487004 | [X]Delusional dysmorphophobia |
| Eu843-2 | 35919005 | [X]Disintegrative psychosis |
| Eu201-1 | 35252006 | [X]Disorganised schizophrenia |
| Eu05y-1 | 111479008 | [X]Epileptic psychosis NOS |
| Eu201 | 35252006 | [X]Hebephrenic schizophrenia |
| Eu300 | 231496004 | Hypomania |
| Eu44-4 | 44376007 | [X]Hysterical psychosis |
| Eu24-2 | 61831009 | [X]Induced paranoid disorder |
| Eu24-3 | 61831009 | [X]Induced psychotic disorder |
| Eu840-3 | 408858002 | [X]Infantile psychosis |
| Eu22y-2 | 231487004 | [X]Involutional paranoid state |
| Eu106-1 | 69482004 | Korsakoff's psychosis |
| Eu03-1 | 3298001 | [X]Korsakov's psychosis, nonalcoholic |
| Eu21-3 | 191559008 | [X]Latent schizophrenia |
| Eu21-1 | 191559008 | [X]Latent schizophrenic reaction |
| Eu31-3 | 13746004 | [X]Manic-depressive reaction |
| Eu30z-1 | 268619003 | [X]Mania NOS |
| Eu302-1 | 231494001 | [X]Mania with mood-congruent psychotic symptoms |
| Eu302-2 | 231494001 | [X]Mania with mood-incongruent psychotic symptoms |
| Eu30 | 268619003 | [X]Manic episode |
| Eu302-3 | 231494001 | [X]Manic stupor |
| Eu332-3 | 36474008 | [X] Manic-depressive psychosis, depressed type without psychotic symptoms |
| Eu333-2 | 765176007 | [X]Manic-depress psychosis,depressed type+psychotic symptoms |
| Eu31-1 | 13746004 | Manic-depressive illness |
| Eu31-2 | 13746004 | Manic-depressive psychosis |
| Eu125 | 943081000000102 | Cannabis-induced psychosis |
| Eu105 | 42344001 | Alcohol-induced psychosis |
| Eu145 | 943101000000108 | Cocaine-induced psychosis |
| Eu115 | 943071000000104 | Opioid-induced psychosis |
| Eu3y0-1 | 192362008 | Bipolar affective disorder , current episode mixed |
| Eu252-2 | 270901009 | Mixed schizophrenic and affective pschosis |
| Eu232-3 | 278853003 | [X]Oneirophrenia |
| Eu052 | 5510009 | Organic delusional disorder |
| Eu0z-1 | 111479008 | [X]Organic psychosis NOS |
| Eu2y | 191525009 | [X]Other nonorganic psychotic disorders |
| Eu22y-3 | 231487004 | [X]Paranoia querulans |
| Eu200 | 64905009 | [X]Paranoid schizophrenia |
| Eu200-1 | 64905009 | [X]Paraphrenic schizophrenia |
| Eu22 | 231487004 | Persistent delusional disorder |
| Eu21-4 | 247804008 | Prepsychotic schizophrenia |
| Eu21-5 | 247804008 | Prodromal schizophrenia |
| Eu21-6 | 31027006 | [X]Pseudoneurotic schizophrenia |
| Eu21-7 | 31027006 | [X]Pseudopsychopathic schizophrenia |
| Eu233-2 | 191680007 | [X]Psychogenic paranoid psychosis |
| Eu2z-1 | 69322001 | Psychotic |
| Eu531-1 | 199260001 | [X]Puerperal psychosis NOS |
| Eu23z-2 | 231437006 | [X]Reactive psychosis |
| Eu333-4 | 191613003 | [X]Recurr severe episodes/psychogenic depressive psychosis |
| Eu31y-2 | 191590005 | [X]Recurrent manic episodes |
| Eu205 | 26025008 | [X]Residual schizophrenia |
| Eu205-2 | 26025008 | [X]Restzustand schizophrenic |
| Eu251 | 84760002 | Schizoaffective disorder, depressive type |
| Eu250 | 271428004 | Schizoaffective disorder, manic type |
| Eu252 | 270901009 | Schizoaffective disorder, mixed type |
| Eu25 | 68890003 | Schizoaffective disorder |
| Eu25z-1 | 68890003 | [X]Schizoaffective psychosis NOS |
| Eu251-1 | 84760002 | [X]Schizoaffective psychosis, depressive type |
| Eu250-1 | 271428004 | [X]Schizoaffective psychosis, manic type |
| Eu20 | 58214004 | [X]Schizophrenia |
| Eu052-2 | 5510009 | [X]Schizophrenia-like psychosis in epilepsy |
| Eu202-2 | 191542003 | [X]Schizophrenic catalepsy |
| Eu202-3 | 191542003 | [X]Schizophrenic catatonia |
| Eu232-4 | 278853003 | [X]Schizophrenic reaction |
| Eu20y-2 | 58214004 | [X]Schizophreniform disord NOS |
| Eu251-2 | 84760002 | [X]Schizophreniform psychosis, depressive type |
| Eu250-2 | 271428004 | [X]Schizophreniform psychosis, manic type |
| Eu20y-3 | 58214004 | [X]Schizophrenifrm psychos NOS |
| Eu21-8 | 31027006 | [X]Schizotypal personality disorder |
| Eu206 | 191527001 | [X]Simple schizophrenia |
| Eu843-4 | 35919005 | [X]Symbiotic psychosis |
| E1074 | 191572009 | Acute exacerbation of chronic schizo-affective schizophrenia |
| E1073 | 191571002 | Acute exacerbation subchronic schizo-affective schizophrenia |
| E01 | 42344001 | Alcohol-induced psychosis |
| E1143 | 162004 | Severe manic bipolar I disorder without psychotic features |
| E1144 | 191623007 | Bipolar affect disord, currently manic,severe with psychosis |
| E1155 | 49512000 | Depressed bipolar I disorder in partial remission |
| E1154 | 765176007 | Bipolar affect disord, now depressed, severe with psychosis |
| E1153 | 61403008 | Bipolar affect disord, now depressed, severe, no psychosis |
| E1145 | 63249007 | Manic bipolar I disorder in partial remission |
| E115 | 191627008 | Bipolar affective disorder, current episode depression |
| E114 | 191618007 | Bipolar affective disorder, current episode manic |
| E11-1 | 13746004 | Bipolar disorder |
| E1072 | 191570001 | Chronic schizoaffective schizophrenia |
| E02 | 191483003 | Drug psychosis |
| E1165 | 760721000000109 | Mixed bipolar affective disorder, partial/unspec remission |
| E1163 | 764591000000108 | Mixed bipolar affective disorder, severe |
| E0110 | 69482004 | Korsakov alcoholic psychosis |
| E110-1 | 231496004 | Hypomanic psychoses |
| E101 | 35252006 | Hebephrenic schizophrenia |
| EMISQHY1 | 851691000006105 | Hypomanic |
| EGTON118 | 853201000006100 | Obsessional compulsive psychosis |
| E03y3-99 | 551591000000100 | Puerperal psychosis |
| E10-98 | 191526005 | Schizophrenic psychoses NOS |
| E10-99 | 191526005 | Schizophrenic psychoses |
| E107-99 | 191567000 | Acute schizo affective psychosis |
| E11-99 | 13746004 | Manic-depressive psychoses |
| E110-99 | 268619003 | Mania/hypomania |
| 212T | 200951000000109 | Psychosis, schizophrenia and bipolar affective disorder resolved |
| Eu318 | 371596008 | Bipolar I disorder |
| Eu319 | 83225003 | Bipolar II disorder |
| Eu319-1 | 83225003 | [X]Bipolar II disorder |
| EMISICD10│F0630 | 58329000 | Organic manic disorder |
| EMISICD10│F0631 | 1972101000006110 | Organic bipolar affective disorder |
| EMISICD10│F2000 | 1974351000006100 | Paranoid schizophrenia, continuous |
| EMISICD10│F2001 | 1974391000006110 | Paranoid schizophrenia, episodic with progressive deficit |
| EMISICD10│F2002 | 1974431000006100 | Paranoid schizophrenia, episodic with stable deficit |
| EMISICD10│F2003 | 1974451000006110 | Paranoid schizophrenia, episodic remittent |
| EMISICD10│F2004 | 1974481000006100 | Paranoid schizophrenia, incomplete remission |
| EMISICD10│F2009 | 1974541000006110 | Paranoid schizophrenia, course uncertain, period of observation too short |
| EMISICD10│F2010 | 1974601000006110 | Hebephrenic schizophrenia, continuous |
| EMISICD10│F2011 | 1974611000006100 | Hebephrenic schizophrenia, episodic with progressive deficit |
| EMISICD10│F2012 | 1974621000006100 | Hebephrenic schizophrenia, episodic with stable deficit |
| EMISICD10│F2013 | 1974641000006110 | Hebephrenic schizophrenia, episodic remittent |
| EMISICD10│F2014 | 1974671000006100 | Hebephrenic schizophrenia, incomplete remission |
| EMISICD10│F2019 | 1974721000006100 | Hebephrenic schizophrenia, course uncertain, period of observation too short |
| EMISICD10│F2020 | 1974751000006100 | Catatonic schizophrenia, continuous |
| EMISICD10│F2021 | 1974781000006110 | Catatonic schizophrenia, episodic with progressive deficit |
| EMISICD10│F2022 | 1974801000006110 | Catatonic schizophrenia, episodic with stable deficit |
| EMISICD10│F2023 | 1974831000006100 | Catatonic schizophrenia, episodic remittent |
| EMISICD10│F2024 | 1974861000006110 | Catatonic schizophrenia, incomplete remission |
| EMISICD10│F2029 | 1974921000006110 | Catatonic schizophrenia, course uncertain, period of observation too short |
| EMISICD10│F2034 | 1975051000006100 | Undifferentiated schizophrenia, incomplete remission |
| EMISICD10│F2050 | 1975351000006100 | Residual schizophrenia, continuous |
| EMISICD10│F2051 | 1975381000006110 | Residual schizophrenia, episodic with progressive deficit |
| EMISICD10│F2052 | 1975401000006110 | Residual schizophrenia, episodic with stable deficit |
| EMISICD10│F2053 | 1975431000006100 | Residual schizophrenia, episodic remittent |
| EMISICD10│F2054 | 1975441000006110 | Residual schizophrenia, incomplete remission |
| EMISICD10│F2059 | 1975481000006100 | Residual schizophrenia, course uncertain, period of observation too short |
| EMISICD10│F2060 | 1975491000006100 | Simple schizophrenia, continuous |
| EMISICD10│F2061 | 1975501000006100 | Simple schizophrenia, episodic with progressive deficit |
| EMISICD10│F2062 | 1975521000006100 | Simple schizophrenia, episodic with stable deficit |
| EMISICD10│F2063 | 1975551000006110 | Simple schizophrenia, episodic remittent |
| EMISICD10│F2064 | 1975571000006100 | Simple schizophrenia, incomplete remission |
| EMISICD10│F2300 | 1975621000006100 | Acute polymorphic psychot disord without symp of schizoph, without associated acute stress |
| EMISICD10│F2069 | 1975641000006110 | Simple schizophrenia, course uncertain, period of observation too short |
| EMISICD10│F2301 | 1975671000006100 | Acute polymorphic psychot disord without symp of schizoph, with associated acute stress |
| EMISICD10│F2310 | 1975681000006100 | Acute polymorphic psychot disord with symp of schizophren, without associated acute stress |
| EMISICD10│F2311 | 1975691000006100 | Acute polymorphic psychot disord with symp of schizophren, with associated acute stress |
| EMISICD10│F2320 | 1975711000006100 | Acute schizophrenia-like psychotic disorder, without associated acute stress |
| EMISICD10│F2321 | 1975731000006110 | Acute schizophrenia-like psychotic disorder, with associated acute stress |
| EMISICD10│F2080 | 1975751000006100 | Other schizophrenia, continuous |
| EMISICD10│F2330 | 1975761000006100 | Other acute predominantly delusional psychotic disorders, without associated acute stress |
| EMISICD10│F2081 | 1975771000006100 | Other schizophrenia, episodic with progressive deficit |
| EMISICD10│F2331 | 1975781000006110 | Other acute predominantly delusional psychotic disorders, with associated acute stress |
| EMISICD10│F2082 | 1975801000006110 | Other schizophrenia, episodic with stable deficit |
| EMISICD10│F2380 | 1975821000006100 | Other acute and transient psychotic disorders, without associated acute stress |
| EMISICD10│F2083 | 1975831000006100 | Other schizophrenia, episodic remittent |
| EMISICD10│F2084 | 1975841000006110 | Other schizophrenia, incomplete remission |
| EMISICD10│F2381 | 1975851000006110 | Other acute and transient psychotic disorders, with associated acute stress |
| EMISICD10│F2390 | 1975871000006100 | Acute and transient psychotic disorder, unspecified, without associated acute stress |
| EMISICD10│F2391 | 1975901000006100 | Acute and transient psychotic disorder, unspecified, with associated acute stress |
| EMISICD10│F2089 | 1975911000006100 | Other schizophrenia, course uncertain, period of observation too short |
| EMISICD10│F3130 | 1975931000006110 | Bipolar affect disorder cur epi mild or moderate depressn, without somatic syndrome |
| EMISICD10│F3131 | 1975941000006100 | Bipolar affect disorder cur epi mild or moderate depressn, with somatic syndrome |
| EMISICD10│F2090 | 1976001000006110 | Schizophrenia, unspecified, continuous |
| EMISICD10│F2091 | 1976061000006100 | Schizophrenia, unspecified, episodic with progressive deficit |
| EMISICD10│F2092 | 1976101000006110 | Schizophrenia, unspecified, episodic with stable deficit |
| EMISICD10│F2093 | 1976121000006100 | Schizophrenia, unspecified, episodic remittent |
| EMISICD10│F2094 | 1976141000006100 | Schizophrenia, unspecified, incomplete remission |
| EMISICD10│F2099 | 1976171000006100 | Schizophrenia, unspecified, course uncertain, period of observation too short |
| EMISICD10│F2008 | 1976881000006110 | Paranoid schizophrenia, other |
| EMISICD10│F2018 | 1976891000006110 | Hebephrenic schizophrenia, other |
| EMISICD10│F2028 | 1976901000006100 | Catatonic schizophrenia, other |
| EMISICD10│F2038 | 1976911000006110 | Undifferentiated schizophrenia, other |
| EMISICD10│F2058 | 1976931000006100 | Residual schizophrenia, other |
| EMISICD10│F2068 | 1976941000006110 | Simple schizophrenia, other |
| EMISICD10│F2088 | 1976951000006110 | Other schizophrenia, other |
| EMISICD10│F2098 | 1976961000006110 | Schizophrenia, unspecified, other |
| ^ESCTBR258680 | 5464005 | Brief psychotic disorder |
| ^ESCT1171785 | 1089671000000110 | Mania with mood-incongruent psychotic features |
| ^ESCT1171786 | 1089681000000110 | Mania with psychotic features |
| ^ESCT1171787 | 1089691000000100 | Acute predominantly delusional psychotic disorder |
| ^ESCTSC300026 | 31027006 | Schizoptypal disorder |
| ^ESCTBI300667 | 31446002 | Bipolar I disorder, most recent episode hypomanic |
| ^ESCTDI306812 | 35252006 | Disorganised schizophrenia |
| ^ESCTSC311804 | 38368003 | Schizoaffective disorder, bipolar type |
| ^ESCTUN313726 | 39610001 | Undifferentiated schizophrenia in remission |
| ^ESCTOR344778 | 58329000 | Organic mood disorder of manic type |
| ^ESCTSE346839 | 59617007 | Severe bipolar I disorder, most recent episode depressed with psychotic features |
| ^ESCTIN350472 | 61831009 | Induced psychotic disorder |
| ^ESCTIN350475 | 61831009 | Induced psychosis |
| ^ESCTIN350479 | 61831009 | Induced paranoid disorder |
| ^ESCTPS362734 | 69322001 | Psychosis |
| ^ESCTKO363012 | 69482004 | Korsakoff psychosis |
| ^ESCTKO363015 | 69482004 | Korsakov psychosis |
| ^ESCTCY373801 | 76105009 | Cyclothymic disorder |
| ^ESCTBI385440 | 83225003 | Bipolar 2 disorder |
| ^ESCTCH386266 | 83746006 | Chronic schizophrenia |
| ^ESCTSC394614 | 88975006 | Schizophreniform disorder |
| ^ESCTSC408293 | 102940002 | Schizophrenic reaction |
| ^ESCTHI453993 | 161468000 | History of schizophrenia |
| ^ESCTDR476321 | 191483003 | Drug-induced psychosis |
| ^ESCTAC476364 | 191539009 | Acute exacerbation of chronic disorganised schizophrenia |
| ^ESCTAC476391 | 191571002 | Acute exacerbation of subchronic schizoaffective schizophrenia |
| ^ESCTAC476392 | 191572009 | Acute exacerbation of chronic schizoaffective schizophrenia |
| ^ESCTPS476451 | 191687005 | Psychosis with origin in childhood |
| ^ESCTBI476731 | 192362008 | Bipolar affective disorder, current episode mixed |
| ^ESCTOR502345 | 231444002 | Organic bipolar disorder |
| ^ESCTEP502353 | 231449007 | Epileptic psychosis |
| ^ESCTMA502396 | 231494001 | Manic |
| ^ESCTSE510608 | 237352005 | Severe postnatal psychosis |
| ^ESCTSC524896 | 247804008 | Schizophrenic prodrome |
| ^ESCTBI543590 | 260994008 | Bipolar |
| ^ESCTMI551797 | 270901009 | Mixed schizophrenic and affective psychosis |
| ^ESCTAC556819 | 274953007 | Acute polymorphic psychotic disorder |
| ^ESCTGE584078 | 297477009 | Germanic language |
| ^ESCTPR593370 | 304757008 | Profile of mood states, bipolar |
| ^ESCTLA689080 | 416340002 | Late onset schizophrenia |
| ^ESCTAC769626 | 712850003 | Acute polymorphic psychotic disorder with symptoms of schizophrenia |
| ^ESCTPS779024 | 719717006 | Psychosis co-occurrent and due to Parkinson's disease |
| ^ESCTCO785964 | 724689006 | Cocaine-induced psychotic disorder |
| ^ESCTRA804178 | 133091000119105 | Rapid cycling bipolar I disorder |
| ^ESCTMI823516 | 760721000000109 | Mixed bipolar affective disorder, in partial remission |
| ^ESCTSI823787 | 764741000000107 | Single manic episode in remission |

**Covariates medical codes**

**BMI medical codes**

| Read code | Snomed concept Id | Medical term |
| --- | --- | --- |
| 22K | 60621009 | Body mass index |
| 22K5 | 162864005 | Body mass index 30+ - obesity |
| 22K1 | 35425004 | Normal body mass index |
| 22K4 | 162863004 | Body mass index index 25-29 - overweight |
| 22K6 | 310252000 | Body mass index less than 20 |
| 22K8 | 412768003 | Body mass index 20-24 - normal |
| 22K7 | 408512008 | Body mass index 40+ - severely obese |
| 22A4-1 | 275947003 | O/E - overweight |
| 222A | 162690006 | O/E - obese |
| 22A5-1 | 162690006 | O/E - obese |
| ^ESCTBO455370 | 162863004 | Body mass index 25-29 - overweight |
| 22KC | 914721000000105 | Obese class I (body mass index 30.0 - 34.9) |
| 22AA | 238131007 | Overweight |
| 22KE | 914741000000103 | Obese class III (body mass index equal to or greater than 40.0) |
| 22KD | 914731000000107 | Obese class II (body mass index 35.0 - 39.9) |
| ^ESCTBM348480 | 60621009 | BMI - Body mass index |
| 22KB | 846931000000101 | Baseline body mass index |
| ^ESCTBM455374 | 162864005 | BMI 30+ - obesity |
| ^ESCTBM455372 | 162863004 | BMI 25-29 - overweight |
| ^ESCTBM599828 | 310252000 | BMI less than 20 |
| ^ESCTOV783399 | 722595002 | Overweight in adulthood with body mass index of 25 or more but less than 30 |
| ^ESCTBA828699 | 846931000000101 | Baseline BMI (body mass index) |
| ^ESCTBO676339 | 408512008 | Body mass index 40+ - morbidly obese |
| ^ESCTBO706884 | 427090001 | Body mass index less than 16.5 |
| ^ESCTWE348479 | 60621009 | Weight: body mass |
| ^ESCTNO307061 | 35425004 | Normal BMI (body mass index) |
| ^ESCTBM676340 | 408512008 | BMI (body mass index) 40+ - severely obese |
| JHCBO5 | 1808071000006100 | Body mass index 18.5-24.9 |
| ^ESCTOB589220 | 301331008 | Observation of body mass index |
| EMISNQBO29 | 1808061000006100 | Body mass index less than 18.5 |
| PCNQBO1 | 923861000006108 | Body mass index |
| ^ESCT1192336 | 301331008 | Finding of body mass index |
| EMISCUN18 | 981741000006106 | Underweight: adult BMI 18.5; child BMI 5th percentile; or less |
| EMISCOV1 | 981731000006101 | Overweight: adult BMI 25.0; child BMI 95th percentile; or more |
| EGTONMO1 | 857321000006109 | Moderately obese |
| EGTONVE2 | 857911000006102 | Very obese |
| ^ESCTOB686699 | 414915002 | Obese |
| ^ESCTOB552644 | 271590003 | Obese build |
| ^ESCT1392667 | 819948005 | Obese class III |
| ^ESCT1211318 | 443381000124105 | Obese class II |
| ^ESCT1211316 | 443371000124107 | Obese class I |
| ^ESCTOB527306 | 249533007 | Obese abdomen |
| HNGNQRF70 | 910251000006106 | [RFC] Overweight |
| ^ESCTPA511785 | 238131007 | Patient overweight |
| ^ESCTNO320328 | 43664005 | Normal weight |

**Blood pressure medical codes**

| Read code | Snomed concept id | Medical term |
| --- | --- | --- |
| 246 | 163020007 | O/E - blood pressure reading |
| 2469 | 72313002 | Systolic arterial pressure |
| 246A | 1091811000000100 | Diastolic arterial pressure |
| 2464 | 163025002 | O/E - BP reading normal |
| 246-1 | 163020007 | O/E - BP reading |
| R1y2 | 24184005 | Raised blood pressure |
| 246d | 413606001 | Average home systolic blood pressure |
| 246c | 413605002 | Average home diastolic blood pressure |
| 662j | 413153004 | Blood pressure recorded by patient at home |
| 315B | 164783007 | Ambulatory blood pressure recording |
| 662V | 135840009 | Blood pressure monitoring |
| 2465 | 163026001 | O/E - BP borderline raised |
| 2466 | 163027005 | O/E - BP reading raised |
| 246P | 400975005 | Standing diastolic blood pressure |
| 246N | 400974009 | Standing systolic blood pressure |
| 662L | 170599006 | 24 hr blood pressure monitoring |
| 246R | 407555005 | Sitting diastolic blood pressure |
| 246Q | 407554009 | Sitting systolic blood pressure |
| 246D | 163034007 | Standing blood pressure reading |
| 246-2 | 163020007 | O/E - blood pressure |
| G20-1 | 24184005 | Elevated blood pressure |
| 246L | 315613000 | Target diastolic blood pressure |
| 246K | 315612005 | Target systolic blood pressure |
| ZV70B | 274785000 | [V]Examination of blood pressure |
| 246E | 163035008 | Sitting blood pressure reading |
| 7P1B2 | 448678005 | Application of ambulatory blood pressure monitor |
| 662Q | 314956000 | Borderline blood pressure |
| 246V | 314462001 | Average 24 hour diastolic blood pressure |
| 246W | 314449000 | Average 24 hour systolic blood pressure |
| 8HR8 | 310353008 | Referral for 24 hour blood pressure recording |
| 246g | 335661000000109 | Self measured blood pressure reading |
| 246X | 314461008 | Average day interval diastolic blood pressure |
| 246Y | 314446007 | Average day interval systolic blood pressure |
| 246Z | 163020007 | O/E-blood pressure reading NOS |
| 246M | 697930002 | White coat hypertension |
| 246a | 314460009 | Average night interval diastolic blood pressure |
| 246b | 314445006 | Average night interval systolic blood pressure |
| 246f | 198091000000104 | Ambulatory diastolic blood pressure |
| 246e | 198081000000101 | Ambulatory systolic blood pressure |
| 246T | 407557002 | Lying diastolic blood pressure |
| 246S | 407556006 | Lying systolic blood pressure |
| 246C | 163033001 | Lying blood pressure reading |
| 246J | 313005002 | O/E - BP reading: no postural drop |
| 8A59 | 924481000000109 | Self-monitoring of blood pressure |
| 8HRH | 824421000000101 | Referral for ambulatory blood pressure monitoring |
| 7A551 | 42826002 | Monitoring of arterial pressure |
| 9OD-2 | 715051000000108 | Blood pressure screen admin |
| EMISNQSY8 | 1808011000006110 | Systolic blood pressure - left arm |
| R1y3 | 271870002 | Low blood pressure reading |
| 2463 | 163024003 | O/E - BP borderline low |
| 2467 | 163028000 | O/E - BP reading very high |
| 2468 | 163029008 | O/E - BP reading:postural drop |
| EMISNQSY9 | 1808041000006110 | Systolic blood pressure - right arm |
| ^ESCTBL372560 | 75367002 | Blood pressure |
| 2462 | 163023009 | O/E - BP reading low |
| 246B | 163032006 | O/E - BP stable |
| EMISNQHO139 | 1994021000006100 | Home systolic blood pressure |
| EMISNQDI86 | 1808021000006100 | Diastolic blood pressure - left arm |
| EMISNQ242 | 1009241000006100 | 24 hr blood pressure normal |
| EMISNQHO138 | 1993951000006110 | Home diastolic blood pressure |
| EMISNQDI87 | 1808051000006110 | Diastolic blood pressure - right arm |
| ^ESCT24604630 | 314463006 | 24 hour blood pressure |
| ^ESCTNO253060 | 2004005 | Normal blood pressure |
| EMISNQ241 | 1009231000006100 | 24 hr blood pressure abnormal |
| EMISCAB20 | 961921000006100 | Abnormal blood pressure reading |
| 246m | 314453003 | Average diastolic blood pressure |
| 246l | 314440001 | Average systolic blood pressure |
| EMISNQSE154 | 2012461000006100 | Self measured blood pressure reading required |
| 246H | 310356000 | O/E - Arterial pressure index normal |
| 2460 | 163021006 | O/E - BP unrecordable |
| 2461 | 163022004 | O/E - BP reading very low |
| 246G | 163037000 | O/E - BP labile |
| ^ESCT1394902 | 860643007 | No postural drop in blood pressure |
| 246F | 163036009 | O/E - blood pressure decreased |
| EMISNQBL19 | 1805081000006100 | Blood pressure monitoring in both arms |
| 246n | 928021000000108 | Baseline blood pressure |
| 246n0 | 716632005 | Baseline diastolic blood pressure |
| 246n1 | 716579001 | Baseline systolic blood pressure |
| ^ESCTEX556633 | 274785000 | Examination of blood pressure |
| ^ESCTBL473783 | 185673004 | Blood pressure abnormal - 1st recall |
| ^ESCTSI455671 | 163035008 | Sitting blood pressure |
| ^ESCTBP372561 | 75367002 | BP - Blood pressure |
| ^ESCTMO592977 | 304495004 | Monitoring of blood pressure, temperature, pulse rate and respiratory rate |
| ^ESCTSY552719 | 271649006 | Systolic blood pressure |
| ^ESCTST455669 | 163034007 | Standing blood pressure |
| ^ESCTME260839 | 6797001 | Mean blood pressure |
| 246I | 310357009 | O/E - Arterial pressure index abnormal |
| EMISNQBL40 | 2011941000006100 | Blood pressure recorded in community |
| ^ESCTMA529511 | 251074006 | MAP - Mean arterial pressure |
| ^ESCTDI552721 | 271650006 | Diastolic blood pressure |
| ^ESCTLO322617 | 45007003 | Low blood pressure |
| EMISNQBL39 | 2011931000006110 | Blood pressure recorded by pharmacy |
| 246h | 87179004 | Arterial pulse pressure |
| ^ESCTAP735856 | 448678005 | Application of ABPM (ambulatory blood pressure monitor) |
| ^ESCTSY367627 | 72313002 | Systolic blood pressure |
| ^ESCTNO529510 | 251074006 | Non-invasive mean arterial pressure |
| 246i | 814081000000101 | Diastolic blood pressure centile |
| 246j | 814101000000107 | Systolic blood pressure centile |
| EMISCEL2 | 961661000006104 | Elevated pulse/respirations/blood pressure |
| 662s | 722500002 | Preoperative blood pressure measurement |
| ^ESCTNO529512 | 251074006 | Non-invasive mean blood pressure |
| 246k | 102584008 | Unequal blood pressure in arms |
| ^ESCTBL644506 | 385845001 | Blood pressure taking assessment |
| ^ESCTLY455667 | 163033001 | Lying blood pressure |
| ^ESCTBL325924 | 46973005 | Blood pressure taking |
| ^ESCTSY319048 | 42826002 | Systemic arterial pressure monitoring |
| 662t | 722502005 | Postoperative blood pressure measurement |
| ^ESCTBL473785 | 185674005 | Blood pressure abnormal - 2nd recall |
| ^ESCTHI311741 | 38341003 | High blood pressure |
| ^ESCTAV784193 | 723232008 | Average blood pressure |
| ^ESCTME528714 | 250546000 | Measurement of partial pressure of oxygen in blood |
| ^ESCTEL635797 | 371622005 | Elevated blood-pressure reading without diagnosis of hypertension |
| ^ESCT24604632 | 314465004 | 24 hour diastolic blood pressure |
| ^ESCT24604631 | 314464000 | 24 hour systolic blood pressure |
| ^ESCTAR319051 | 42826002 | Arterial pressure monitoring |
| ^ESCTST552725 | 271651005 | Stable blood pressure |
| ^ESCTME836947 | 990241000000101 | Measurement of blood pressure using standard adult size blood pressure cuff |
| ^ESCTBL473788 | 185675006 | Blood pressure abnormal - 3rd recall |
| ^ESCTAB312665 | 38936003 | Abnormal blood pressure |
| ^ESCT1261950 | 1105331000000110 | Blood pressure measurement using oscillometric monitoring device with opportunistic atrial fibrillation detection |
| 246o | 1036531000000110 | Non-invasive central blood pressure |
| ^ESCTSE810748 | 335661000000109 | Self measured BP (blood pressure) reading |
| ^ESCTNO539060 | 258057004 | Non-invasive blood pressure monitor |
| ^ESCTAR277209 | 17146006 | Arterial pressure monitoring, non-invasive method |
| ^ESCTNO270539 | 12929001 | Normal systolic arterial pressure |
| ^ESCTMA604626 | 314459004 | Maximum 24 hour diastolic blood pressure |
| ^ESCTMA604615 | 314448008 | Maximum 24 hour systolic blood pressure |
| ^ESCTMA604606 | 314439003 | Maximum systolic blood pressure |
| ^ESCTIN286917 | 23154005 | Increased diastolic arterial pressure |
| ^ESCTMI604621 | 314454009 | Minimum day interval diastolic blood pressure |
| 246o1 | 1036571000000100 | Non-invasive central diastolic blood pressure |
| ^ESCTMA604625 | 314458007 | Maximum day interval diastolic blood pressure |
| ^ESCTMA604624 | 314457002 | Maximum night interval diastolic blood pressure |
| ^ESCTMI604622 | 314455005 | Minimum night interval diastolic blood pressure |
| ^ESCTMA604611 | 314444005 | Maximum day interval systolic blood pressure |
| ^ESCTMA604610 | 314443004 | Maximum night interval systolic blood pressure |
| ^ESCTMI604609 | 314442009 | Minimum night interval systolic blood pressure |
| ^ESCTMI604608 | 314441002 | Minimum day interval systolic blood pressure |
| 246o0 | 1036551000000100 | Non-invasive central systolic blood pressure |
| ^ESCTAB645668 | 386534000 | ABP - Arterial blood pressure |
| ^ESCTMA604619 | 314452008 | Maximum diastolic blood pressure |
| ^ESCTON455630 | 163020007 | On examination - blood pressure reading |
| ^ESCTBP311745 | 38341003 | BP - High blood pressure |
| ^ESCTPO761507 | 707304009 | Post exercise systolic blood pressure response normal |
| ^ESCTMI604605 | 314438006 | Minimum systolic blood pressure |
| EMISNQBL41 | 2018581000006110 | Blood pressure recorded by optician |
| ^ESCTME826407 | 816161000000103 | Measurement of blood pressure using non-invasive blood pressure monitor |
| ^ESCTME732836 | 446695008 | Measurement of blood pressure at anterior tibial pulse using doppler |
| ^ESCTBL535892 | 255330009 | Blood pressure rise |
| ^ESCTNO466071 | 174255007 | Non-invasive diastolic arterial pressure |
| ^ESCTBL435634 | 123820005 | Blood oxygen pressure |
| ^ESCTIN645664 | 386532001 | Invasive arterial pressure |
| ^ESCTFI588962 | 301140001 | Finding of systemic arterial pressure |
| ^ESCTNO337253 | 53813002 | Normal diastolic arterial pressure |
| ^ESCTME836946 | 990231000000105 | Measurement of blood pressure using large adult size blood pressure cuff |
| ^ESCTME836936 | 990151000000109 | Measurement of blood pressure in right arm |
| ^ESCTME836935 | 990141000000106 | Measurement of blood pressure in left arm |
| ^ESCTBL654365 | 392570002 | Blood pressure finding |
| ^ESCTSB645671 | 386536003 | SBP - Systemic blood pressure |
| ^ESCTEL635798 | 371622005 | Elevated blood pressure reading without diagnosis of hypertension |
| ^ESCTMI604623 | 314456006 | Minimum 24 hour diastolic blood pressure |
| ^ESCTMI604618 | 314451001 | Minimum diastolic blood pressure |
| ^ESCTMI604614 | 314447003 | Minimum 24 hour systolic blood pressure |
| ^ESCTNO529502 | 251070002 | Non-invasive systolic arterial pressure |
| ^ESCTPR463465 | 170573004 | Pre-treatment blood pressure reading |
| ^ESCTON455665 | 163032006 | On examination - blood pressure stable |
| ^ESCTON455653 | 163028000 | On examination - blood pressure reading very high |
| ^ESCTON455644 | 163025002 | On examination - blood pressure reading normal |
| ^ESCTNO270540 | 12929001 | Normal systolic blood pressure |
| ^ESCTSY625413 | 364090009 | Systemic arterial pressure |
| ^ESCTAB531027 | 252071000 | ABI - Arterial pressure index |
| ^ESCTAB330678 | 49844009 | Abnormal diastolic arterial pressure |
| ^ESCTDE318812 | 42689008 | Decreased diastolic arterial pressure |
| ^ESCTAB279169 | 18352002 | Abnormal systolic arterial pressure |
| ^ESCTHI278650 | 18050000 | High systolic arterial pulse pressure |
| ^ESCTIN278648 | 18050000 | Increased systolic arterial pressure |

**Smoker medical codes**

| Read code | Snomed concept id | Medical term |
| --- | --- | --- |
| 1371 | 266919005 | Never smoked tobacco |
| 8CAL | 225323000 | Smoking cessation advice |
| 137S | 8517006 | Ex-smoker |
| 137P | 65568007 | Cigarette smoker |
| 137R | 77176002 | Current smoker |
| 137L | 160618006 | Current non-smoker |
| 137K | 160617001 | Stopped smoking |
| 1374 | 160604004 | Moderate cigarette smoker (10-19 cigs/day) |
| 1371-1 | 8392000 | Non-smoker |
| 1373 | 160603005 | Light cigarette smoker (1-9 cigs/day) |
| 137G | 160616005 | Trying to give up smoking |
| 137T | 160625004 | Date ceased smoking |
| 137 | 266918002 | Tobacco smoking consumption |
| 1379 | 266923002 | Ex-moderate cigarette smoker (10-19/day) |
| 137j | 281018007 | Ex-cigarette smoker |
| 137F | 8517006 | Ex-smoker - amount unknown |
| 1375 | 160605003 | Heavy cigarette smoker (20-39 cigs/day) |
| 137M | 160619003 | Rolls own cigarettes |
| 1378 | 266922007 | Ex-light cigarette smoker (1-9/day) |
| 8H7i | 395700008 | Referral to smoking cessation advisor |
| 9N2k | 401068004 | Seen by smoking cessation advisor |
| 137P-1 | 77176002 | Smoker |
| 137A | 266924008 | Ex-heavy cigarette smoker (20-39/day) |
| 137d | 394873005 | Not interested in stopping smoking |
| 8IAj | 527151000000107 | Smoking cessation advice declined |
| 137X | 230056004 | Cigarette consumption |
| 137c | 394871007 | Thinking about stopping smoking |
| 1372 | 266920004 | Trivial cigarette smoker (less than one cigarette/day) |
| 1377 | 266921000 | Ex-trivial cigarette smoker (<1/day) |
| EGTON320 | 137811000006103 | Smoking Status |
| 137J | 59978006 | Cigar smoker |
| 9OO1 | 185789006 | Attends stop smoking monitoring |
| 137n | 228487000 | Total time smoked |
| 13p0 | 390901002 | Negotiated date for cessation of smoking |
| 137b | 394872000 | Ready to stop smoking |
| 137g | 401201003 | Cigarette pack-years |
| 137I | 43381005 | Passive smoker |
| 137U | 315213009 | Not a passive smoker |
| 13p5 | 401160008 | Smoking cessation programme start date |
| 8IEM | 822591000000108 | Smoking cessation drug therapy declined |
| 137H | 82302008 | Pipe smoker |
| 13WF4 | 161080002 | Passive smoking risk |
| 137B | 266925009 | Ex-very heavy cigarette smoker (40+/day) |
| 13p | 390900001 | Smoking cessation milestones |
| 13p1 | 390902009 | Smoking status at 4 weeks |
| 13WK | 394964001 | No smokers in the household |
| 745H | 710081004 | Smoking cessation therapy |
| EGTONSM3 | 137771000006103 | Smoking Age Started |
| 1372-1 | 428041000124106 | Occasional smoker |
| 67H6 | 506491000000102 | Brief intervention for smoking cessation |
| 9OO | 714001000000108 | Anti-smoking monitoring admin. |
| 137k | 1098881000000100 | Declined to give smoking status |
| 1376 | 160606002 | Very heavy cigarette smoker (40+ cigs/day) |
| 137E | 160614008 | Tobacco consumption unknown |
| 137Q | 266929003 | Smoking started |
| 13p4 | 395177003 | Smoking free weeks |
| 137h | 413173009 | Minutes from waking to first tobacco consumption |
| 745H4 | 713700008 | Smoking cessation drug therapy |
| 8CdB | 783011000000105 | Stop smoking service opportunity signposted |
| E251 | 89765005 | Tobacco dependence |
| 9OOB0 | 783401000000101 | Stop smoking invitation first short message service text message |
| EMISQSM12 | 904181000006102 | Smoking cessation counselling |
| 137C | 160612007 | Keeps trying to stop smoking |
| 67A3 | 171055003 | Pregnancy smoking advice |
| 137-1 | 266918002 | Smoker - amount smoked |
| 137Z | 266918002 | Tobacco consumption NOS |
| EGTONSM4 | 137761000006105 | Smoking Age Ceased |
| EGTON322 | 649861000006105 | Ex-Cigarette Smoker |
| EMISQSM18 | 852131000006108 | Smoking cessation-practice nurse support |
| EMISQSM9 | 904011000006105 | Smoking cessation declaration signed by patient |
| 137m | 446172000 | Failed attempt to stop smoking |
| 137N | 160620009 | Ex-pipe smoker |
| 137O | 160621008 | Ex-cigar smoker |
| ^ESCTTO628233 | 365981007 | Tobacco smoking behaviour - finding |
| EMISHGT222 | 37311000006101 | Other anti-smoking drug given |
| EMISQPR6 | 904021000006102 | Previous smoking quit attempts |
| 137V | 134406006 | Smoking reduced |
| 745Hy | 710081004 | Other specified smoking cessation therapy |
| 1782 | 340921000000103 | Asthma trigger - tobacco smoke |
| 9NdW | 750851000000104 | Consent given for smoking cessation data sharing |
| EGTON321 | 854021000006104 | Cigarette smoker |
| 137W | 81703003 | Chews tobacco |
| 137Y | 230057008 | Cigar consumption |
| 9ko-1 | 505651000000103 | Current smoker annual review |
| 9km-1 | 505761000000105 | Ex-smoker annual review |
| 137K0 | 517211000000106 | Recently stopped smoking |
| EGTON1025 | 604961000006105 | Current Smoker NOS |
| 9OO-1 | 714021000000104 | Stop smoking clinic administration |
| EMISQDA1 | 904091000006100 | Date of last cigarette |
| EMISNQSM6 | 1626121000006100 | Smoking cessation drug therapy - varenicline |
| 137e | 308438006 | Smoking restarted |
| 137f | 401159003 | Reason for restarting smoking |
| 137l | 492191000000103 | Ex roll-up cigarette smoker |
| 9kn-1 | 505681000000109 | Non-smoker annual review |
| EGTON1027 | 649851000006108 | Ex- Rolled Tobacco Smoker |
| 13p50 | 712971000000108 | Practice based smoking cessation programme start date |
| EMISQSM5 | 904151000006105 | Smoking cessation 4 week F/U completed |
| EMISQSM15 | 904221000006106 | Smoking cessation bupropion therapy |
| EMISQSM14 | 904241000006104 | Smoking cessation counselling in person |
| HNGZ011 | 909391000006101 | [RFC] Smoking cessation |
| ASDFGNO1 | 1009271000006100 | Non Smoker - Nos |
| 137I0 | 228524006 | Exposed to tobacco smoke at home |
| 137F-99 | 266928006 | EX-Smoker NOS |
| 137Q-1 | 308438006 | Smoking restarted |
| 137a | 230058003 | Pipe tobacco consumption |
| 177 | 426936004 | Smoke inhalation |
| 137o | 836001000000109 | Waterpipe tobacco consumption |
| 1PD | 908781000000104 | Ex user of electronic cigarette |
| ^ESCTAS810907 | 340921000000103 | Asthma trigger - tobacco smoke |
| EGTON328 | 854151000006107 | Date stopped smoking |
| ^ESCTWA806318 | 203191000000107 | Wants to stop smoking |
| 9kn | 505681000000109 | Non-smoker annual review - enhanced services administration |
| ^ESCTSM822152 | 720401000000103 | Smoking cessation |
| ^ESCTNE549592 | 266919005 | Never smoked |
| 9NdZ | 751661000000106 | Declined consent for smoking cessation data sharing |
| EGTONGR10 | 854951000006109 | Grade A non-smoker |
| EGTONGR11 | 854961000006106 | Grade B light smoker (1-10/day) |
| EGTONGR12 | 854981000006101 | Grade C moderate smoker (11-20/day) |
| EMISQSM4 | 904141000006108 | Smoking cessation 4 week F/U not completed |
| EMISQSM11 | 904201000006101 | Smoking cessation confidence score |
| 137D | 160613002 | Admitted tobacco consumption possibly untrue |
| ^ESCTEX549603 | 266928006 | Ex-cigarette smoker amount unknown |
| EMISCSM1 | 961581000006105 | Smokes/uses tobacco products |
| ZV4K0 | 110483000 | Tobacco user |
| ZV4D7 | 229819007 | Tobacco use and exposure |
| ^ESCT1190499 | 230059006 | Occasional cigarette smoker |
| ^ESCTLI500315 | 230060001 | Light cigarette smoker |
| ^ESCTSM737599 | 449868002 | Smokes tobacco daily |
| E2510 | 89765005 | Tobacco dependence, unspecified |
| 1V08 | 228378005 | Smokes drugs in cigarette form |
| ^ESCTMO500316 | 230062009 | Moderate cigarette smoker |
| ^ESCTHE500317 | 230063004 | Heavy cigarette smoker |
| TDyy4 | 217580001 | Accident caused by cigarette |
| E251z | 89765005 | Tobacco dependence NOS |
| ^ESCTTO395911 | 89765005 | Tobacco dependence syndrome |
| ^ESCTOC815337 | 428041000124106 | Occasional tobacco smoker |
| ^ESCTEX801757 | 48031000119106 | Ex-smoker for more than 1 year |
| EGTON1024 | 852981000006107 | Rolls own cigarettes |
| ^ESCT1172045 | 1092111000000100 | Ex-light smoker (1-9/day) |
| ^ESCT1172048 | 1092131000000110 | Ex-trivial smoker (<1/day) |
| ^ESCTHE342222 | 56771006 | Heavy smoker (over 20 per day) |
| ^ESCTCI407457 | 102408007 | Cigarette smoke |
| E2511 | 191887008 | Tobacco dependence, continuous |
| E2513 | 191889006 | Tobacco dependence in remission |
| 1V09 | 228379002 | Smokes drugs through a pipe |
| ^ESCTSM628235 | 365981007 | Smoking |
| ^ESCT1172039 | 1092071000000100 | Ex-heavy smoker (20-39/day) |
| EMISNQSM16 | 1825071000006100 | Smoking cessation 12 week F/U not completed |
| EGTON324 | 854051000006108 | Ex-pipe smoker |
| ^ESCT1172042 | 1092091000000110 | Ex-moderate smoker (10-19/day) |
| EMISNQSM14 | 1819411000006100 | Smoking increased |
| 137i | 228513009 | Ex-tobacco chewer |
| SMC | 212899006 | Toxic effect of tobacco and nicotine |
| ^ESCTEX319903 | 43381005 | Exposed to tobacco smoke |
| ^ESCTSM354414 | 64197008 | Smoke |
| ^ESCTEX498100 | 228525007 | Exposed to tobacco smoke in public places |
| ^ESCTVE500318 | 230064005 | Very heavy cigarette smoker |
| ZV116 | 8517006 | History of tobacco use |
| E2512 | 191888003 | Tobacco dependence, episodic |
| ^ESCTST736897 | 449369001 | Stopped smoking before pregnancy |
| ^ESCTTO314292 | 39953003 | Tobacco |
| ^ESCTCH500319 | 230065006 | Chain smoker |
| ^ESCTOC549595 | 266920004 | Occasional cigarette smoker (less than one cigarette/day) |
| U271 | 284744004 | [X]Intentional self harm by smoke, fire and flames, occurrence in residential institution |
| ^ESCTCU671807 | 405746006 | Current non smoker but past smoking history unknown |
| EMISQWA1 | 904041000006109 | Waking time to first cigarette |
| EMISQSM16 | 904211000006103 | Smoking cessation service patient satisfaction score |
| ^ESCT1172036 | 1092041000000100 | Ex-very heavy smoker (40+/day) |
| EMISNQSM15 | 1823811000006100 | Smoking cessation 12 week follow up |
| EMISNQUS72 | 1879431000006110 | User of electronic cigarette |
| ^ESCTTO407456 | 102407002 | Tobacco smoke |
| ^ESCTCI558572 | 276468004 | Cigarette burn |
| ^ESCTNE498087 | 228512004 | Never chewed tobacco |
| 137L-99 | 160618006 | Tobacco Consumption Nil |
| EMISQTH1 | 904031000006104 | Thinking about stopping smoking |
| ALLERGY14708NEMIS | 1120481000006110 | Adverse reaction to Lactobacillus Acidophilus |
| J0364 | 9473008 | Tobacco deposit on teeth |
| Eu173 | 90755006 | [X]Mental and behavioural disorders due to use of tobacco: withdrawal state |
| ^ESCTSM494853 | 225934006 | Smokes in bed |
| ^ESCTFI498083 | 228509002 | Finding relating to tobacco chewing |
| ^ESCTAM549590 | 266918002 | Amount and type of tobacco smoked |
| ^ESCTSM642793 | 384742004 | Smoking cessation assistance |
| ^ESCTNE756906 | 702979003 | Never used tobacco |
| ^ESCTCE263604 | 8517006 | Cessation of smoking |
| ^ESCTUS498078 | 228504007 | User of moist powdered tobacco |
| 0C3Z | 159882006 | Tobacco processor NOS |
| ^ESCTCI783250 | 722496004 | Cigarette |
| ^ESCTST334508 | 52138004 | Streptobacillary fever |
| ^ESCTTR500314 | 230059006 | Trivial cigarette smoker |
| ^ESCTAG621728 | 360900008 | Aggressive ex-smoker |
| PCSQI51 | 1538681000006100 | Smoke |
| ^ESCTTO343057 | 57264008 | Tobacco |
| ^ESCTPI387464 | 84498003 | Pipe smoking tobacco |
| ^ESCTTO418033 | 110483000 | Tobacco use |
| ^ESCTDO498075 | 228501004 | Does not use moist powdered tobacco |
| ^ESCTDO498086 | 228511006 | Does not chew tobacco |
| ^ESCTIN621715 | 360890004 | Intolerant ex-smoker |
| ^ESCTTO628237 | 365982000 | Tobacco smoking consumption - finding |
| ^ESCT1179361 | 384742004 | Smoking cessation behaviour support |
| ^ESCTAS765081 | 709507008 | Assessment of readiness for smoking cessation |

**Ethnicity medical codes**

| Read code | Snomed concept Id | Medical term |
| --- | --- | --- |
| 226 | 162730000 | O/E - ethnic group |
| 226-1 | 162730000 | O/E - ethnic origin |
| 9SB1 | 186020007 | Other ethnic, Black/White orig |
| 9SB2 | 186021006 | Other ethnic, Asian/White orig |
| 9SB4 | 186023009 | Other ethnic, other mixed orig |
| 9T | 186034007 | Ethnicity and other related nationality data |
| 9T1 | 186035008 | New Zealand ethnic groups |
| 9SH | 315281001 | Other Asian ethnic group |
| 9S14 | 401214002 | Other white British ethnic group |
| 9iA4 | 86461000000107 | Sri Lankan - ethnic category 2001 census |
| 9i2K | 88971000000106 | Albanian - ethnic category 2001 census |
| 9iF6 | 88991000000105 | Jewish - ethnic category 2001 census |
| 9i4 | 92431000000100 | White and Black African - ethnic category 2001 census |
| 9iA | 92481000000101 | Other Asian background - ethnic category 2001 census |
| 9i22 | 92551000000106 | Welsh - ethnic category 2001 census |
| 9i24 | 92561000000109 | Northern Irish - ethnic category 2001 census |
| 9i63 | 92601000000109 | Chinese and White - ethnic category 2001 census |
| 9iA2 | 92651000000105 | Kashmiri - ethnic category 2001 census |
| 9iA3 | 92661000000108 | East African Asian - ethnic category 2001 census |
| 9iA8 | 92681000000104 | British Asian - ethnic category 2001 census |
| 9iA7 | 92691000000102 | Caribbean Asian - ethnic category 2001 census |
| 9iD1 | 92731000000108 | Nigerian - ethnic category 2001 census |
| 9iF2 | 92771000000105 | Filipino - ethnic category 2001 census |
| 9i26 | 92791000000109 | Cypriot (part not stated) - ethnic category 2001 census |
| 9i27 | 93931000000104 | Greek - ethnic category 2001 census |
| 9i2J | 93981000000100 | Kosovan - ethnic category 2001 census |
| 9i2M | 94001000000108 | Croatian - ethnic category 2001 census |
| 9i2Q | 94021000000104 | Mixed Irish and other White - ethnic category 2001 census |
| 9iFA | 94061000000107 | North African - ethnic category 2001 census |
| 9iF4 | 94131000000103 | Buddhist - ethnic category 2001 census |
| 9iB | 107691000000105 | Caribbean - ethnic category 2001 census |
| 9i20 | 110761000000106 | English - ethnic category 2001 census |
| 9iA6 | 110781000000102 | Sinhalese - ethnic category 2001 census |
| 9i2N | 88981000000108 | Serbian - ethnic category 2001 census |
| 9i64 | 92611000000106 | Asian and Chinese - ethnic category 2001 census |
| 9iD3 | 92721000000106 | Mixed Black - ethnic category 2001 census |
| 9i2B | 93961000000109 | Italian - ethnic category 2001 census |
| 9t1 | 976591000000101 | Ethnic category - 2011 census Northern Ireland |
| 9t01 | 976651000000108 | White: Irish - England and Wales ethnic category 2011 census |
| 9t20 | 977911000000103 | White: Scottish - Scotland ethnic category 2011 census |
| 9t24 | 978011000000101 | White: Polish - Scotland ethnic category 2011 census |
| 9SAD | 186005001 | Other ethnic NEC (NMO) |
| 9T1Z | 186035008 | New Zealand ethnic group NOS |
| ^ESCTOT474038 | 186022004 | Other ethnic, mixed white origin |
| ^ESCTBR474024 | 186006000 | British ethnic minority specified (NMO) |
| ^ESCTBR474026 | 186007009 | British ethnic minority unspecified (NMO) |
| ^ESCTET659190 | 397731000 | Ethnicity |
| ^ESCTET474006 | 185983003 | Ethnic group (1991 census) (UK) |
| ^ESCTET1163867 | 999002401000000000 | Ethnic category - 2011 census Northern Ireland simple reference set |
| ^ESCTET626090 | 364699009 | Ethnic group |
| 9SJ | 372148003 | Ethnic group |
| 9i2H | 88961000000104 | Commonwealth of (Russian) Independent States - ethnic category 2001 census |
| 9i2P | 94011000000105 | Other republics which made up the former Yugoslavia - ethnic category 2001 census |
| 9iFB | 94071000000100 | Middle Eastern (excluding Israeli, Iranian and Arab) - ethnic category 2001 census |
| 9iFJ | 94121000000100 | Multi-ethnic islands: Mauritian or Seychellois or Maldivian or St Helena - ethnic category 2001 census |
| 9t00 | 976631000000101 | White: English or Welsh or Scottish or Northern Irish or British - England and Wales ethnic category 2011 census |
| 9t07 | 976771000000108 | Mixed multiple ethnic groups: any other Mixed or multiple ethnic background - England and Wales ethnic category 2011 census |
| 9t08 | 976791000000107 | Asian or Asian British: Indian - England and Wales ethnic category 2011 census |
| 9t09 | 976811000000108 | Asian or Asian British: Pakistani - England and Wales ethnic category 2011 census |
| 9t0D | 976891000000104 | Black or African or Caribbean or Black British: African - England and Wales ethnic category 2011 census |
| 9t0E | 976911000000101 | Black or African or Caribbean or Black British: Caribbean - England and Wales ethnic category 2011 census |
| 9t0G | 976951000000102 | Other ethnic group: Arab - England and Wales ethnic category 2011 census |
| 9t16 | 977591000000103 | Asian or Asian British: Indian - Northern Ireland ethnic category 2011 census |
| 9t18 | 977731000000108 | Asian or Asian British: Bangladeshi - Northern Ireland ethnic category 2011 census |
| 9t1A | 977771000000105 | Asian or Asian British: any other Asian background - Northern Ireland ethnic category 2011 census |
| 9t1B | 977791000000109 | Black or African or Caribbean or Black British: African - Northern Ireland ethnic category 2011 census |
| 9t1D | 977831000000102 | Black or African or Caribbean or Black British: other Black or African or Caribbean background - Northern Ireland ethnic category 2011 census |
| 9t1F | 977871000000100 | Other ethnic group: any other ethnic group - Northern Ireland ethnic category 2011 census |
| 9t27 | 978071000000106 | Asian or Asian Scottish or Asian British: Pakistani, Pakistani Scottish or Pakistani British - Scotland ethnic category 2011 census |
| 9t2B | 978211000000108 | Asian or Asian Scottish or Asian British: any other Asian group - Scotland ethnic category 2011 census |
| 9t2D | 978251000000107 | African: any other African - Scotland ethnic category 2011 census |
| 9t2H | 978381000000105 | Other ethnic group: Arab, Arab Scottish or Arab British - Scotland ethnic category 2011 census |
| 9t2J | 978401000000105 | Other ethnic group: any other ethnic group - Scotland ethnic category 2011 census |
| 9t04 | 976711000000103 | Mixed multiple ethnic groups: White and Black Caribbean - England and Wales ethnic category 2011 census |
| 9t0A | 976831000000100 | Asian or Asian British: Bangladeshi - England and Wales ethnic category 2011 census |
| 9t0F | 976931000000109 | Black or African or Caribbean or Black British: other Black or African or Caribbean background - England and Wales ethnic category 2011 census |
| 9t17 | 977711000000100 | Asian or Asian British: Pakistani - Northern Ireland ethnic category 2011 census |
| 9t19 | 977751000000101 | Asian or Asian British: Chinese - Northern Ireland ethnic category 2011 census |
| 9t2A | 978191000000109 | Asian or Asian Scottish or Asian British: Chinese - Scotland ethnic category 2011 census |
| 9t2G | 978361000000101 | Caribbean or Black: any other Black or Caribbean group - Scotland ethnic category 2011 census |
| ^ESCTET1163866 | 999002391000000000 | Ethnic category - 2011 census England and Wales simple reference set |
| ^ESCTFI627041 | 365456003 | Finding of ethnicity / related nationality data |
| ESCTBL5 | 315240009 | Black - ethnic group |
| ESCTMI5 | 315239007 | Mixed ethnic census group |
| 9TC | 718958002 | Roma ethnic group |
| 9i2F | 88941000000100 | Polish - ethnic category 2001 census |
| 9i1 | 92401000000106 | Irish - ethnic category 2001 census |
| 9i3 | 92421000000102 | White and Black Caribbean - ethnic category 2001 census |
| 9i21 | 92541000000108 | Scottish - ethnic category 2001 census |
| 9iA5 | 92671000000101 | Tamil - ethnic category 2001 census |
| 9iD0 | 92711000000100 | Somali - ethnic category 2001 census |
| 9i25 | 93921000000101 | Ulster Scots - ethnic category 2001 census |
| 9iFE | 94091000000101 | Kurdish - ethnic category 2001 census |
| 9iF8 | 94141000000107 | Sikh - ethnic category 2001 census |
| 9i0 | 92391000000108 | British or mixed British - ethnic category 2001 census |
| 9SA1 | 186006000 | Brit. ethnic minor. spec.(NMO) |
| 9SB3 | 186022004 | Other ethnic, mixed white orig |
| ^ESCTET659760 | 398089004 | Ethnic background |
| 9T1E | 315280000 | Asian - ethnic group |
| 9S12 | 185984009 | White - ethnic group |
| ^ESCTET822482 | 723621000000103 | Ethnicity |
| ^ESCTON455110 | 162730000 | On examination - ethnic group |
| 9T1Y | 186035008 | Other New Zealand ethnic group |
| ^ESCTET627039 | 365455004 | Ethnic groups (1991 census) (United Kingdom) |
| 226Z | 162730000 | O/E - ethnic group NOS |
| 9t10 | 977351000000100 | White - Northern Ireland ethnic category 2011 census |
| 9t21 | 977931000000106 | White: other British - Scotland ethnic category 2011 census |
| 9i | 92381000000106 | Ethnic category - 2001 census |
| 9iF1 | 92761000000103 | Japanese - ethnic category 2001 census |
| 9i2L | 93991000000103 | Bosnian - ethnic category 2001 census |
| 9i9 | 92471000000103 | Bangladeshi or British Bangladeshi - ethnic category 2001 census |
| 9t13 | 977411000000108 | Mixed multiple ethnic groups: White and Black African - Northern Ireland ethnic category 2011 census |
| 9t15 | 977551000000106 | Mixed multiple ethnic groups: any other Mixed or multiple ethnic background - Northern Ireland ethnic category 2011 census |
| 9t25 | 978031000000109 | White: any other White ethnic group - Scotland ethnic category 2011 census |
| ^ESCTET474007 | 185983003 | Ethnic groups (1991 census) |
| ^ESCTET627040 | 365456003 | Ethnicity / related nationality data - finding |
| ^ESCTOT474034 | 186020007 | Other ethnic, Black/White origin |
| ^ESCTOT474040 | 186023009 | Other ethnic, other mixed origin |
| ^ESCTET659191 | 397731000 | Ethnic groups |
| ^ESCTET627038 | 365455004 | Ethnic groups (1991 census) (UK) |
| 9S | 397731000 | Ethnic group finding |
| 9i2T | 94051000000109 | Other White or White unspecified - ethnic category 2001 census |
| 9t02 | 976671000000104 | White: Gypsy or Irish Traveller - England and Wales ethnic category 2011 census |
| 9t06 | 976751000000104 | Mixed multiple ethnic groups: White and Asian - England and Wales ethnic category 2011 census |
| 9t0H | 976971000000106 | Other ethnic group: any other ethnic group - England and Wales ethnic category 2011 census |
| 9t11 | 977371000000109 | Irish Traveller - Northern Ireland ethnic category 2011 census |
| 9t1C | 977811000000105 | Black or African or Caribbean or Black British: Caribbean - Northern Ireland ethnic category 2011 census |
| 9t23 | 977971000000108 | White: Gypsy or Irish Traveller - Scotland ethnic category 2011 census |
| 9t29 | 978171000000105 | Asian or Asian Scottish or Asian British: Bangladeshi, Bangladeshi Scottish or Bangladeshi British - Scotland ethnic category 2011 census |
| 9t2C | 978231000000100 | African: African, African Scottish or African British - Scotland ethnic category 2011 census |
| 9t2E | 978271000000103 | Caribbean or Black: Caribbean, Caribbean Scottish or Caribbean British - Scotland ethnic category 2011 census |
| 9t2F | 978341000000102 | Caribbean or Black: Black, Black Scottish or Black British - Scotland ethnic category 2011 census |
| 9t0 | 976571000000100 | Ethnic category - 2011 census England and Wales |
| 9t22 | 977951000000104 | White: Irish - Scotland ethnic category 2011 census |
| 9i00 | 494131000000105 | White British - ethnic category 2001 census |
| 9i2E | 88931000000109 | Gypsy/Romany - ethnic category 2001 census |
| 9i8 | 92461000000105 | Pakistani or British Pakistani - ethnic category 2001 census |
| 9i60 | 92581000000100 | Black and Asian - ethnic category 2001 census |
| 9i61 | 92591000000103 | Black and Chinese - ethnic category 2001 census |
| 9iA9 | 92631000000103 | Mixed Asian - ethnic category 2001 census |
| 9iF0 | 92751000000101 | Vietnamese - ethnic category 2001 census |
| 9i2A | 93951000000106 | Turkish Cypriot - ethnic category 2001 census |
| 9i29 | 110401000000103 | Turkish - ethnic category 2001 census |
| 9iF5 | 110831000000107 | Hindu - ethnic category 2001 census |
| ^ESCTET474008 | 185983003 | Ethnic group (1991 census) (United Kingdom) |
| ^ESCTET474042 | 186034007 | Ethnicity / related nationality data |
| 9SZ | 397731000 | Ethnic groups (census) NOS |
| 9SG | 315279003 | Other black ethnic group |
| 9SB | 186019001 | Other ethnic, mixed origin |
| 9i2C | 88911000000101 | Irish Traveller - ethnic category 2001 census |
| 9i2D | 88921000000107 | Traveller - ethnic category 2001 census |
| 9iFH | 89021000000101 | South and Central American - ethnic category 2001 census |
| 9iF7 | 89031000000104 | Muslim - ethnic category 2001 census |
| 9i2 | 92411000000108 | Other White background - ethnic category 2001 census |
| 9i6 | 92451000000107 | Other Mixed background - ethnic category 2001 census |
| 9iC | 92491000000104 | African - ethnic category 2001 census |
| 9iE | 92511000000107 | Chinese - ethnic category 2001 census |
| 9iF | 92521000000101 | Other - ethnic category 2001 census |
| 9i23 | 92571000000102 | Cornish - ethnic category 2001 census |
| 9iA1 | 92641000000107 | Punjabi - ethnic category 2001 census |
| 9iF3 | 92781000000107 | Malaysian - ethnic category 2001 census |
| 9i28 | 93941000000108 | Greek Cypriot - ethnic category 2001 census |
| 9iFG | 94111000000106 | Latin American - ethnic category 2001 census |
| 9i62 | 110771000000104 | Black and White - ethnic category 2001 census |
| 9iD | 92501000000105 | Other Black background - ethnic category 2001 census |
| ^ESCTET1163868 | 999002411000000000 | Ethnic category - 2011 census Scotland simple reference set |
| 9i2R | 94041000000106 | Other White European or European unspecified or Mixed European - ethnic category 2001 census |
| 9iD4 | 92741000000104 | Other Black or Black unspecified - ethnic category 2001 census |
| 9t03 | 976691000000100 | White: any other White background - England and Wales ethnic category 2011 census |
| 9t05 | 976731000000106 | Mixed multiple ethnic groups: White and Black African - England and Wales ethnic category 2011 census |
| 9t0C | 976871000000103 | Asian or Asian British: any other Asian background - England and Wales ethnic category 2011 census |
| 9t1E | 977851000000109 | Other ethnic group: Arab - Northern Ireland ethnic category 2011 census |
| 9t26 | 978051000000102 | Mixed or multiple ethnic groups: any Mixed or multiple ethnic group - Scotland ethnic category 2011 census |
| ^ESCTPE658974 | 397607002 | Perioperative plan of care consistent with patient value system, lifestyle, ethnicity, and culture |
| ^ESCTET636480 | 372148003 | Ethnic category |
| ^ESCTOT474036 | 186021006 | Other ethnic, Asian/White origin |
| 9T1A | 372148003 | Other Pacific ethnic group |
| 9t | 976551000000109 | Ethnic category - 2011 census |
| 9t2 | 976611000000109 | Ethnic category - 2011 census Scotland |
| 9iF9 | 89001000000105 | Arab - ethnic category 2001 census |
| 9iFD | 89011000000107 | Iranian - ethnic category 2001 census |
| 9i5 | 92441000000109 | White and Asian - ethnic category 2001 census |
| 9i2S | 94031000000102 | Other mixed White - ethnic category 2001 census |
| 9iFC | 94081000000103 | Israeli - ethnic category 2001 census |
| 9iFF | 94101000000109 | Moroccan - ethnic category 2001 census |
| 9iFK | 94151000000105 | Any other group - ethnic category 2001 census |
| 9i7 | 110751000000108 | Indian or British Indian - ethnic category 2001 census |
| 9iD2 | 110791000000100 | Black British - ethnic category 2001 census |
| 9SA | 186005001 | Other ethnic non-mixed (NMO) |
| 9SA2 | 186007009 | Brit. ethnic minor. unsp (NMO) |
| 9i10 | 494161000000100 | White Irish - ethnic category 2001 census |
| 9i2G | 88951000000102 | Baltic States (Estonian or Latvian or Lithuanian) - ethnic category 2001 census |
| 9i65 | 92621000000100 | Other Mixed or Mixed unspecified - ethnic category 2001 census |
| 9iAA | 92701000000102 | Other Asian or Asian unspecified - ethnic category 2001 census |
| 9t0B | 976851000000107 | Asian or Asian British: Chinese - England and Wales ethnic category 2011 census |
| 9t12 | 977391000000108 | Mixed multiple ethnic groups: White and Black Caribbean - Northern Ireland ethnic category 2011 census |
| 9t14 | 977431000000100 | Mixed multiple ethnic groups: White and Asian - Northern Ireland ethnic category 2011 census |
| 9t28 | 978111000000100 | Asian or Asian Scottish or Asian British: Indian, Indian Scottish or Indian British - Scotland ethnic category 2011 census |
